# Study on the failure characteristics of sliding surface and stability analysis of inverted t-type retaining wall in active limit state

**DOI:** 10.1371/journal.pone.0298337

**Published:** 2024-02-08

**Authors:** Yongqing Zeng, Weidong Hu, Meixin Chen, Yinghuan Zhang, Xiaohong Liu, Xinnian Zhu

**Affiliations:** 1 College of Civil Engineering and Architecture, Hunan Institute of Science and Technology, Yueyang, China; 2 Nanhu College, Hunan Institute of Science and Technology, Yueyang, China; Jamia Millia Islamia, INDIA

## Abstract

This paper investigates the sliding surface failure characteristics, earth pressure distribution law and stability safety factor of inverted T-type retaining wall by using the finite element limit analysis software OptumG2, the effects of width of wall heel plate, width of wall toe plate, thickness of bottom plate, soil–wall interface friction angle, soil cohesion and soil internal friction angle of filling on the failure characteristics of sliding surface, the earth pressure distribution law and stability safety factor of retaining walls are analyzed, The stability safety factor of the retaining wall showed a gradually increasing trend as the width of wall heel plate and wall toe plate increased; as the bottom plate thickness increases, the stability safety factor of the retaining wall gradually increases; as the soil-wall interface element reduction coefficient rises, that is, the internal friction angle of the soil-wall gradually increases to the soil internal friction angle, the stability safety factor of the retaining wall gradually increases; as the soil cohesion and internal friction angle increase, the stability safety factor of the retaining wall progressively increases. The safety factor of retaining wall increases by 0.45 for every 0.5m increase in the width of the wall heel plate; the safety factor of the retaining wall increases by 0.29 when the width of the wall toe plate increases by 0.5m; for every 0.5m increase in the width of wall plate thickness, the safety factor of the retaining wall is increased by 0.62; for every 0.25 increase in soil-wall interface element reduction coefficient, the safety factor of the retaining wall increases by 0.29; for every increase of 5KPa in soil cohesion, the safety factor of the retaining wall increased by 1.16; for every 5° increases in soil internal friction angle, the safety factor of retaining wall increases by 0.6. The research is significant for studying the failure laws and stability of retaining walls and providing references for retaining wall design.

## 1. Introduction

Retaining walls refer to structures that support high-filling subgrade or hillside soil to prevent deformation and instability; it is widely used in civil engineering, water resources and hydropower engineering, highway and railway engineering [1–4]. During the construction process or after transferring the completed retaining wall, there are often a variety of damages or even large-scale collapse. In the construction process and improper maintenance, the retaining wall may be damaged, but the damage caused by imperfect design is a more general pattern for some retaining walls.

The inverted T-type retaining wall is a retaining structure constructed of reinforced concrete materials, consisting of three parts: vertical arm panel, toe plate, and heel plate. It relies on the wall body self-weight and the filling soil’s gravity above the wall bottom plate to maintain the stability of the retaining wall [5, 6]. It has the characteristics of small thickness, light self-weight, and large height and is suitable for filling sections with insufficient stone materials and low foundation-bearing capacity.

**Fig 1:**
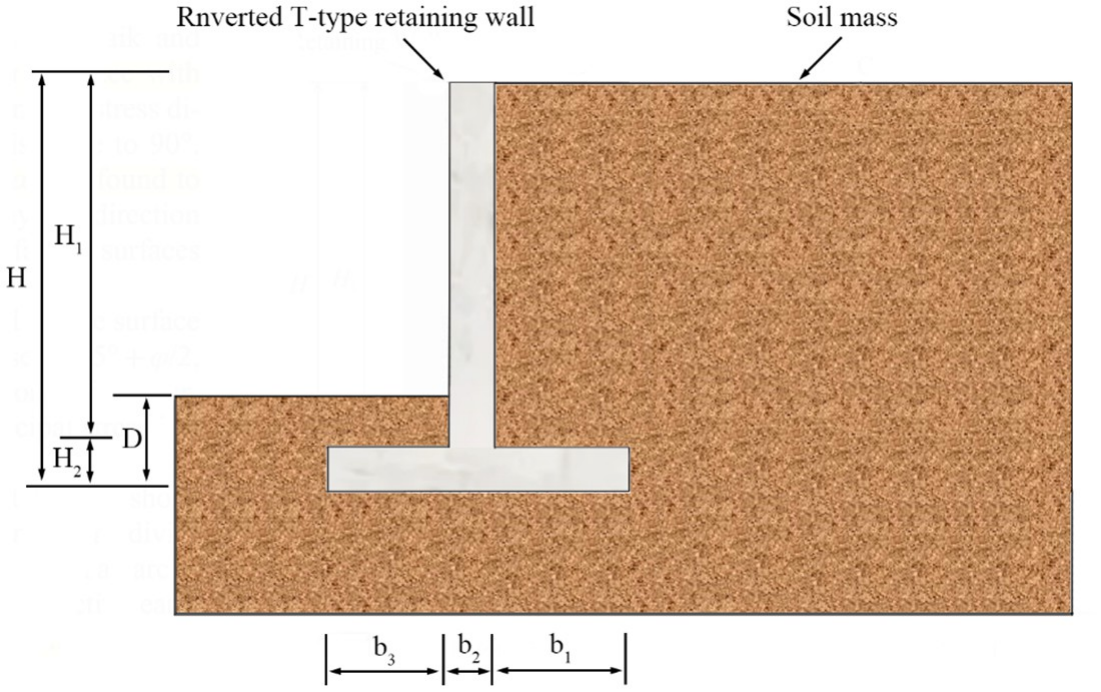
The geometry section of the inverted T-Type retaining wall.

Inverted T-type retaining wall mainly relies on the wall stem and the backfill on the bottom plate to resist earth pressure. The deformation and failure characteristics of the sliding surface, earth pressure distribution law, and stability safety factor in the backfill behind inverted T-type retaining walls differ from other types of retaining walls. The geometry section of the inverted T-type retaining wall is shown in Fig 1, in which the retaining wall’s height is H; the wall stem’s height is H_1_; the bottom plate thickness is H_2_; D is the buried depth at the bottom of the wall; the wall heel width is b_1_; the wall stem is entirely vertical with a width of b_2_; and the wall toe width is b_3_.

In general, the inverted T-Type retaining wall design needs to check the anti-sliding stability, anti-overturning stability, wall section strength, and foundation bearing capacity. In this process, Rankine earth pressure theory or Coulomb earth pressure theory is usually used to calculate earth pressure. Rankine earth pressure theory assumes that the back of the retaining wall is smooth upright, and the filling soil surface is horizontal. In contrast, Coulomb earth pressure theory assumes that the filling behind the retaining wall is an ideal granular body, the sliding failure surface is a plane, and the sliding soil is a rigid body. However, these assumptions differ from the actual situation, so sometimes the calculation results bring a significant error [7, 8]. In particular, analyzing retaining wall stability using traditional methods is more challenging when the retaining wall structure is varied, and the geological and engineering conditions are complicated [9–11]. In the design of an inverted T-Type retaining wall, larger cross-sectional dimensions are often encountered to meet the stability requirements of the retaining wall, resulting in unnecessary waste of resources. Therefore, it is necessary to analyze factors affecting the stability of the retaining wall and provide a theoretical basis for optimizing the section design of the retaining wall.

For analysis and research on retaining wall stability, the main drawback of the conventional stability analysis for retaining walls is examined, wherein the irrational premise of its theoretical analysis is identified. A more rational overturning coefficient K_0_ is proposed, considering the assumption that the vertical load on the retaining wall is equivalent to the actual loading state at the limit condition. By comparing the bearing capacity and overturning stability requirements outlined in China’s design guidelines, it is established that as long as the foundation’s bearing capacity fulfills the design specifications, overturning stability can be ensured, thereby eliminating the need for an individual examination of overturning stability [12]. When a retaining wall is adjacent to a natural slope, the primary cause of wall overturning is typically a yielding foundation. At the point of overturning, the narrow backfill behind the wall has reached its active limit state. However, previous studies did not adequately consider the foundation conditions when calculating the earth pressure on the retaining wall with the narrow backfill. In order to comprehensively assess the stress state of the retaining wall, the finite-element limit analysis method was employed to investigate the failure mode of a retaining wall adjacent to a natural slope; a slip-line computational model for the retaining wall was established. The shape of the narrow backfill, the roughness of the soil-wall interface, and the use of low-strength backfill can all contribute to the reduction of the active earth pressure on a retaining wall [13]. A slip-line method investigated the active earth pressure of narrow and layered backfills against an inverted T-type retaining wall rotating around the base mode. The simulation used adaptive finite-element software to represent the failure mechanism accurately. Slip-line field calculation models were established for the inverted T-type retaining wall to study the characteristics of the narrow and layered backfills and the effect of the heel bottom plate. Each point’s stress state was then determined using the limit equilibrium and finite difference methods. The earth pressure acting on the stem and the second failure surface could be obtained by converting the boundary conditions. The results of the proposed method were compared with those obtained from finite-element limit analysis and existing theoretical solutions [14]. It is challenging to design and assess retaining walls due to disagreements regarding how earthquakes affect the toe of retaining walls in design codes in China and other countries. The study focuses on a retaining wall with a 2m soil layer covering its front, aiming to design and analyze its response to strong earthquake motion effectively. The shear forces acting on the toe of the wall play a crucial role in maintaining dynamic stability and resisting overturning and sliding caused by dynamic earth pressure. By employing a simplified method, the resultant forces from pressure and shear on both the toe and the back of the wall are determined and found to be close. To promote accurate design, a reliable value for the earthquake impact on the toe of the wall is proposed, and the reduced coefficient of seismic action effect is discussed [15]. The adaptive finite element analysis explores narrow cohesive backfill failure mechanisms under seismic conditions. A pseudo-static solution is developed where a narrow cohesive backfill is present behind a gravity retaining wall and experiences seismic requirements. An analytical solution is obtained using the limit equilibrium method, Rankine’s theory, and the horizontal differential layer method. Moreover, a novel approach is proposed, which utilizes the finite difference method to determine the depth of tension cracks in the narrow cohesive backfill. Additionally, an iterative calculation is used to determine the critical failure angle of the backfill wedge [16]. In order to investigate the response of a small-scale physical model of a retaining wall to high-intensity acceleration time history, 12 shake table experiments were conducted on a 0.4 m high physical model. These experiments simulated three different intensities of base acceleration, representing peak ground acceleration of various historical earthquakes ranging from 0.74g to 1.44g. The study considered a wide range of relative density, ranging from 35% to 55%, with a step size of 10%. In the denser cases, both static and dynamic loadings exhibited up to 48% and 54% less lateral displacement, respectively. Moreover, displacements increased by 39% under higher peak ground acceleration. The presence of vertical surcharge contributed to a significant increase in lateral movement of the wall; an increase of 92% and 142% was observed for static and dynamic cases, respectively [17].

For analysis and research on earth pressure distribution and failure mechanisms of retaining wall, it is hypothesized that a linear relationship exists between the lateral pressure and horizontal displacement along a rigid retaining wall. The soil mass is considered as a combination of springs and ideal rigid plasticity objects. By analyzing displacement models of the retaining wall and enhancing Coulomb’s theory, a calculation method for active earth pressure on rigid retaining walls under various displacement modes is proposed, thereby offering theoretical insight into studying active earth pressure on rigid retaining walls [18]. A series of translation and rotation experiments were conducted using a model box to investigate the distribution pattern of non-limit passive earth pressure of clay on a rigid retaining wall at varying depths under different displacement modes. The aim was to analyze the impact of these displacement modes and corresponding displacements on the lateral earth pressure. The analysis revealed that the resultant force of lateral earth pressures exhibited an increasing trend with the compressive displacement for all three modes: translation, rotation around the top, and rotation around the bottom. Notably, the translation mode showed the highest increase magnitude [19]. An experiment was conducted to investigate the active earth pressure of cohesionless soil behind a retaining wall with limited width in three different modes: translational motion, rotation around the bottom, and rotation around the top. The experiment aimed to observe the failure modes of the soil and the distribution of the active earth pressure with varying backfill widths. The results indicated that the failure surface of the limited-width soil behind the retaining wall is continuous and progressively moves outward, eventually reaching a fixed position as the backfill width increases. Moreover, Coulomb’s theory is no longer applicable to the backfilled soil with limited width. Additionally, it was found that the motion patterns of the retaining wall significantly influence the distribution of the active earth pressure [20]. To investigate the impact of retaining wall movement mode and fill width on the active earth pressure of cohesionless finite soil, it is conducted discrete element simulations involving the translation (T) mode, rotating about the base (RB) mode, and rotating about the top (RT) mode of the retaining wall, which were performed using different fill widths. Variations in the distribution of active earth pressure can be observed by observing the soil’s failure mode and stress state as influenced by the retaining wall’s movement mode and the fill width. In the T and RB modes, the values of the mobilized internal friction angle within the sliding wedge increased when compared to the initial value. Additionally, small principal stress arches manifested in the sliding soil wedges of the T mode. However, The RT mode exhibited a more unique behaviour. When the fill width was small, the stress state closely resembled the T mode’s. However, when the fill width was large, the upper part of the sliding wedge displayed a region where the internal friction angle decreased relative to the initial value, accompanied by the appearance of a significant principal stress arch [21]. A new visual model was utilized to investigate the failure mechanism and earth pressure of cohesionless narrow backfill positioned behind a rigid retaining wall. The experimental system can simulate various displacement modes of the retaining wall, the wall’s inclinations, and the backfill widths. The results revealed that when the retaining wall moves through a translation mode, a sliding surface forms as a reflection between the walls and reaches the ground. Furthermore, it was observed that as the width of the backfill decreases, the number of reflective sliding surfaces increases. The active earth pressure on the wall was found to be lower than the values obtained through classical theories and follows a nonlinear distribution. It was also established that the number of reflective sliding surfaces can be mitigated by increasing the inclination of the retaining wall [22]. To explore the active earth pressure on inverted T-type retaining walls under rotational mode, a slip-line method is employed to simulate the typical failure mechanism using adaptive finite element software. In light of the heel bottom plate’s influence, the slip-line field calculation models are established for inverted T-type retaining walls with both long and short heels. The stress state of each point is determined using the limit equilibrium method and the finite difference method. By converting the boundary conditions, the earth pressure acting on the stem and imaginary wall can be determined. The findings indicate that increasing the length of the heel and enhancing the interface strength are advantageous in maintaining the integrity of the backfill and reducing the earth pressure exerted on the stem and imaginary wall [23]. In the field of high seismic intensity, there have been limited methods available for calculating active earth pressure (Ea), particularly for cantilever retaining walls with long relief shelves (CRW-LRS). Therefore, the seismic active earth pressure (Es) on CRW-LRS structures was investigated subjected to translational mode. The finite element method (FEM) was employed to assess the failure mode of the active limit state under seismic loading. The findings indicate that the backfill located behind the wall generates the first sliding surface at the bottom of the wall heel, the second at the top of the wall heel, and the third at the top of the relief shelf. Consequently, a calculation formula for Es under the long relief shelf failure mode was proposed by utilizing the limit analysis method of a horizontal differential layer [24]. The proposed method utilizes failure surfaces to analyze the active lateral earth thrusts exerted on cantilever retaining walls. Using the limit-equilibrium approach, a new method for determining lateral earth thrusts on cantilever walls with either a short or long heel is proposed. To determine the active earth thrust coefficients and failure surface inclination angles behind a cantilever wall in an active scenario, an earth thrust-maximization algorithm was developed and implemented using Matlab Environment. Additionally, particle image velocimetry (PIV) analysis was employed to experimentally examine the failure surfaces behind model cantilever walls and corresponding failure cases. Through both analytical and experimental investigations, the effects of parameters on long-heel and short-heel cases were thoroughly examined [25].

Inverted T-type retaining walls are widely used in civil engineering. Accurate determination of failure characteristics of the sliding surface, active earth pressure distribution law and stability analysis of inverted T-type retaining wall in the active limit state is of immense importance for designing earth retaining structures. With the development of numerical calculation technology, using numerical models to analyze the stability of retaining wall structures has become an effective approach. Therefore, the finite element limit analysis software OptumG2 is used to analyze the deformation and failure characteristics of the sliding surface, earth pressure distribution law in the backfill and stability safety factor of retaining wall; the effects of width of wall heel plate, width of wall toe plate, thickness of bottom plate, soil–wall interface friction angle, soil cohesion and soil internal friction angle of filling on the failure characteristics of sliding surface, the earth pressure distribution law and stability safety factor of retaining walls are analyzed, which provides a reference for the design and application of the inverted T-Type retaining wall.

## 2. Failure mechanism analysis against inverted T-type retaining wall

### 2.1. Finite-Element model

#### (1) Introduction to finite element limit analysis software OptumG2

OptumG2 is advanced geotechnical analysis software that combines two-dimensional finite element analysis with limit analysis. It offers a range of features, including simple operation, fast modeling, support for CAD file import, and automatic grid encryption. OptumG2 is particularly effective in analyzing complex geological conditions, conducting failure mode analysis of intricate retaining structures, assessing foundation bearing capacity, and performing reliability analysis [26, 27]. One of the critical strengths of OptumG2 lies in its computational core, which is based on advanced numerical algorithms. Unlike ordinary finite element programs, OptumG2 does not encounter common issues such as convergence problems; therefore, there is no need to spend time adjusting algorithm parameters. Instead, one can focus on solving the underlying physical issues. OptumG2 streamlines the process by calculating strict upper and lower limits for the desired physical quantities. Users can immediately estimate the exact solution and error range by utilizing these bounds. Additionally, increasing the number of elements in the calculation further enhances the accuracy of both the solution and the error estimation. OptumG2 also offers adaptive grid encryption for analysis, providing a powerful method to achieve high accuracy at a low computational cost.

#### (2) Finite element analysis and boundary state

**Fig 2:**
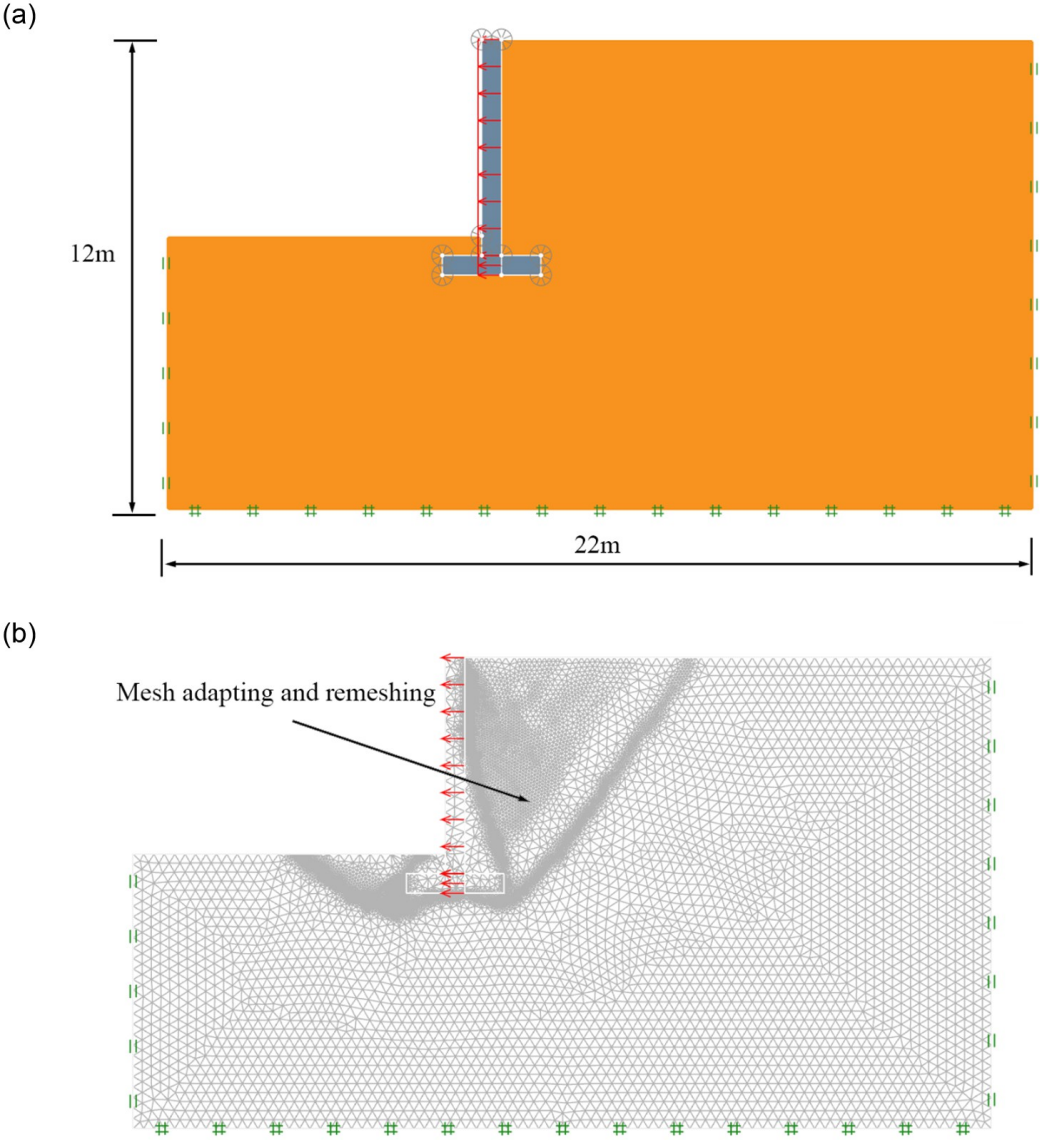
(a). The finite-element model. (b). adaptivity mesh.

This paper used finite element limit analysis software OptumG2 to simulate the active failure mechanism of an inverted T-type retaining wall in active limit state. The finite element model and adaptivity mesh are shown in Fig 2; to eliminate boundary effects, the finite element model dimensions are set as 22 m and 12 m in the horizontal direction and vertical direction, respectively. The left and right sides of the model are constrained in normal direction, the bottom of the model is constrained in tangential and normal direction, and the upper boundary of the model is free. A uniform lateral distributed load with a multiplier is applied to the whole section of the retaining wall to simulate the translation process of the retaining wall. The uniform lateral distributed load gradually increases until the backfill soil reaches the active limit state. It is possible to adapt the mesh in a series of adaptivity iterations. For an accurate representation, the retaining wall is simulated by using the rigid element with unit weight *γ* = 25KN/m^3^ and the soil mass is simulated with the Mohr–Coulomb model, the retaining wall’s height H = 6m; the wall stem’s height H_1_ = 5.5m; the bottom plate thickness H_2_ = 0.5m; the buried depth at the bottom of the wall D = 1m; the wall heel width b_1_ = 1m; the wall stem is entirely vertical with a width of b_2_ = 0.5m; and the wall toe width is b_3_ = 1m.

#### (3) Numerical calculation settings and material parameters

The lower bound limit elements are chosen in the finite element model, the initial mesh consisted of 20000 elements; the mesh was redistributed through adaptive iterations to achieve a more precise mesh distribution. Three adaptive iterations are carried out by considering shear dissipation as the control variable, and the total number of iterative mesh elements is set to 25000.

The Mohr-Coulomb model is used to simulate the soil in the finite element model. The basic Mohr-Coulomb yield function is given by

$$F=\left|\sigma_{1}\text{-}\sigma_{3}\right|\text{+}\left(\sigma_{1}\text{-}\sigma_{3}\right)\text{sin}\varphi -2c\text{cos}\varphi$$ (1)

Where *σ*_1_ and *σ*_3_ are the major and minor principal stresses respectively; *c* is soil cohesion, φ is soil internal friction angle.

The main parameters of the backfill are provided in Table 1. The soil in the finite element model is assumed to be in a normally consolidated state. The dilatancy angle of the soil is set to 0, and the soil follows the non-correlated flow criterion. The soil–wall interface friction angle of δ is simulated using the soil–wall interface element reduction coefficient K and can be calculated using the following equation:

δ=Kφ (2)

where *δ* is soil–wall interface friction angle, K is soil–wall interface element reduction coefficient and *φ* is soil internal friction angle.

**Table 1:** The main calculation parameters of soil mass.

Name of soil layer	Natural unit weight(kg/m^3^)	Cohesion (KPa)	Internal friction angle (°)	young’s modulus (MPa)	Poisson’s ratio
Firm clay	21	15	20	25	0.30

### 2.2. Parametric research scheme of influencing factors

The single factor analysis method is used to study the effects of inverted T-type retaining wall structure dimensions and mechanical parameters of filling, in which the width of the heel bottom plate b_1_, the width of bottom plate b_3_, the bottom plate thickness H_2_, the soil–wall interface element reduction coefficient K, the soil interface friction angle φ and the soil cohesion C are selected influencing factors for the failure characteristics of sliding surface and the earth pressure distribution law of retaining wall in active limit state. Geometric dimensions of the retaining wall and mechanical parameters for working conditions are shown in Table 2, in which the retaining wall’s height H = 6m, the wall stem is entirely vertical with a width of b_2_ = 0.5m, the buried depth at the bottom of the wall D is always greater 0.5m than the bottom plate thickness; a correlation of the investigated range of soil parameters to conventional values for retaining wall design would be insightful; the values of soil parameters are determined according to the engineering geological manual on soil properties [28] and other relevant retaining wall papers [29–32]. In Group 1, the wall heel width b_1_ is 1m, 2m, 3mand 4m, respectively; In Group 2, the wall toe width b_3_ is 1m, 2m, 3m and 4m, respectively; In Group 3, the bottom plate thickness H_2_ is 0.5m, 0.75m, 1.0m and 1.25m, respectively; In Group 4, the soil–wall interface element reduction coefficient K is 0, 0.333, 0.666 and 1, respectively; In Group 5, the soil cohesion C is 5KPa, 15KPa, 25KPa and 35KPa, respectively; In Group 5, the soil internal friction angle φ is 10°, 20°, 30° and 40°, respectively. In addition, during the analysis, the young’s modulus E is 25MPa and the poisson’s ratio is 0.3.

**Table 2:** Geometric dimensions and mechanical parameters for working conditions.

Working conditions	Wall heel width b_1_ (m)	Wall toe width b_3_ (m)	Bottom plate thickness H_2_(m)	Soil–wall interface element reduction coefficient K	Soil cohesion C (KPa)	Soil internal friction angle φ(°)
Group 1	1~4	1	0.5	1	15	20
Group 2	1	1~4	0.5	1	15	20
Group 3	1	1	0.5~1.25	1	15	20
Group 4	1	1	0.5	0~1	15	20
Group 5	1	1	0.5	1	5~35	20
Group 6	1	1	0.5	1	15	10~40

### 2.3. Calculation principle of stability analysis

Limit Analysis allows for the rapid assessment of geostructures’ stability or bearing capacity without having to perform an exhaustive step-by-step elastoplastic analysis.

Multiplier load is applicable in Limit Analysis; the load is amplified until a state of incipient collapse is attained; the factor by which the multiplier load needs to be amplified in order to cause collapse is also referred to as the collapse multiplier [33]. In OPTUM G2, stability analysis can be carried out using Limit Analysis with Multiplier. In cases, collapse multiplier less than 1 implies collapse, while a value of collapse multiplier above 1 implies that the system is stable, in which the collapse multiplier is the safety factor.

The criteria for retaining wall stability calculation and judgment refer to the national standard "Technical Code for Construction Slope Engineering" (GB50330-2013) [34], as shown in Tables 3 and 4. If a safety accident occurs, the consequences are very serious; therefore, the safety grade of slope engineering is First order; under normal conditions, the critical safety factor of slope Fst should be taken as 1.35.

**Table 3:** Classification of slope stability states.

Slope stability coefficient F_s_	*F*_*s*_ < 1.00	1.00 ≤ *F*_*s*_ < 1.05	1.00 ≤ *F*_*s*_ < *F*_*st*_	*F*_*s*_ ≥ *F*_*st*_
Slope stability states	Instable	Understable	Basically stable	Stable

**Table 4:** Critical safety factor of slope *F*_st_.

Slope type		Safety grade of slope engineering		
		First order	Second order	Third order
Permanent slope	Normal condition	1.35	1.30	1.25
	Seismic condition	1.15	1.10	1.05
Temporary slope		1.25	1.20	1.15

## 3. Results and discussion

### 3.1. The failure characteristics of sliding surface

The results of finite element limit analysis can be presented in the form of shear dissipation contours; a shear dissipation contour represents the potential sliding surface; the greater the shear dissipation energy, the greater the possibility of shear in this region.

**Fig 3:**
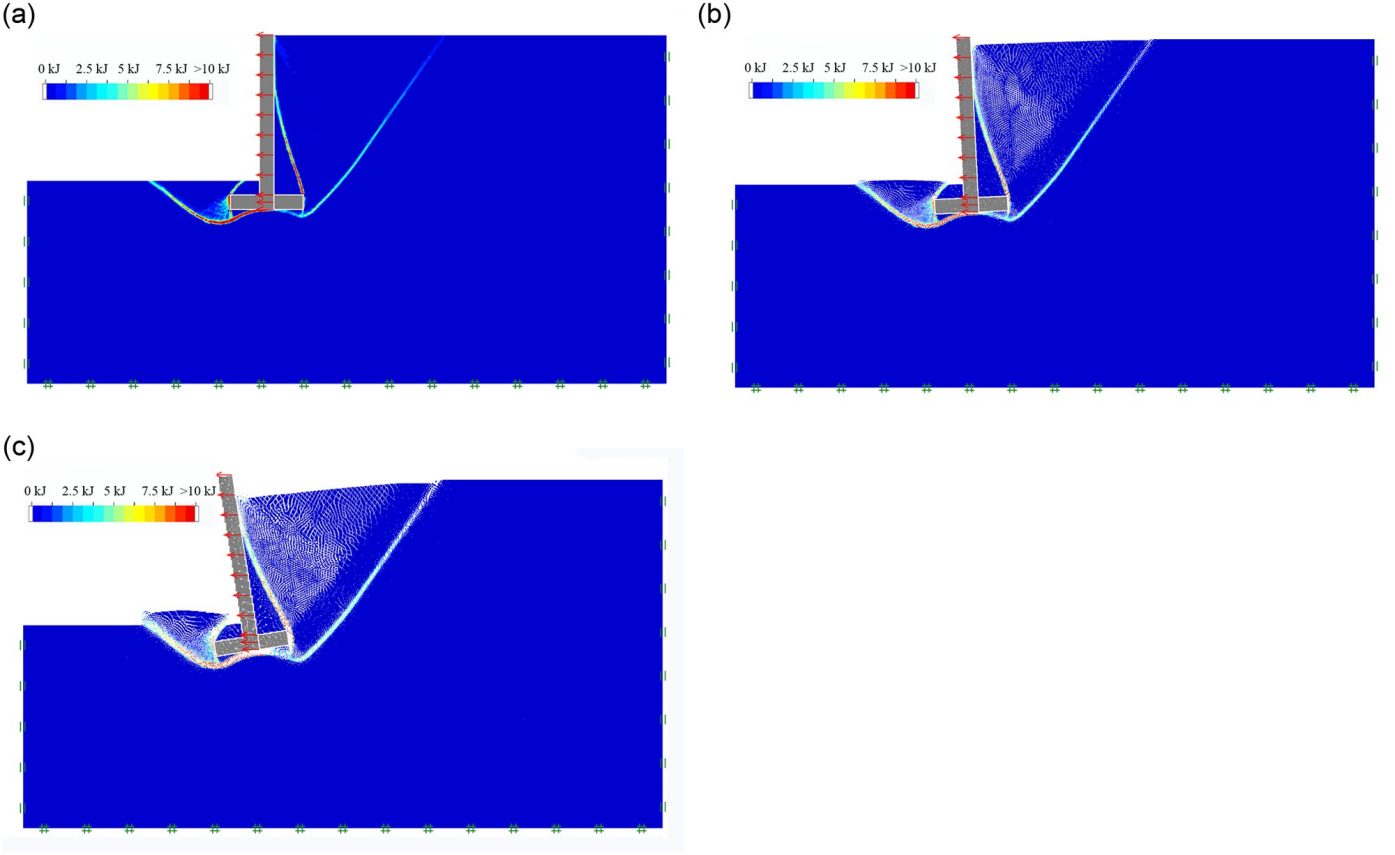
Sliding process of inverted T-type retaining wall in active limit state. (a) sliding surface; (b) sliding process; (c) final failure state.

When the retaining wall’s height H = 6m, the wall stem is entirely vertical with a width of b_2_ = 0.5m, the buried depth at the bottom of the wall D = 0.5m, the wall heel width b1 = 1m, the wall toe width b_3_ = 1m, the bottom plate thickness H_2_ = 0.5m, the soil–wall interface element reduction coefficient K = 1, the soil parameters are selected with shown in Table 1, The sliding process of inverted T-type retaining wall in the active limit state is described in Fig 3; It can be seen that there are two sliding failure surfaces developed in the soil behind the retaining wall in an active state; these two failure surfaces are called the first and second failure surfaces behind the wall, respectively; The first failure surface developed from the lower edge of the wall bottom plate to the ground and the second failure surface formed from the upper edge of the wall bottom plate to the wall stem. A sliding failure surface developed from the bottom of the wall stem to the ground appeared in the soil in front of the wall; the retaining walls exhibit a characteristic of overturning failure.

#### (1)The effect of wall heel width

**Fig 4:**
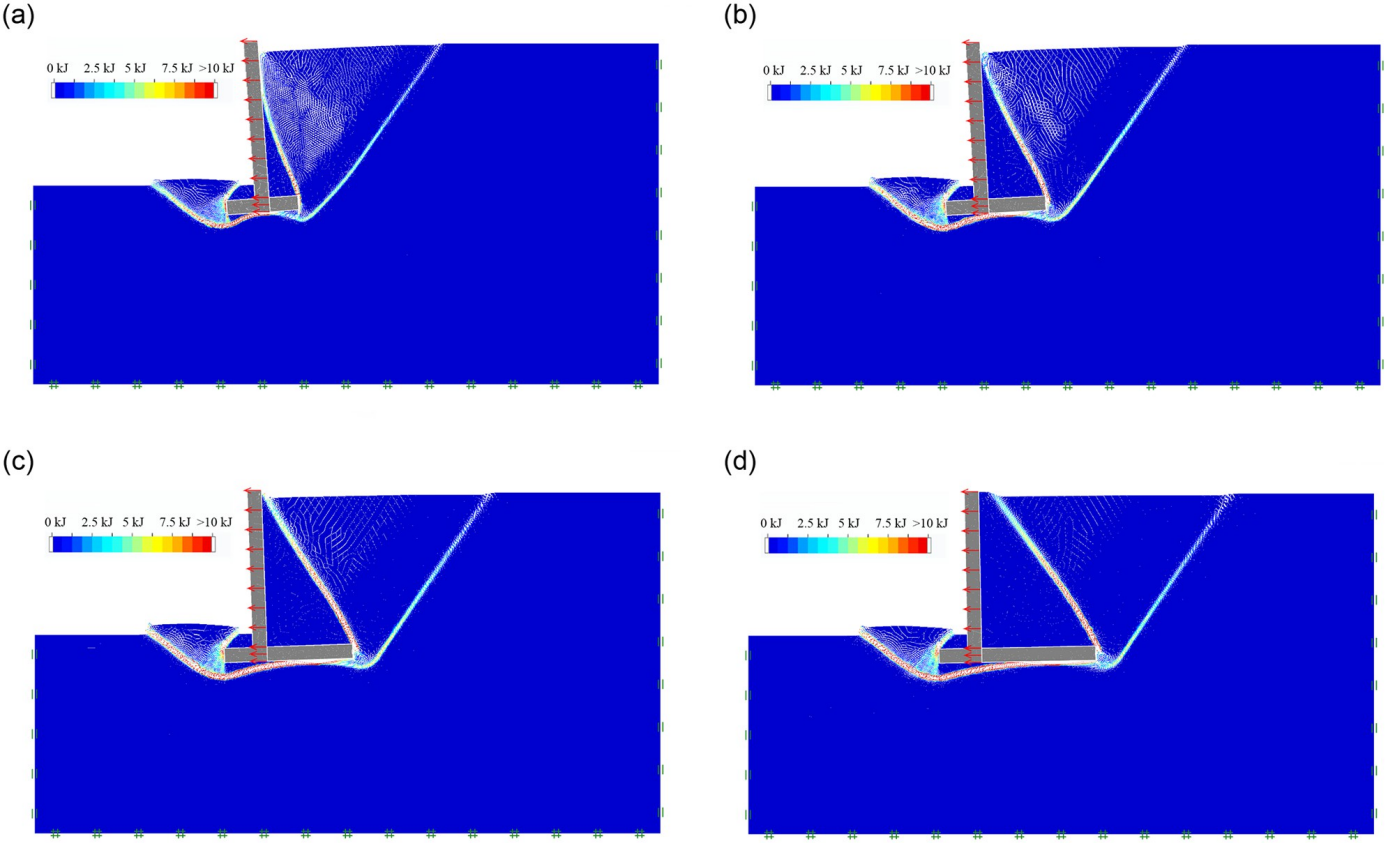
The soil mass final failure state of wall heel width. (a) b_1_ = 1m; (b) b_1_ = 2m; (c) b_1_ = 3m; (d) b_1_ = 4m.

As the soil mechanical parameters and the geometric dimension of inverted T-type retaining walls are shown in Tables 1 and 2, For Group 1, when the wall heel width b_1_ is 1m, 2m, 3m, and 4m, respectively, the soil mass final failure state of retaining wall in active limit state is shown in Fig 4; the soil mass sliding surface, sliding process and final failure state is included in the S1 Appendix. It can be seen that as the wall heel width b_1_ increases from 1m to 4m, the soil behind the inverted T-shaped retaining wall still develops the first and second failure surfaces behind the wall; The first failure surface always extends from the lower edge of the wall bottom plate to the ground, while the second failure surface changes from extending from the upper edge of the wall bottom plate to the wall stem and directly extending from the upper edge of the wall stem plate to the ground. A sliding failure surface in the soil in front of the wall still develops from the bottom of the wall stem to the ground. When the wall heel width b_1_ is 1m, the ratio of wall heel width to the wall stem’s height is 0.181; under the limit horizontal load, the retaining wall has the characteristic of overturning failure; As the wall heel width b_1_ gradually increases to 4m, the ratio of wall heel width to the wall stem’s height is 0.727; under the limit horizontal load, the retaining wall is characterized by sliding failure.

#### (2)The effect of wall toe width

**Fig 5:**
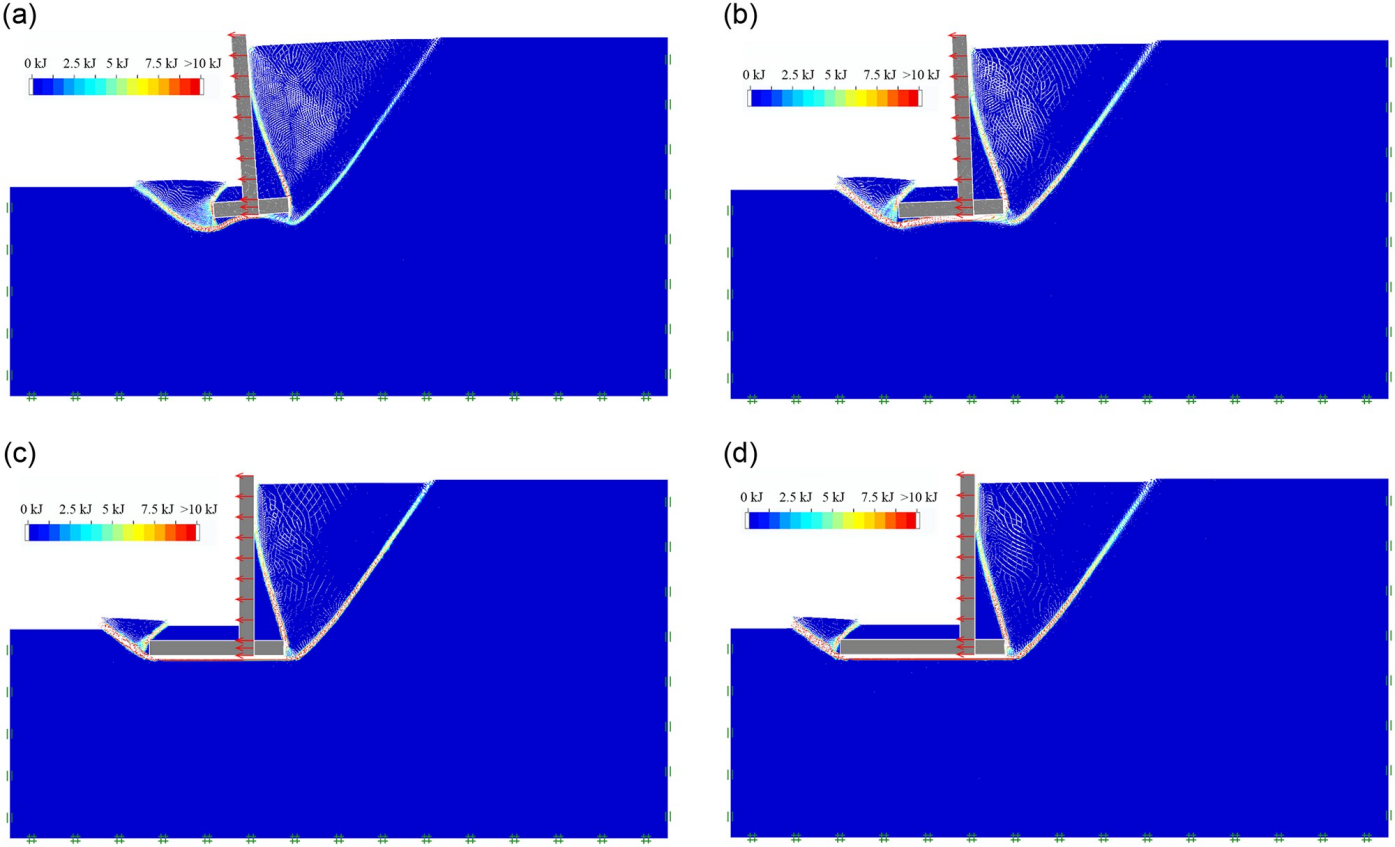
The soil mass final failure state of wall toe width. (a) b_3_ = 1m; (b) b_3_ = 2m; (c) b_3_ = 3m; (d) b_3_ = 4m.

As the soil mechanical parameters and geometric dimension of inverted T-type retaining walls are shown in Tables 1 and 2, For Group 2, when the wall toe width b_3_ is 1m, 2m, 3m, and 4m, respectively, the soil mass final failure state of retaining wall in active limit state is shown in Fig 5; the soil mass sliding surface, sliding process and final failure state is included in the S1 Appendix. It can be seen that as the wall toe width b_3_ increases from 1m to 4m, the soil behind the inverted T-shaped retaining wall still develops the first and second failure surfaces behind the wall; The first failure surface extends from the lower edge of the wall bottom plate to the ground, and the second failure surface extends from the upper edge of the wall bottom plate to the wall stem. A sliding failure surface in the soil in front of the wall changes from developing from the bottom of the wall stem to the ground to directly extending from the lower edge of the wall stem plate to the ground. When the wall toe width b_3_ is 1m, the ratio of wall toe width to the wall stem’s height is 0.181; under the limit horizontal load, the retaining wall has the characteristic of overturning failure; As the wall toe width b_3_ gradually increases to 4m, the ratio of wall toe width to the wall stem’s height is 0.727; the retaining wall observably shows the characteristic of sliding failure.

#### (3)The effect of bottom plate thickness

**Fig 6:**
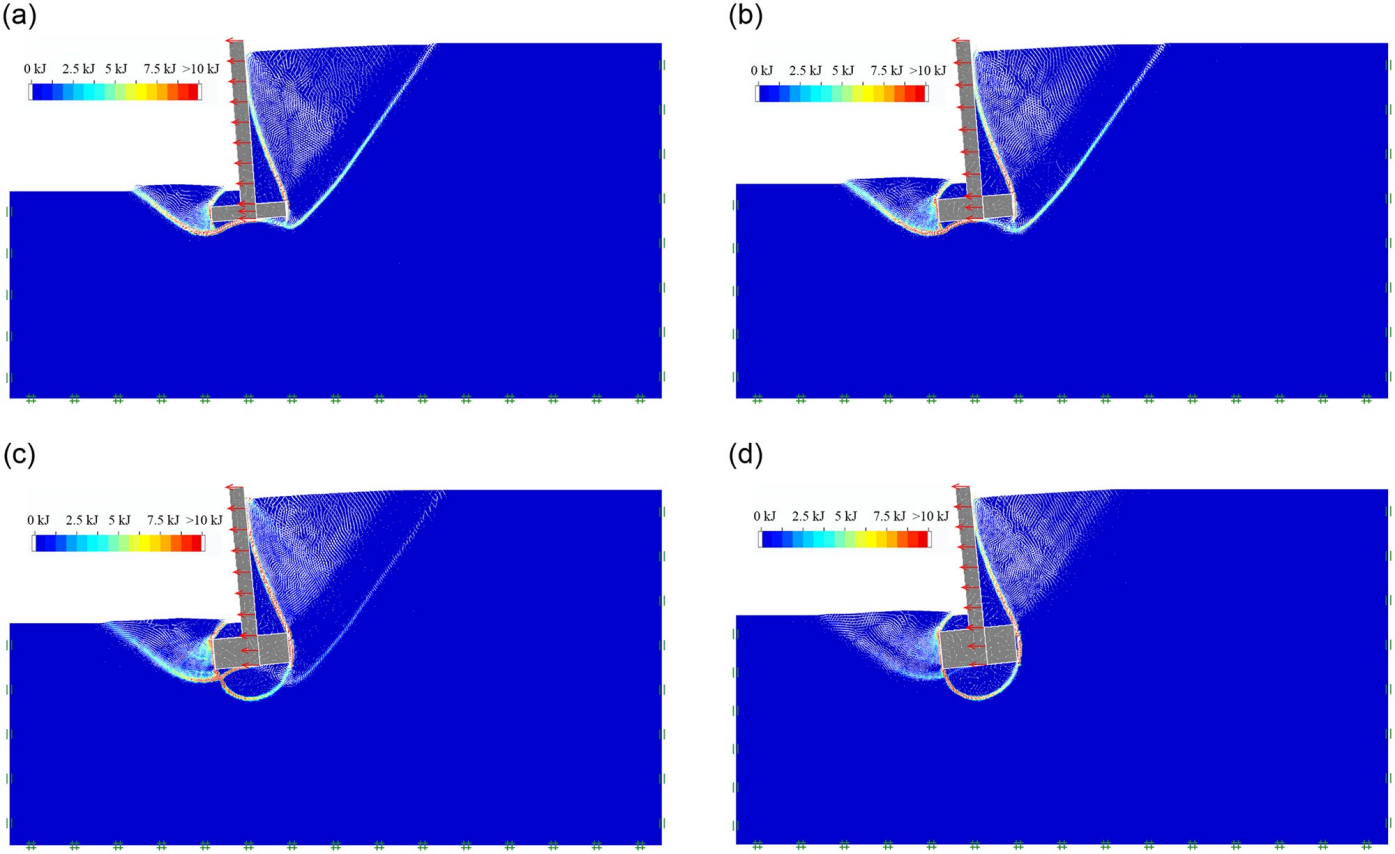
The soil mass final failure state of bottom plate thicknesss. (a) H_2_ = 0.5m; (b) H_2_ = 0.75m; (c) H_2_ = 1.0m; (d) H_2_ = 1.25m.

As the soil mechanical parameters and geometric dimension of inverted T-type retaining walls are shown Tables 1 and 2, For Group 3, when the bottom plate thickness H_2_ is 0.5m, 0.75m, 1.0m and 1.25m, respectively, the soil mass final failure state of retaining wall in active limit state is shown in Fig 6; the soil mass sliding surface, sliding process and final failure state is included in the S1 Appendix. It can be seen that as the bottom plate thickness H_2_ increases from 0.5m to 1.25m, the retaining wall apparently shows the characteristic of overturning failure. The first failure surfaces behind the wall changes from the state that develops from the lower edge of the wall bottom plate to the ground to the state that extends from the upper edge of the wall bottom plate to the ground, showing a gradual disappearance feature, while the second failure surfaces behind the wall still exists; The soil below the bottom plate forms a arc failure surface from the lower edge of the wall toe to the lower edge of the wall heel and extends to the second sliding surface; The soil in front of the wall exhibits a trend of varying from one obvious fracture facing to multiple fracture surfaces, extending from the lower edge of the wall stem plate to the ground.

#### (4) The effect of soil–wall interface element reduction coefficient

**Fig 7:**
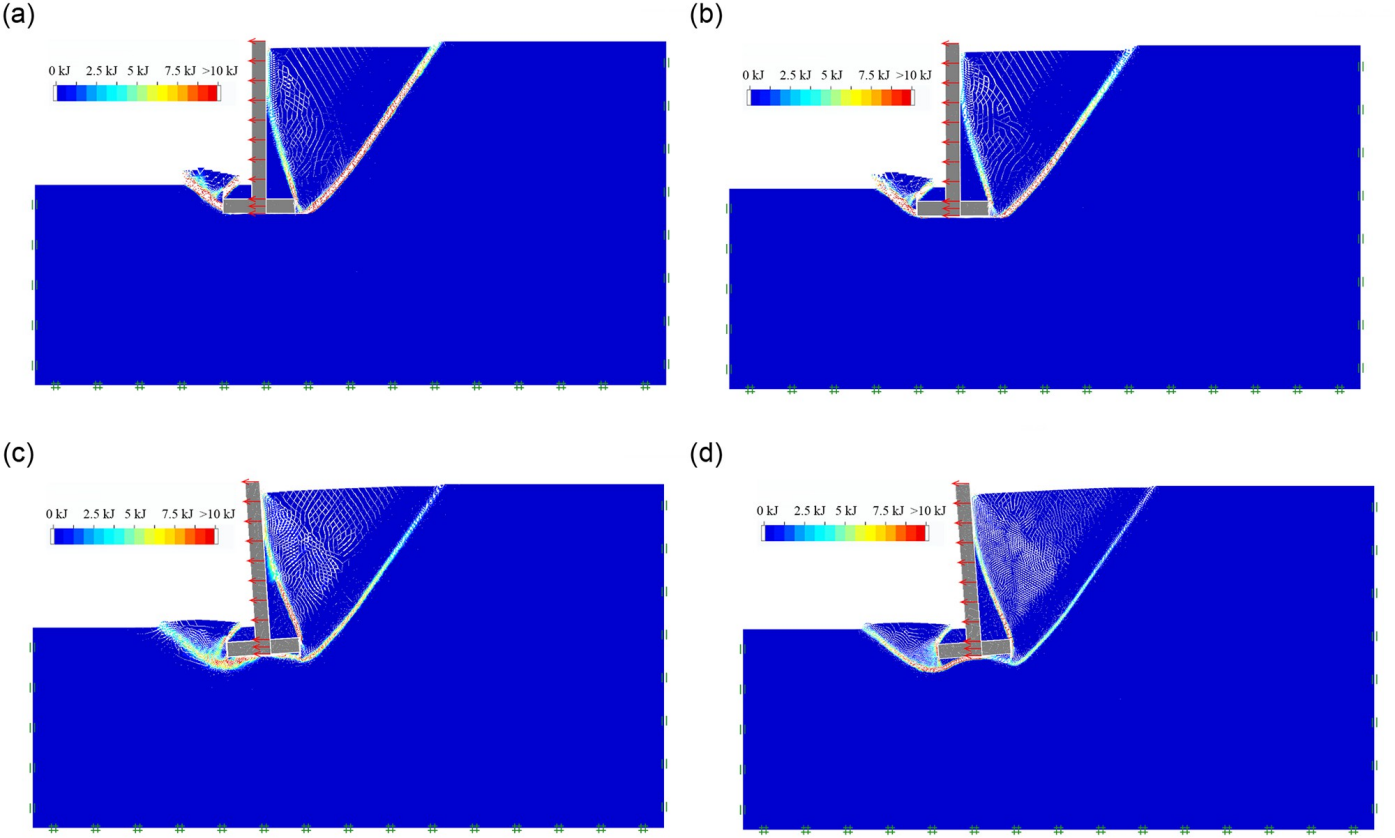
The soil mass final failure state of soil–wall interface element reduction coefficient. (a) K = 0; (b) K = 0.333; (c) K = 0.666; (d) K = 1.

As the soil mechanical parameters and geometric dimension of inverted T-type retaining walls are shown in Tables 1 and 2, For Group 4, when the soil–wall interface element reduction coefficient K is 0, 0.333, 0.666, and 1, respectively, the soil mass final failure state of retaining wall in active limit state is shown in Fig 7; the soil mass sliding surface, sliding process and final failure state is included in the S1 Appendix. It can be seen that as the soil–wall interface element reduction coefficient K increases from 0 to 1, that is, the internal friction angle of the soil-wall gradually increases to the soil internal friction angle; the inverted T-shaped retaining wall clearly shows the rule from translational sliding failure to overturning failure. The soil behind the inverted T-shaped retaining wall still develops the first and second failure surfaces behind the wall; when the soil–wall interface element reduction coefficient K is 0 and 0.333, the first failure surface extends from the lower edge of wall heel to the ground; when the soil–wall interface element reduction coefficient K is 0.666 and 1, the first failure surface extends from the center section below the wall bottom plate to the ground; when the soil–wall interface element reduction coefficient K increases from 0 to 1, the second failure surface always extends from the upper edge of the wall heel to the wall stem. A sliding failure surface in the soil in front of the wall changes from the state that directly develops from the lower edge of the wall toe to the ground to the state that extends from the bottom of wall stem to the ground.

#### (5) The effect of soil cohesion

**Fig 8:**
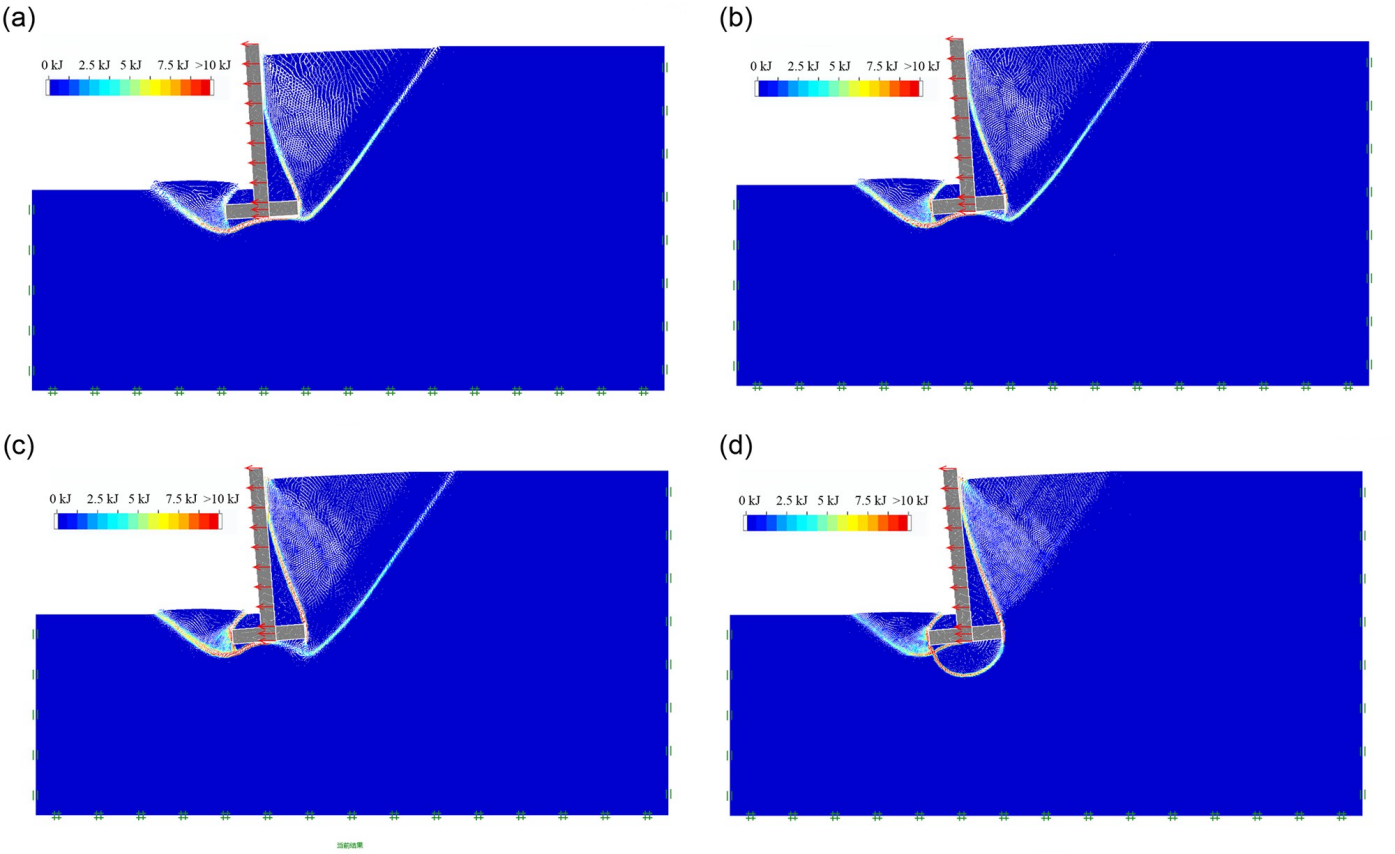
The soil mass final failure state of soil cohesion. (a) C = 5KPa; (b) C = 15KPa; (c) C = 25KPa; (d) C = 35KPa.

As the soil mechanical parameters and geometric dimension of inverted T-type retaining walls are shown in Tables 1 and 2, For Group 5, when the soil cohesion C is 5KPa, 15KPa, 25KPa and 35KPa, respectively, the soil mass final failure state of retaining wall in active limit state is shown in Fig 8; the soil mass sliding surface, sliding process and final failure state is included in the S1 Appendix. It is evident that as the soil cohesion C increases from 5KPa to 35KPa, the behavior of the retaining wall clearly indicates an overturning failure. The initial failure surfaces behind the wall evolve from developing near the lower edge of the wall heel to extending from the upper edge of the wall bottom plate to the ground, showing a gradual disappearance characteristic. In contrast, the second failure surface behind the wall continues to exist. The soil beneath the bottom plate forms an arc-shaped failure surface, originating from the lower edge of the wall toe and extending to the lower edge of the wall heel, ultimately reaching the second sliding surface. In front of the wall, the soil displays a distinct fracture surface that extends from the lower edge of the wall stem plate to the ground.

#### (6) The effect of soil internal friction angle

**Fig 9:**
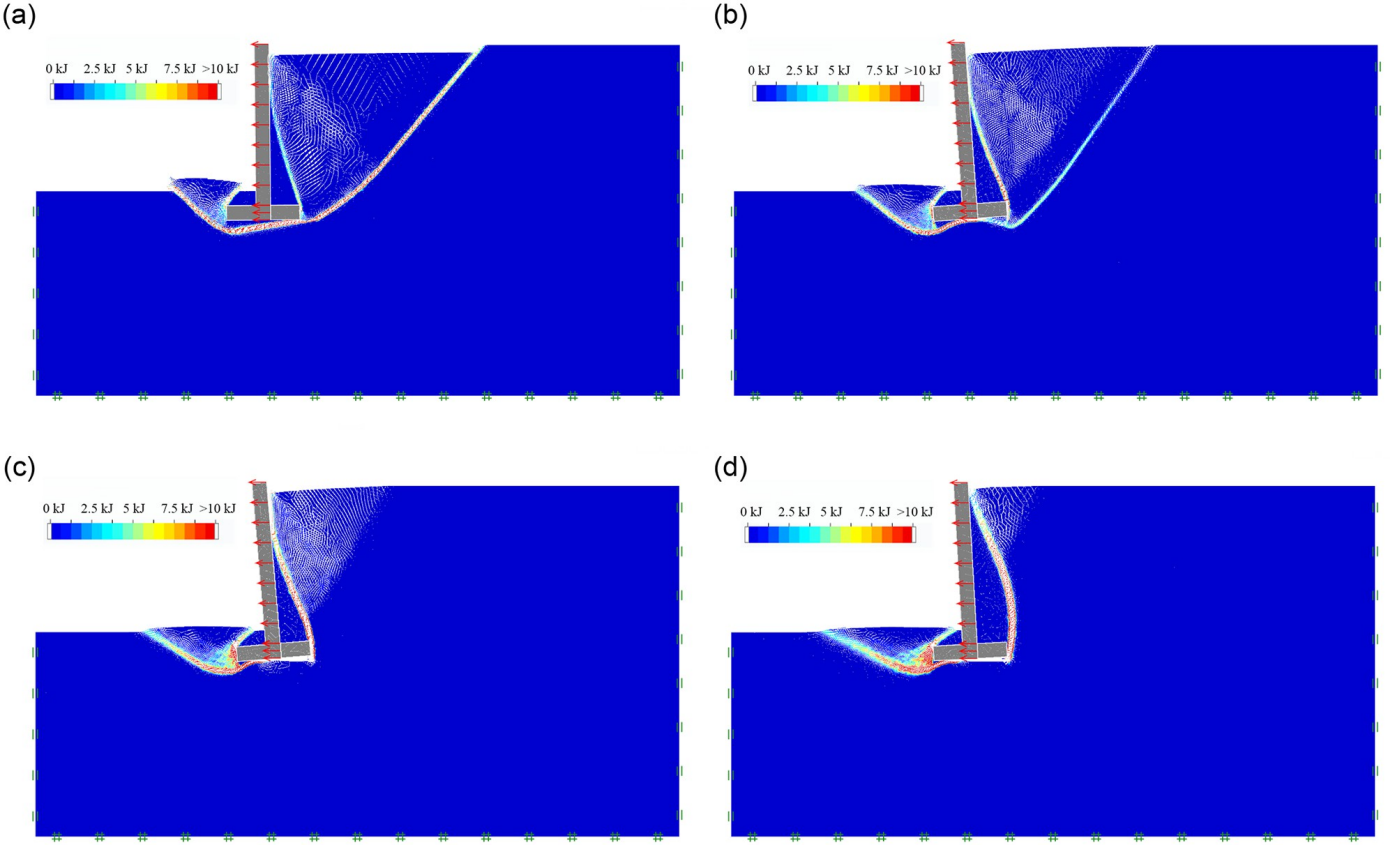
The soil mass final failure state of soil internal friction angle. (a) φ = 10°; (b) φ = 20°; (c) φ = 30°; (d) φ = 40°.

As the soil mechanical parameters and geometric dimension of inverted T-type retaining walls are shown in Tables 1 and 2, For Group 6, when the soil internal friction angle φ is 10°, 20°, 30° and 40°, respectively, the soil mass final failure state of retaining wall in active limit state is shown in Fig 9; the soil mass sliding surface, sliding process and final failure state is included in the S1 Appendix. It can be seen that as the soil internal friction angle φ increases from 10° to 40°, the retaining wall apparently shows the characteristic of overturning failure; Due to soil cohesion C is set as 15KPa, the soil strength increases gradually with the increase of soil internal friction angle φ. When the soil internal friction angle φ is 10° and 20°, the first failure surface extends from the lower edge of the wall heel to the ground; when the soil internal friction angle φ is 30° and 40°, the first failure surface gradually moves upwards in parallel, reducing the sliding failure area of the soil behind the wall; when the soil internal friction angle φ increases from 10° and 40°, the second failure surface always extends from the upper edge of the wall heel to the wall stem. A sliding failure surface in the soil in front of the wall changes from the state that directly develops from the lower edge of the wall heel to the ground to the state that extending from the bottom of wall toe to the ground.

As the wall heel width and the wall toe width increase from 1m to 4m, the soil behind the inverted T-shaped retaining wall still develops the first and second failure surfaces behind the wall. When the wall heel width and the wall toe width vary from 1m to 4m, the inverted T-type retaining wall has the change characteristic from overturning failure to sliding failure. As the bottom plate thickness increases from 0.5m to 1.25m, the retaining wall shows the characteristic of overturning failure. As the soil–wall interface element reduction coefficient K increases from 0 to 1, the soil-wall’s internal friction angle gradually increases to the soil internal friction angle; the inverted T-shaped retaining wall clearly shows the rule from translational sliding failure to overturning failure. It is evident that as the soil cohesion C increases from 5KPa to 35KPa, the behaviour of the retaining wall clearly indicates an overturning failure. It can be seen that as the soil internal friction angle φ increases from 10° to 40°, the retaining wall apparently shows the characteristic of overturning failure.

### 3.2. The earth pressure distribution law of inverted T-type retaining wall in active limit state

**Fig 10:**
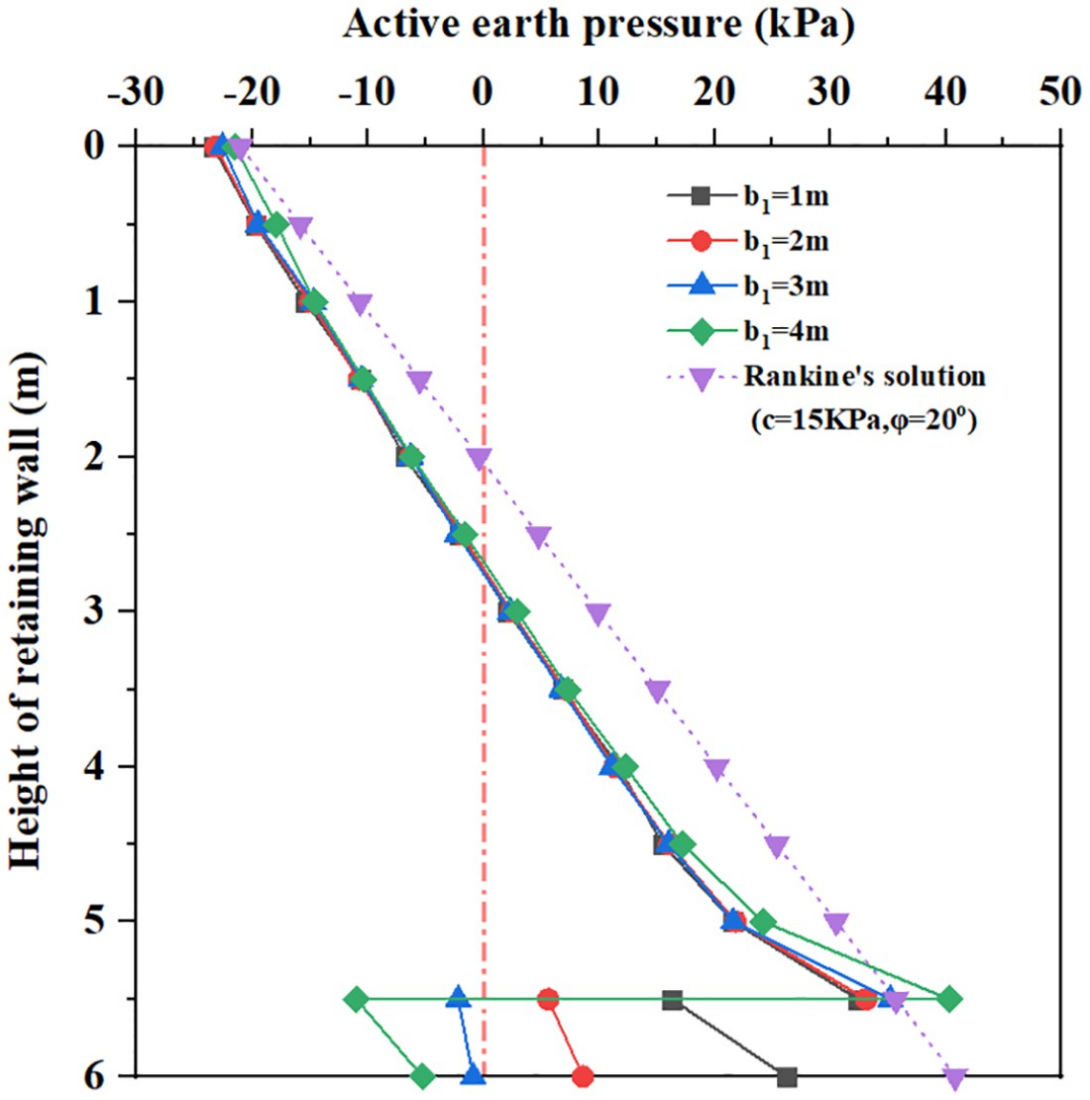
The earth pressure distribution with different wall heel width.

**Fig 11:**
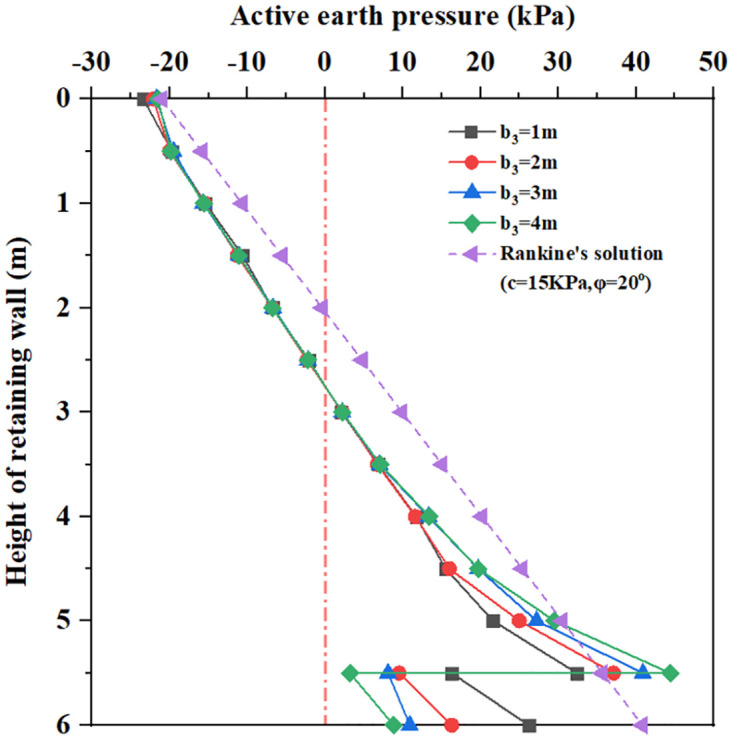
The earth pressure distribution with different wall toe width.

**Fig 12:**
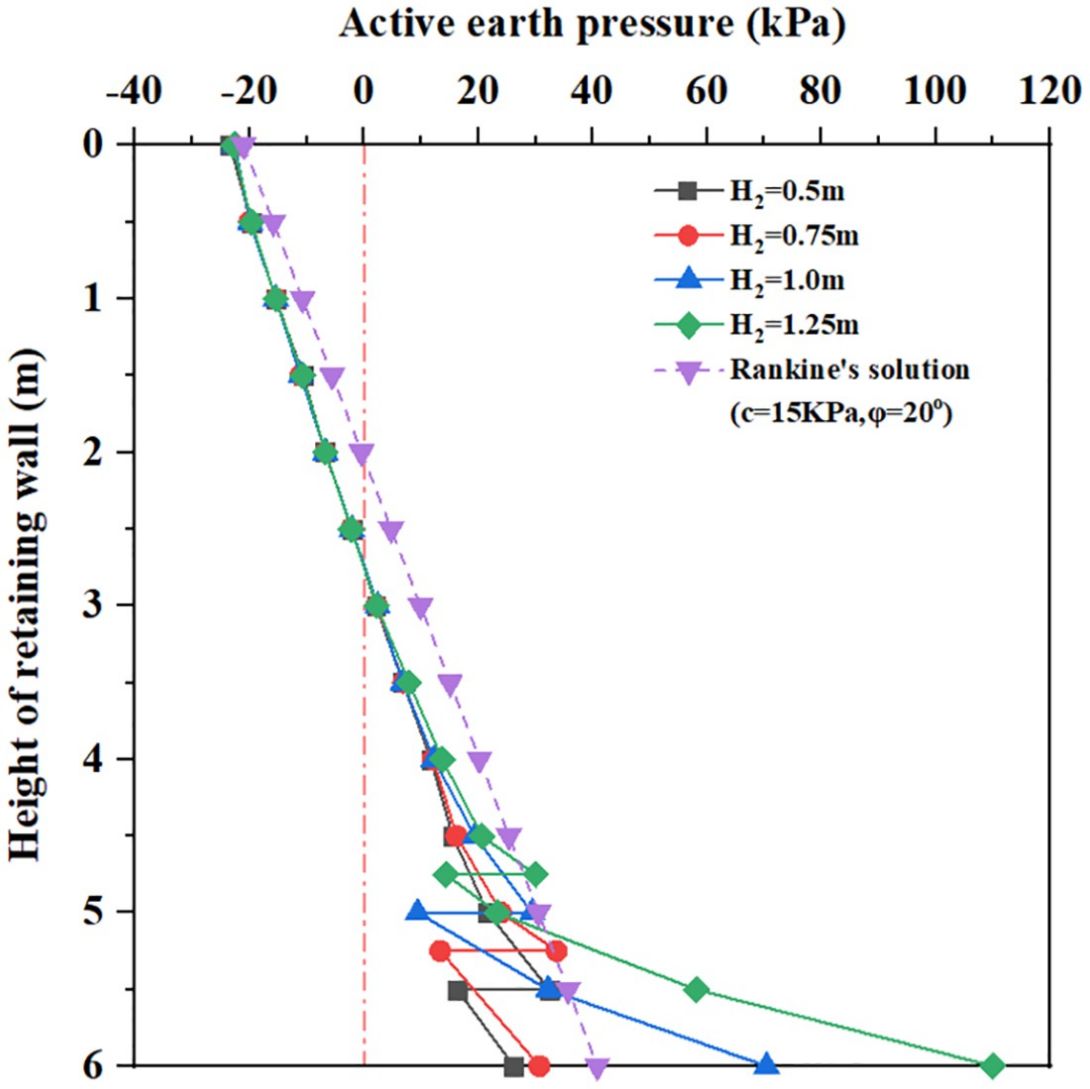
The earth pressure distribution with different bottom plate thickness.

**Fig 13:**
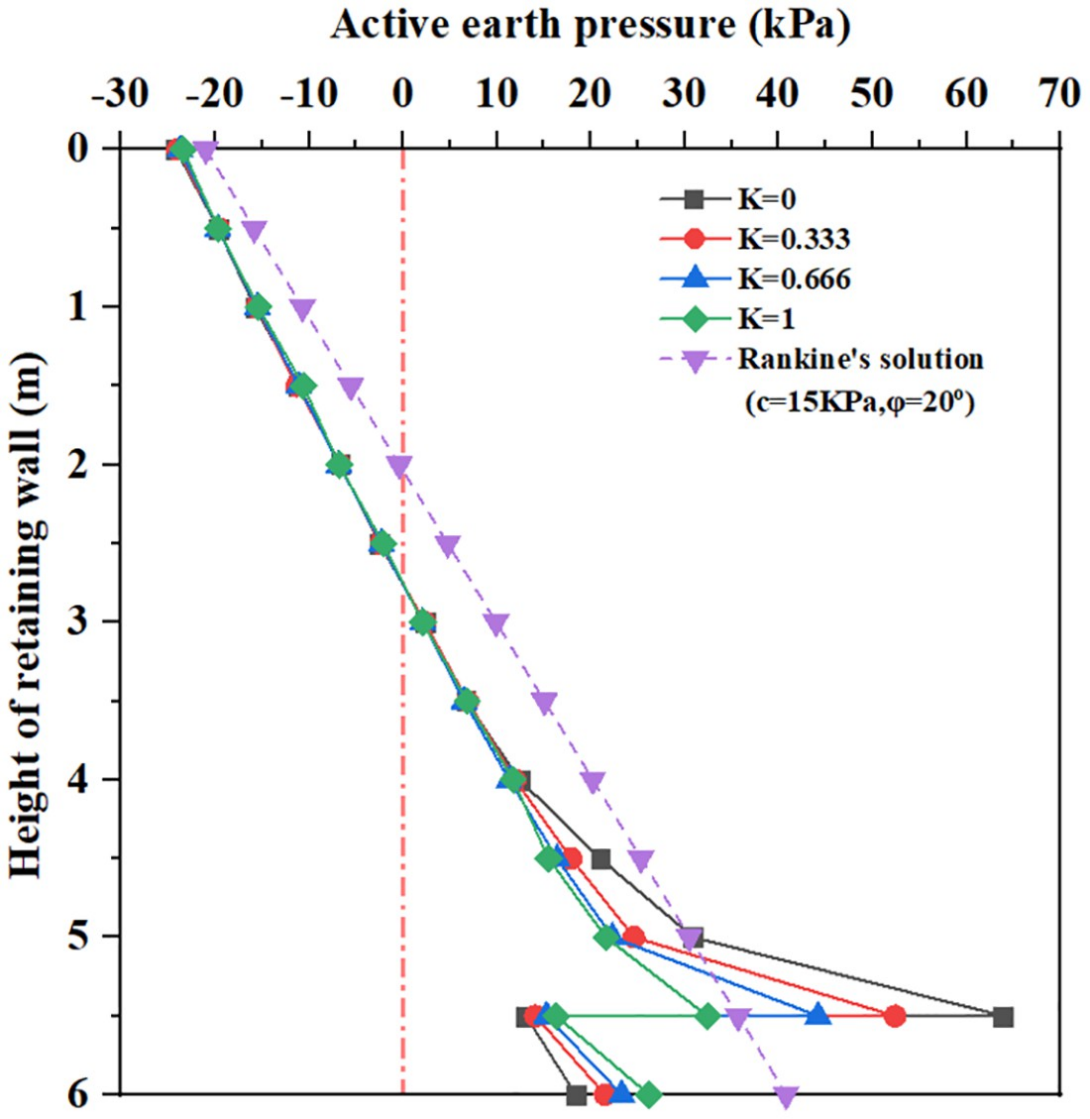
The earth pressure distribution with different soil–wall interface element reduction coefficient.

**Fig 14:**
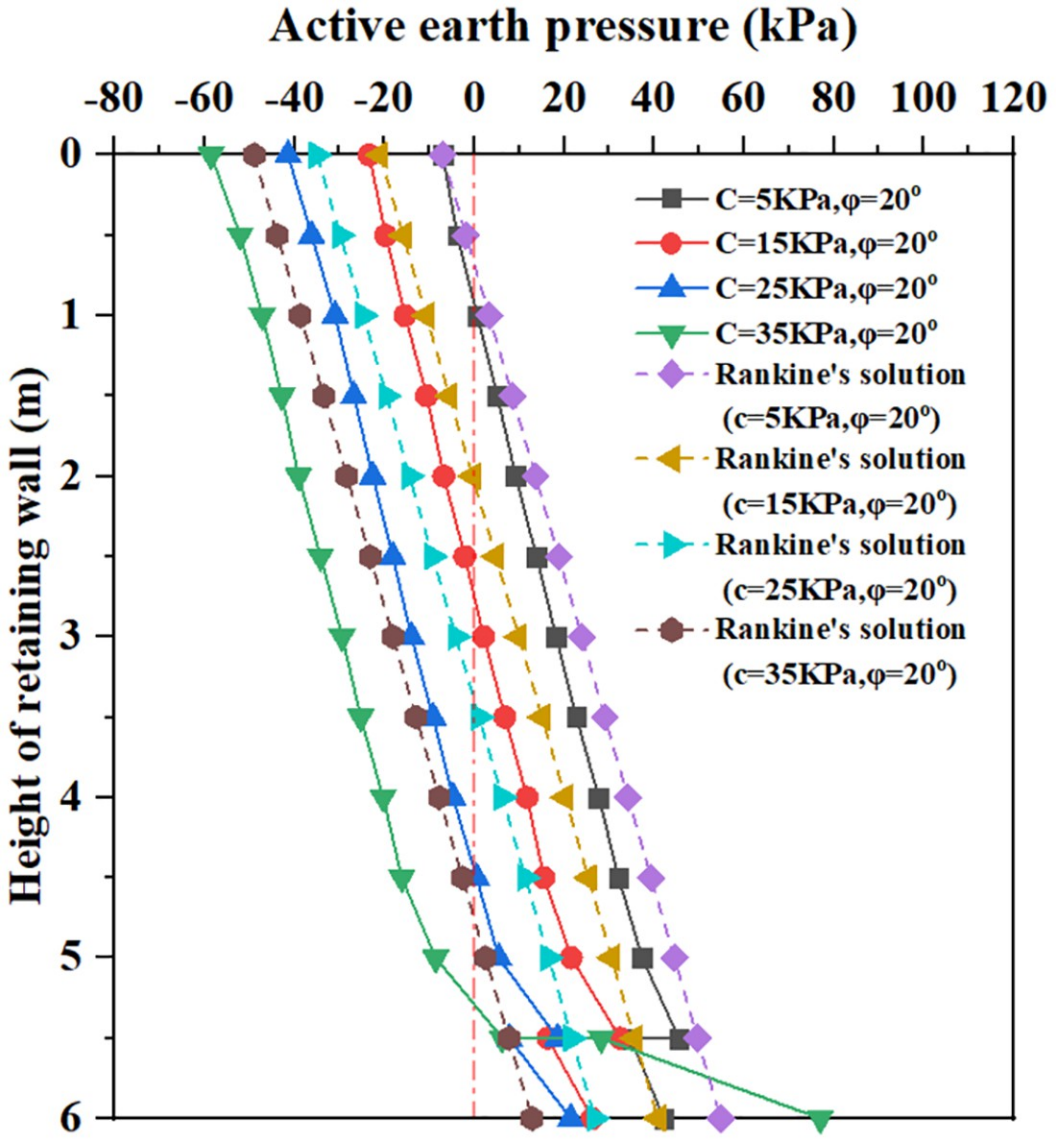
The earth pressure distribution with different soil cohesion.

**Fig 15:**
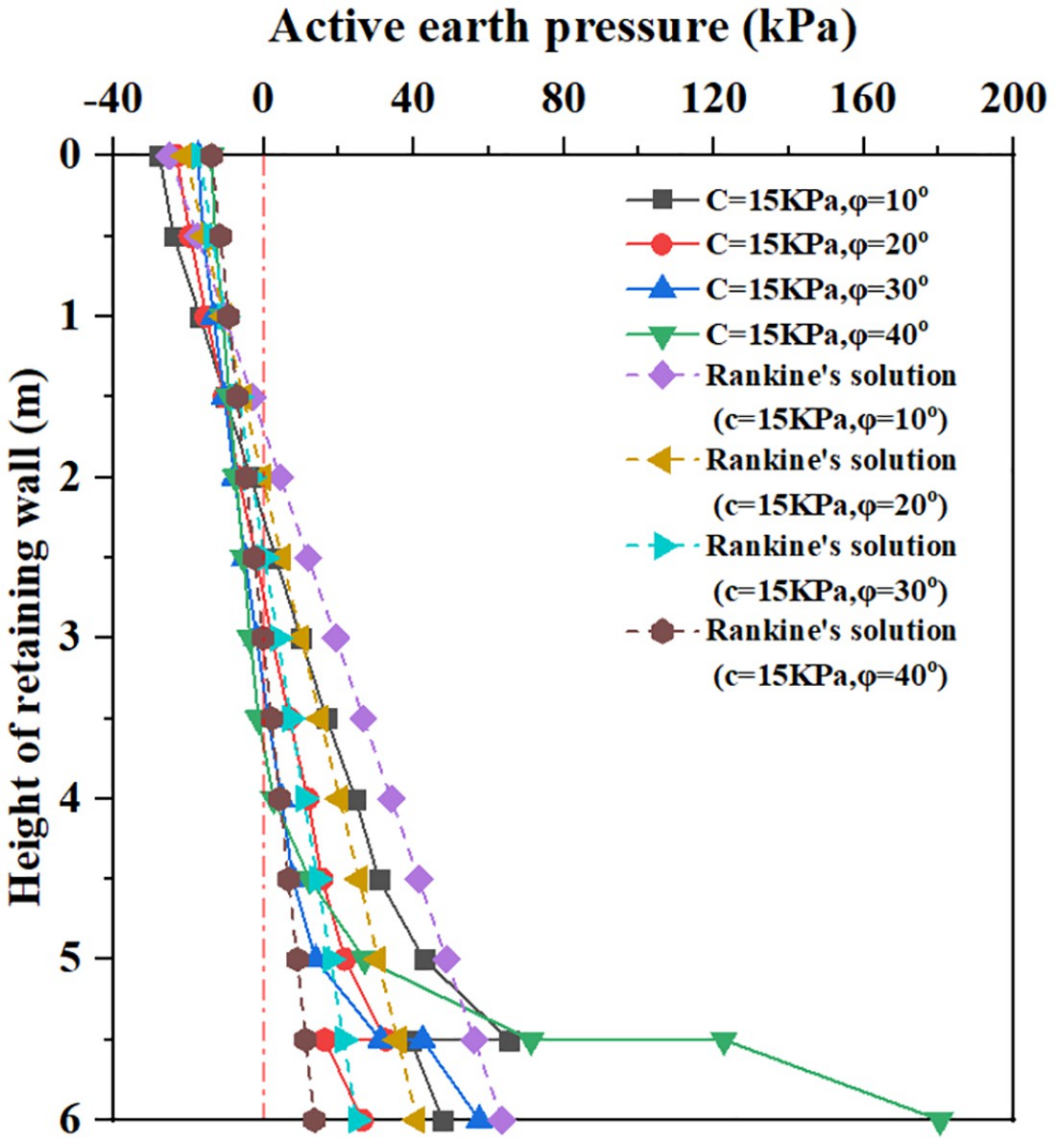
The earth pressure distribution with different soil internal friction angle.

The earth pressure distributions of inverted T-type retaining wall in active limit state with influencing factors are shown in Figs 10–15. The distribution law of earth pressure is described as follows, in which Coulomb’s earth pressure theory [35] is chosen as a comparative analysis:

Figs 10 and 11 show that for wall heel width b_1_ = 1m to 4m and wall toe width b_3_ = 1m to 4m, within 3.5m below the top of the retaining wall, the earth pressure curve is very close to and smaller than the Coulomb earth pressure. As the depth increases, there is a large difference between the earth pressure curves. When the buried depth is from 0 to 5.5m, the earth pressure increases with the increase of buried depth; at the same time, the larger the wall heel width and wall toe width, the greater the earth pressure. When the buried depth is 5.5m, all earth pressure curves show a rapidly decreasing change. When the buried depth is 5.5 to 6.0m, the earth pressure increases with the increase of wall heel width and wall toe width, but the value of earth pressure is smaller than that of the Coulomb earth pressure curve at 5.5m. It can be seen that increasing wall heel width and wall toe width have little influence on lateral earth pressure distribution.

Fig 12 shows that for bottom plate thickness H_2_ = 0.5m to 1.25m, the earth pressure curves are very close and smaller than the Coulomb earth pressure within 3.5m below the top of the retaining wall. As the depth increases, there is a significant difference in the earth pressure curves; when the burial depth is 0 to the top surface of the bottom plate, the earth pressure increases with the increase of the buried depth; at the same time, the larger the bottom plate thickness, the greater the earth pressure. At the burial depth of the top surface of the bottom plate, the earth pressure has a rapidly decreasing change; when the burial depth is between the top surface of the bottom plate and 6.0m, the earth pressure increases with the increase of bottom plate thickness. For the bottom plate thickness H_2_ = 0.5 to 0.75m, the value of the earth pressure is smaller than the value of the Coulomb earth pressure curve at 5.5m. For bottom plate thickness H_2_ = 1m to 1.25m, the value of earth pressure increases sharply with burial depth and ultimately exceeds the Coulomb earth pressure curve at 5.5m. It can be seen that increasing bottom plate thickness will cause a significant increase in lateral earth pressure within the range of bottom plate thickness.

Fig 13 shows that for soil–wall interface element reduction coefficient K = 0 to 1, within 3.5m below the top of the retaining wall, the earth pressure curve is very close to and smaller than the Coulomb earth pressure. As the depth increases, there is a large difference between the earth pressure curves. When the buried depth is from 0 to 5.5m, the earth pressure increases with the increase of buried depth; at the same time, the larger the soil–wall interface element reduction coefficient, the greater the earth pressure. When the buried depth is 5.5m, all earth pressure curves show a rapidly decreasing change. When the buried depth is 5.5 to 6.0m, the earth pressure increases with the increase of soil–wall interface element reduction coefficient, but the value of earth pressure is smaller than that of the Coulomb earth pressure curve at 5.5m. It can be seen that increasing soil–wall interface element reduction coefficient has little influence on lateral earth pressure distribution.

Fig 14 shows that for soil cohesion C = 5Kpa to 35Kpa and soil internal friction angle φ = 20°, within 5.5m below the top of the retaining wall, the earth pressure curve increases with the increase of buried depth. The analysis of all curves shows that at the same depth, the active earth pressure of soil decreases with the rise of soil cohesion, in which the critical depth of earth pressure sharply increases with the rise of soil cohesion. When the buried depth is 5.5m, all earth pressure curves show a rapidly decreasing change. When the buried depth is 5.5m to 6.0m, the earth pressure increases with the increase of buried depth. For the soil cohesion C = 5 Kpa to 25Kpa, the value of the earth pressure is smaller than the value of the Coulomb earth pressure curve at 6.0m. For soil cohesion C = 35Kpa, the value of earth pressure increases sharply with burial depth and ultimately exceeds the Coulomb earth pressure curve at 6.0m. It can be seen that increasing soil cohesion reduces the total earth pressure acting on the soil, which is more conducive to the stability of retaining walls.

Fig 15 shows that for soil internal friction angle φ = 10° to 40° and soil cohesion C = 15Kpa, within 5.5m below the top of the retaining wall, the earth pressure curve increases with the increase of buried depth, the critical depth of earth pressure gradually increases with the increase of soil internal friction angle. For the soil internal friction angle φ = 10° to 20°, When the buried depth is 5.5m, the earth pressure curves show a rapidly decreasing change. When the buried depth is 5.5 to 6.0m, the earth pressure increases with the increase of buried depth, and the value of the earth pressure is smaller than the value of the earth pressure curve at 5.5m. For the soil internal friction angle φ = 30° to 40°, When the buried depth is 5.5m, the earth pressure curves show a rapidly increasing change. When the buried depth is 5.5 to 6.0m, the earth pressure increases with the increase of buried depth; the value of the earth pressure of φ = 40° is larger than the value of the Coulomb earth pressure curve at 6.0m. It can be seen that increasing soil internal friction angle results in a significant increase in lateral soil pressure within the range of bottom plate thickness.

### 3.3. The stability safety factor of inverted T-type retaining wall in active limit state

**Fig 16:**
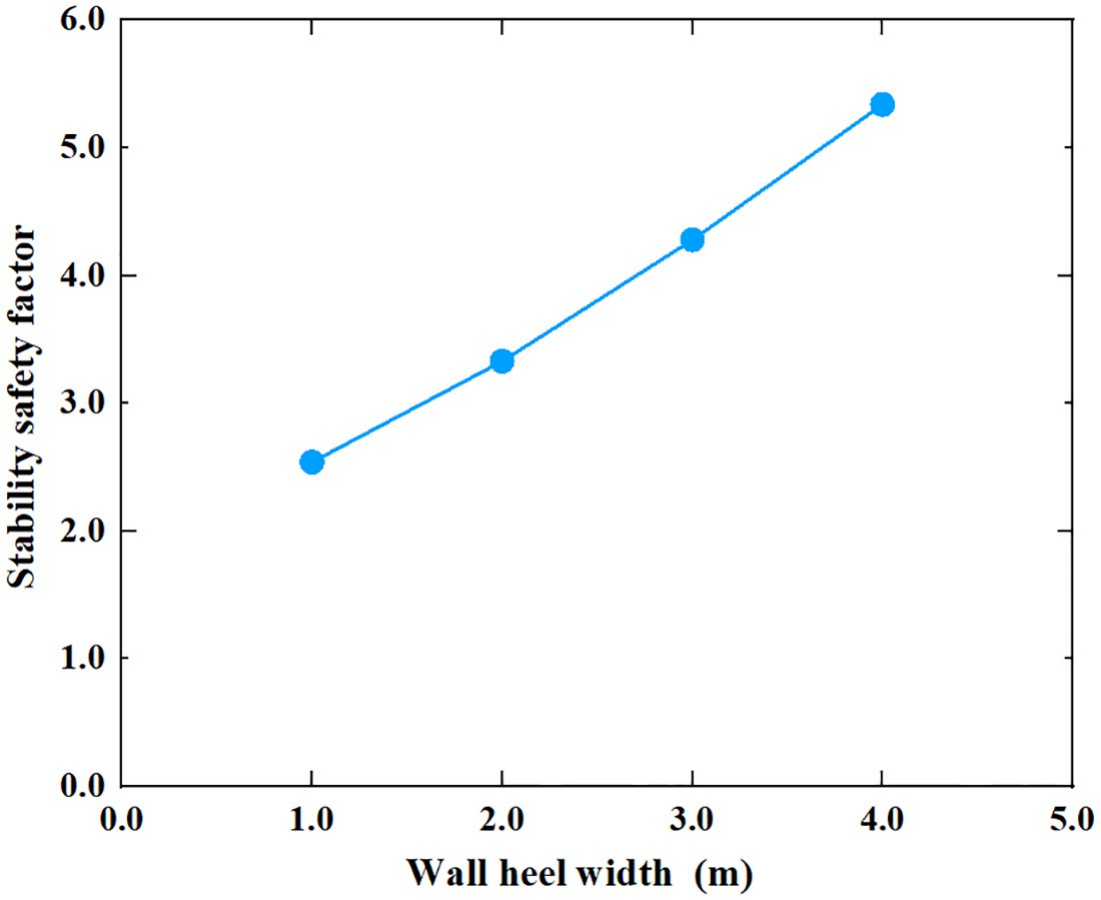
The relationship between the stability safety factor and wall heel width.

**Fig 17:**
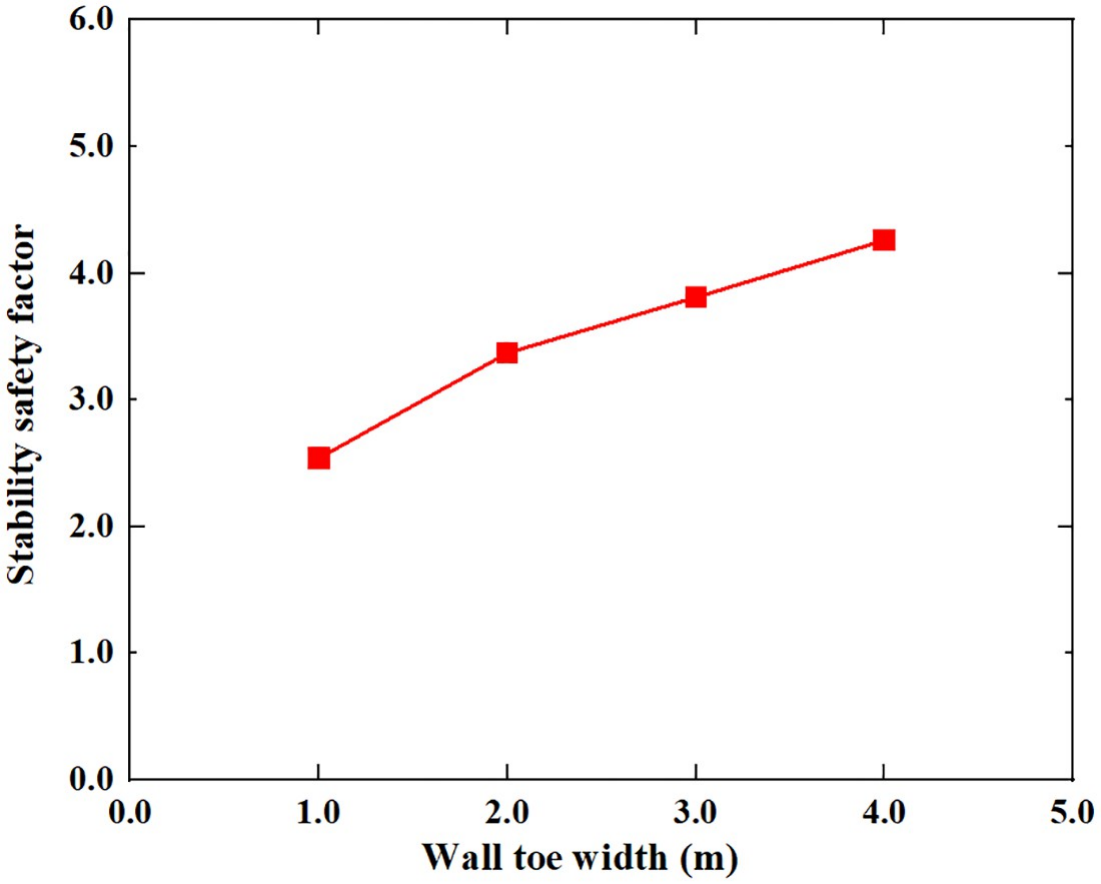
The relationship between the stability safety factor and wall toe width.

**Fig 18:**
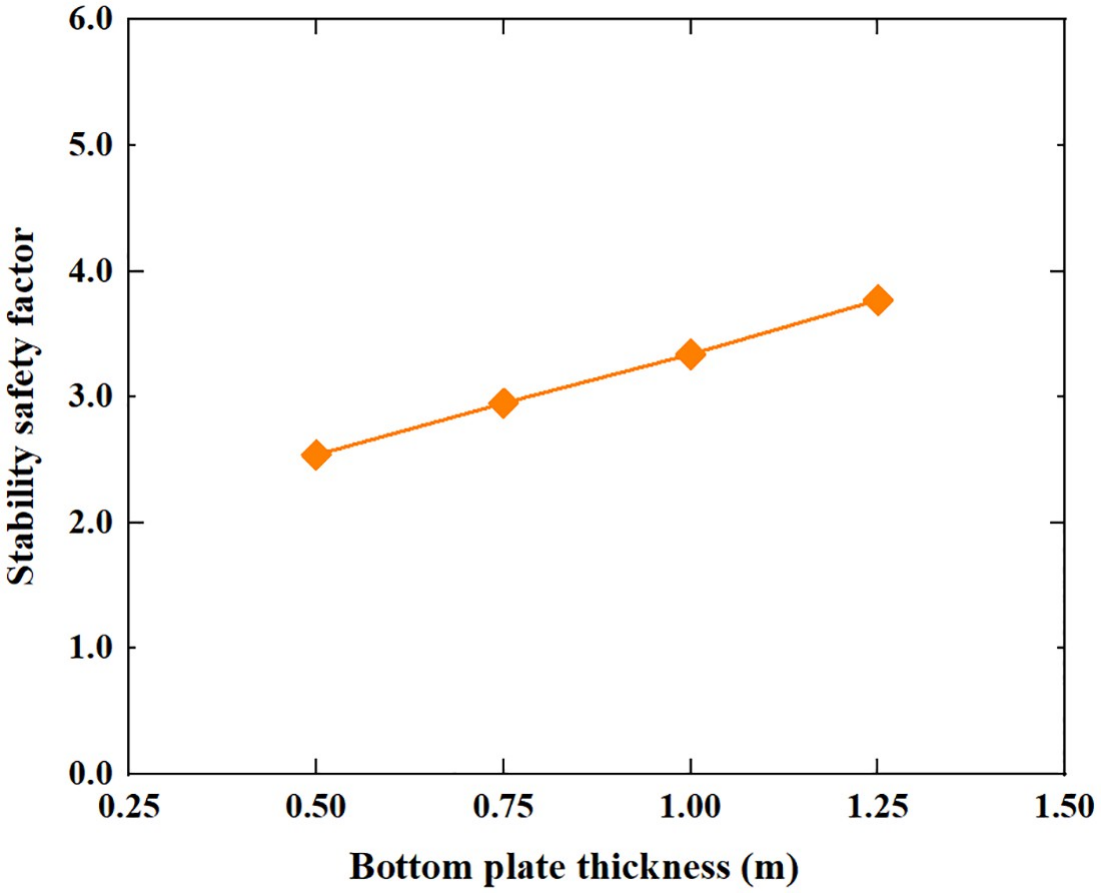
The relationship between the stability safety factor and bottom plate thickness.

**Fig 19:**
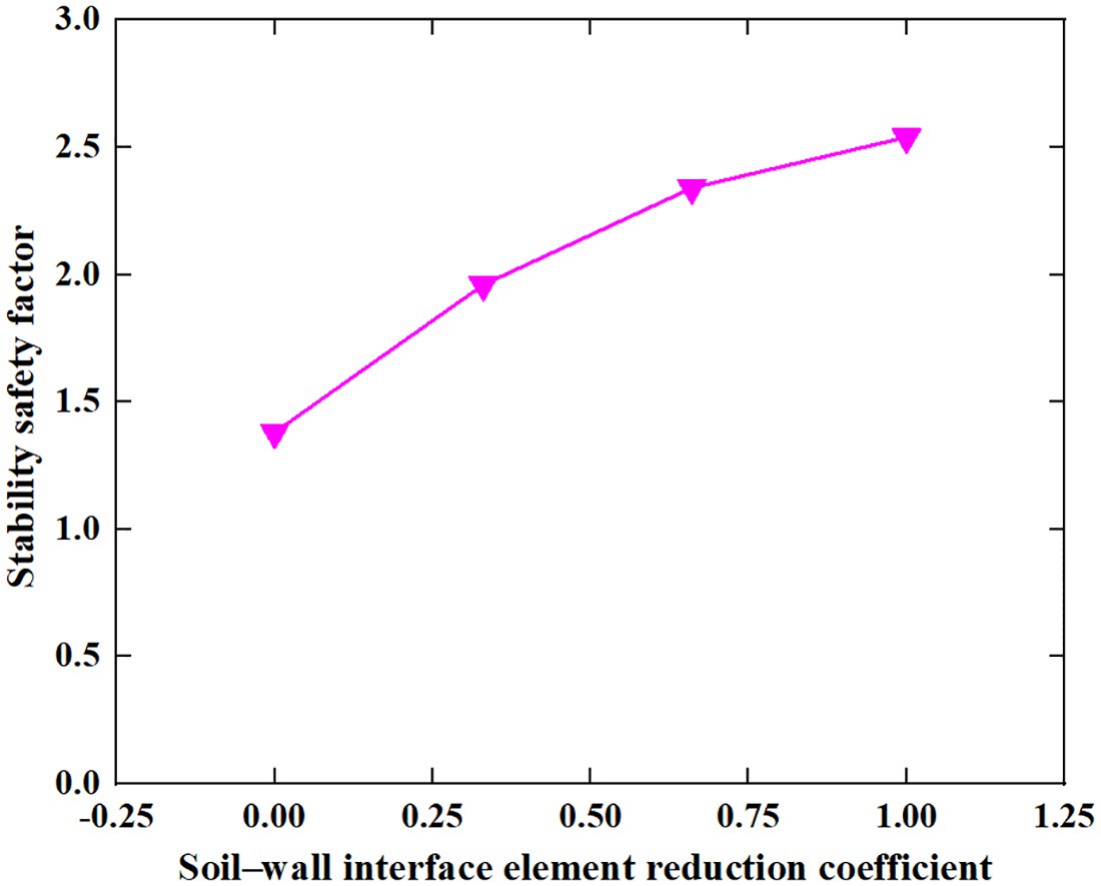
The relationship between the stability safety factor and soil–wall interface element reduction coefficient.

**Fig 20:**
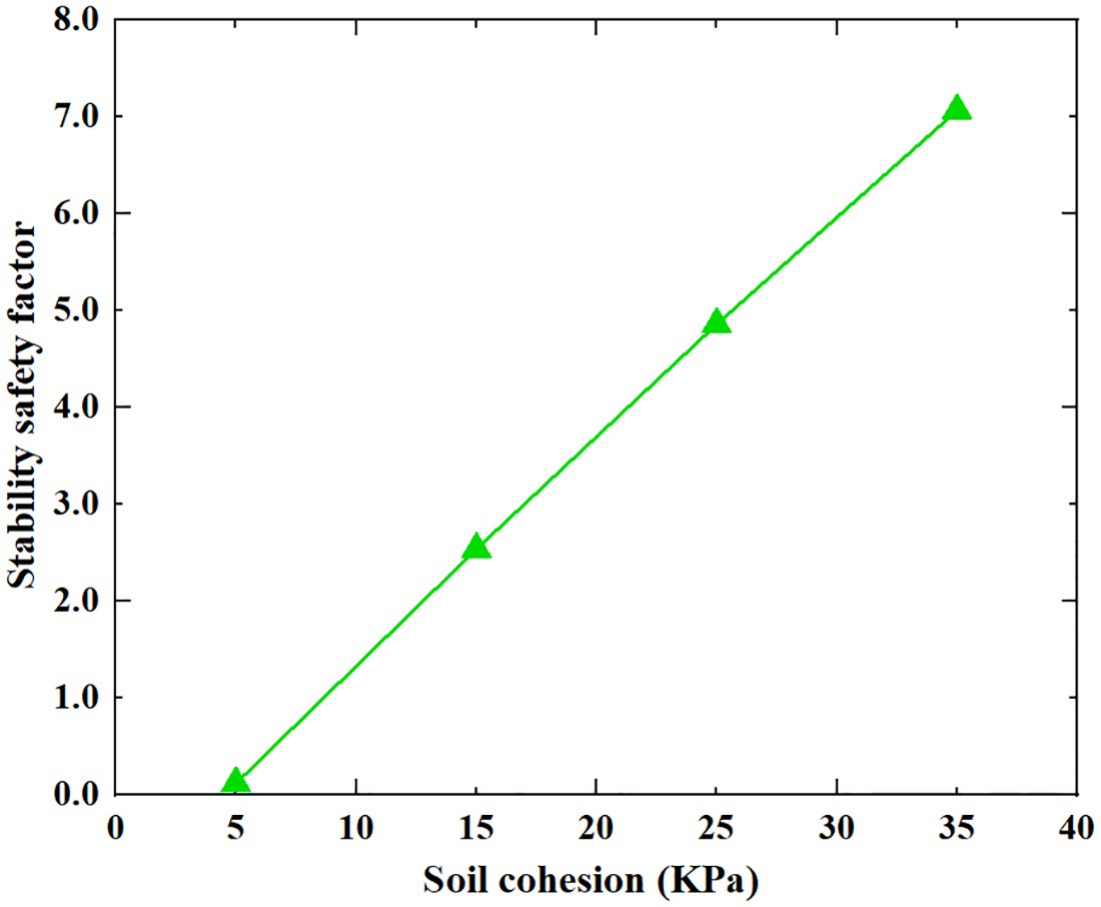
The relationship between the stability safety factor and soil cohesion.

**Fig 21:**
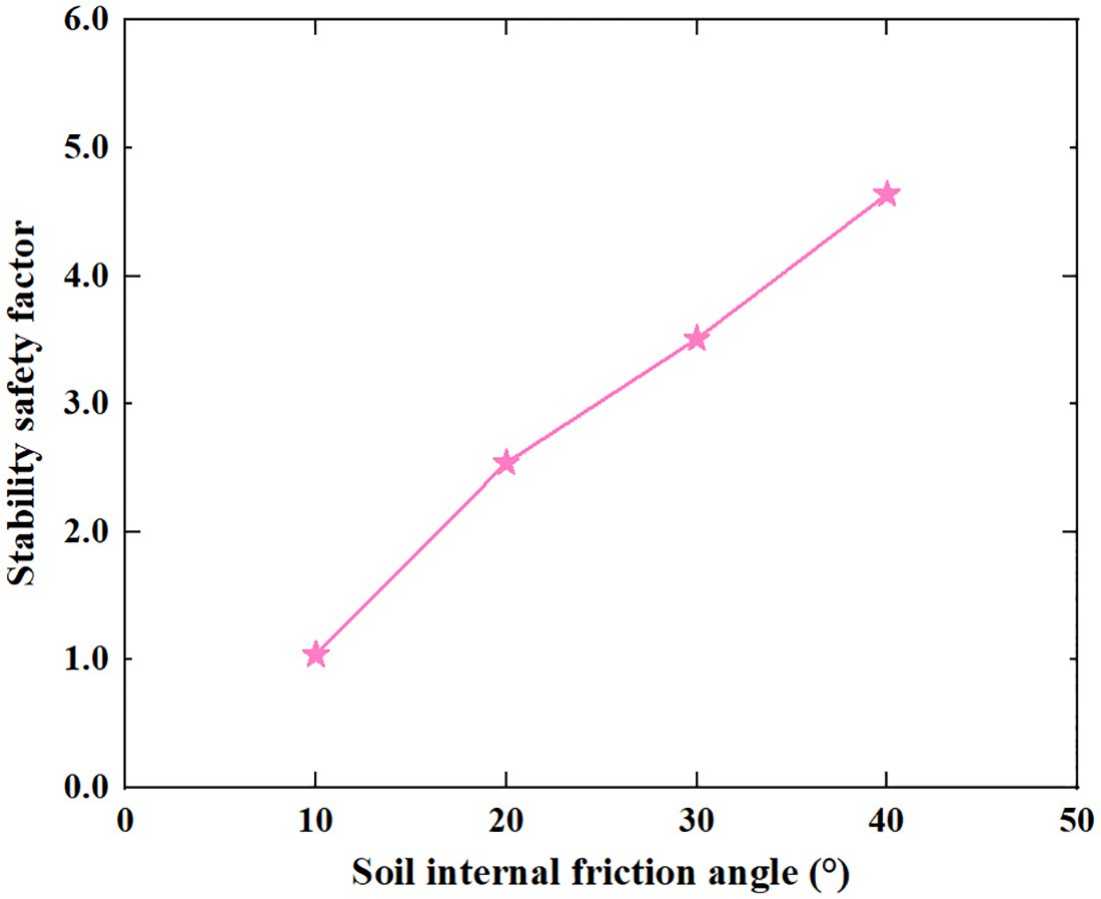
The relationship between the stability safety factor and soil internal friction angle.

Based on the Limit Analysis of Multiplier load, the stability safety factor of the inverted T-type retaining wall varies with the width of the wall heel plate, width of wall toe plate, thickness of bottom plate, soil–wall interface friction angle, soil cohesion and soil internal friction angle of filling are shown in Figs 16–21. The stability safety factor of retaining wall is described as follows:

As can be seen from Figs 16 and 17, the stability safety factor of the inverted T-type retaining wall showed a gradually increasing trend as the width of the wall heel plate and wall toe plate increased. When the width of the wall heel plate and wall toe plate increased from 1m to 4m, the stability safety factor of the retaining wall rose from 2.54 to 5.24 and from 2.54 to 4.26, respectively. On average, the safety factor of retaining wall increases by 0.45 for every 0.5m increase in the width of the wall heel plate; the safety factor of the retaining wall increases by 0.29 when the width of the toe plate increases by 0.5m. It can be seen that for the same width, increasing the width of the wall heel plate can improve the stability of the retaining wall more than increasing the width of wall toe plate. In the process of retaining wall construction, priority should be given to increasing the width of wall heel plate. The larger the width of the wall heel plate and the width of the wall toe plate, the greater the construction difficulty, in actual engineering, the appropriate width of the wall heel plate and the wall toe plate should be selected according to the specific needs of the foundation stress, eccentricity, construction conditions and stability.

Fig 18 shows that as the bottom plate thickness increases, the stability safety factor of the inverted T-type retaining wall gradually increases. When the bottom plate thickness is increased from 0.5m to 1.25m, the stability safety factor of the retaining wall is increased from 2.54 to 3.77, on average, for every 0.5m increase in the width of wall plate thickness, the safety factor of the retaining wall is increased by 0.62. Increasing the bottom plate thickness can significantly improve the stability safety factor of the retaining wall.

As shown in Fig 19, as the soil-wall interface element reduction coefficient increases, that is, the soil-wall internal friction angle gradually increases to the soil internal friction angle, the stability safety factor of the retaining wall gradually increases. When the soil-wall interface element reduction coefficient increases from 0 to 1, the stability safety factor of the retaining wall increases from 1.38 to 2.54, on average, for every 0.25 increase in soil-wall interface element reduction coefficient, the safety factor of the retaining wall increases by 0.29. To increase the stability of retaining walls, the measures can be taken to increase the soil wall internal friction angle at the wall-soil junction.

The filling material for retaining walls is generally taken from the construction site. For example, retaining walls near mountains can be filled with excavated rocks, crushed stones, etc.; in some areas, the filling soil may be cohesive, sandy, etc. The properties of fill soil vary greatly, and it is necessary to analyze the impact of different soil parameters. As shown in Figs 20 and 21, as the soil cohesion and soil internal friction angle increase, the stability safety factor of retaining wall both gradually increase. When soil cohesion increased from 5KPa to 35KPa, the stability safety factor of retaining wall rose from 0.12 to 7.07, on average, for every increase of 5KPa in soil cohesion, the safety factor of the retaining wall increased by 1.16. When the soil internal friction angle increases from 10° to 40°, the stability safety factor of the retaining wall increases from 1.04 to 4.64; on average, for every 5° increases in soil internal friction angle, the safety factor of retaining wall increases by 0.6. Increasing soil cohesion and soil internal friction angle in engineering reinforcement treatment can effectively improve the safety of the retaining wall.

## 4. Conclusions

This study aimed to investigate the effects of width of wall heel plate, width of wall toe plate, thickness of bottom plate, soil–wall interface friction angle, soil cohesion and soil internal friction angle of filling on the failure characteristics of sliding surface, the earth pressure distribution law and stability safety factor of retaining walls. The main conclusions of the study are as follows.

1. As the wall heel width and the wall toe width increase, the soil behind the inverted T-shaped retaining wall still develops the first and second failure surfaces behind the wall. When the wall heel width and the wall toe width increase, the inverted T-type retaining wall has the change characteristic from overturning failure to sliding failure. As the bottom plate thickness increases, the retaining wall shows the characteristic of overturning failure. As the soil–wall interface element reduction coefficient increases, the inverted T-shaped retaining wall clearly shows the rule from translational sliding failure to overturning failure. As the soil cohesion increases, the behaviour of the retaining wall clearly indicates an overturning failure. As the soil internal friction angle increases, the retaining wall apparently shows the characteristic of overturning failure.
2. The safety factor of retaining wall increases by 0.45 for every 0.5m increase in the width of the wall heel plate; the safety factor of the retaining wall increases by 0.29 when the width of the wall toe plate increases by 0.5m; for every 0.5m increase in the width of wall plate thickness, the safety factor of the retaining wall is increased by 0.62; for every 0.25 increase in soil-wall interface element reduction coefficient, the safety factor of the retaining wall increases by 0.29; for every increase of 5KPa in soil cohesion, the safety factor of the retaining wall increased by 1.16; for every 5° increases in soil internal friction angle, the safety factor of retaining wall increases by 0.6.
3. It can be seen that the stability of the retaining wall is improved less by increasing the width of the wall toe plate than by increasing the width of the wall heel plate. Increasing the bottom plate thickness can significantly improve the stability safety factor of the retaining wall. To increase the stability of retaining walls, the measures can be taken to increase the soil wall internal friction angle at the wall-soil junction. Increasing soil cohesion and soil internal friction angle in engineering reinforcement treatment can effectively improve the safety of the retaining wall. The study has important practical significance for the design of inverted T-type retaining walls.

## Supporting information

S1 Appendix (DOCX)
