# Supplementary material for: Study on the failure characteristics of sliding surface and stability analysis of inverted t-type retaining wall in active limit state
Source: PLoS One. 2024 Feb 8;19(2):e0298337. doi: 10.1371/journal.pone.0298337 (PMC10852246; doi:10.1371/journal.pone.0298337)
Supplement: S1 Appendix — (DOCX) [file pone.0298337.s001.docx]

**S1 Appendix**

**(1) The effect of wall heel width on the soil mass sliding surface, sliding process and final failure state**


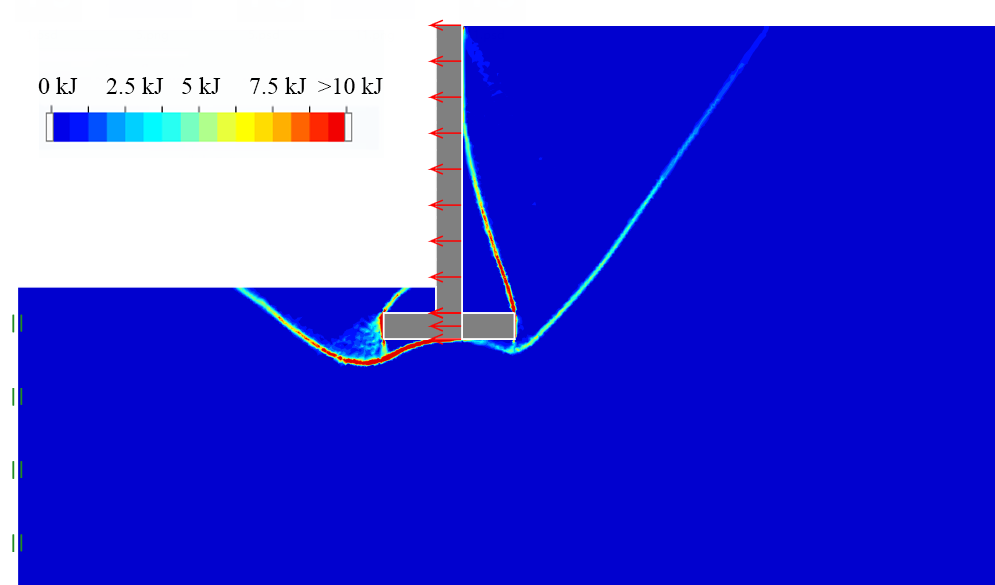


(a) Soil mass sliding surface


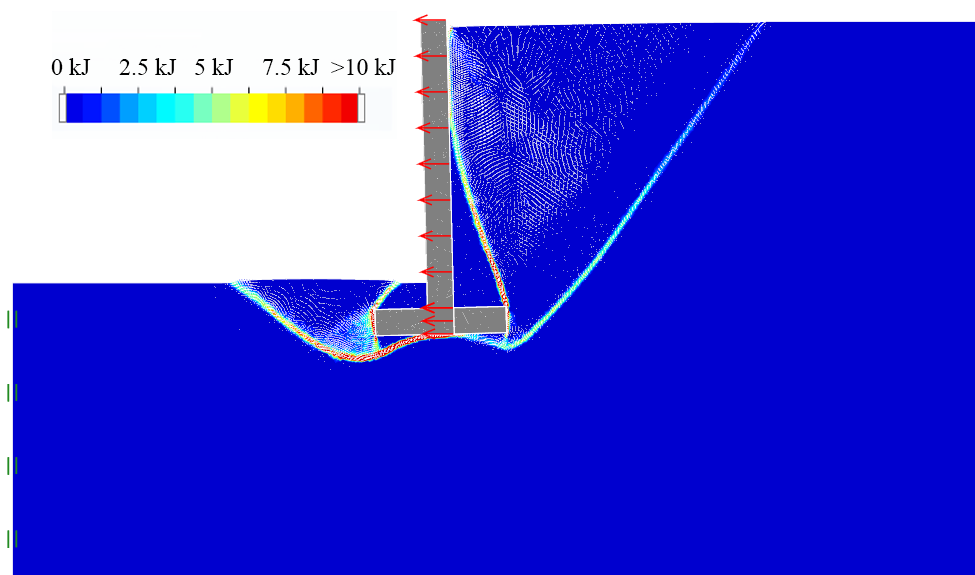


(b) Sliding process


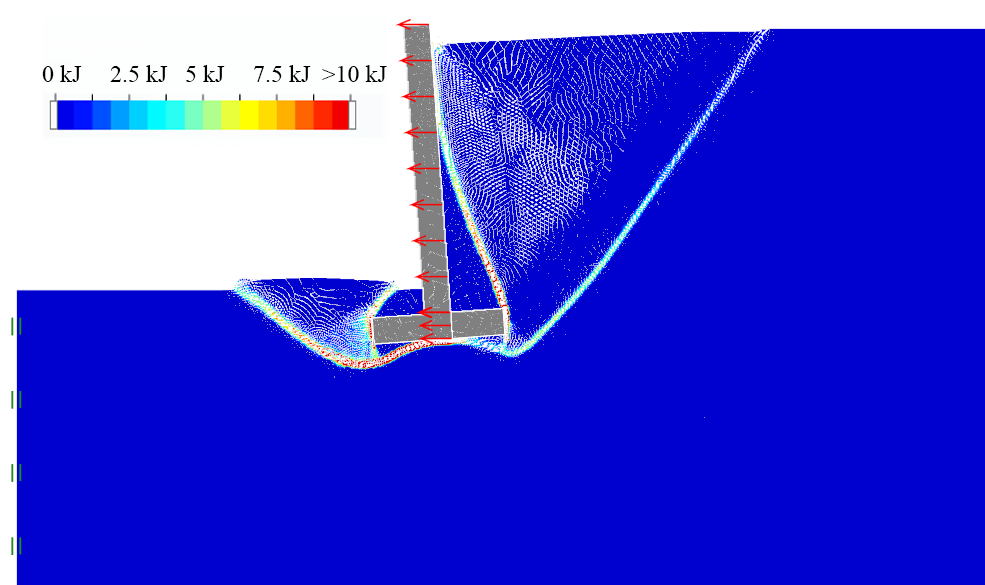


(c) Final failure state

**The soil mass sliding surface, sliding process and final failure state of wall heel width b_1_=1m.**


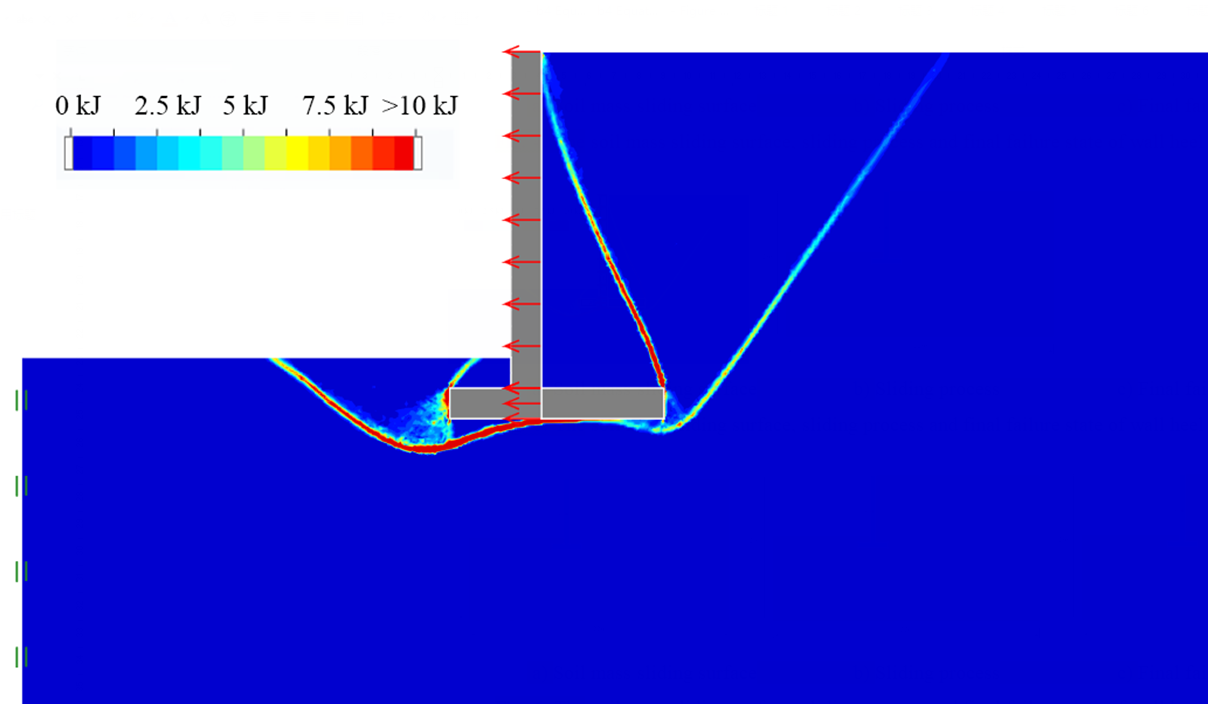


(a) Soil mass sliding surface


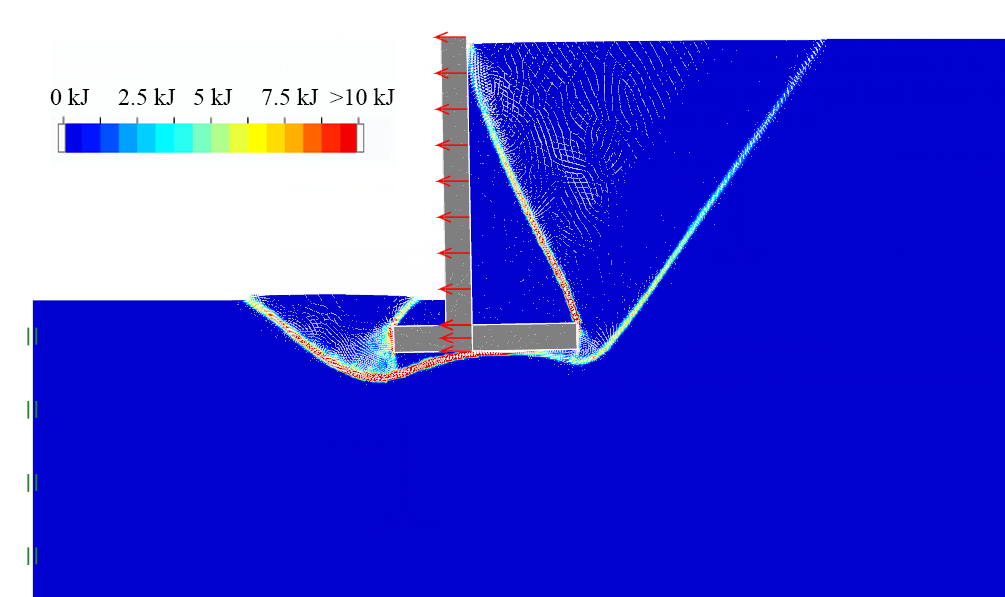


(b) Sliding process


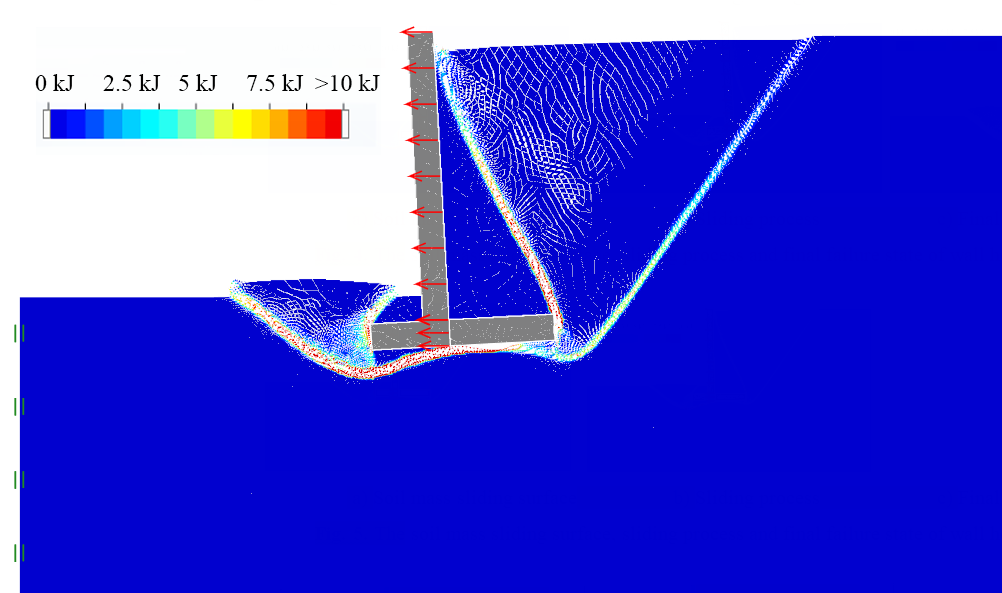


(c) Final failure state

**The soil mass sliding surface, sliding process and final failure state of wall heel width b_1_=2m.**


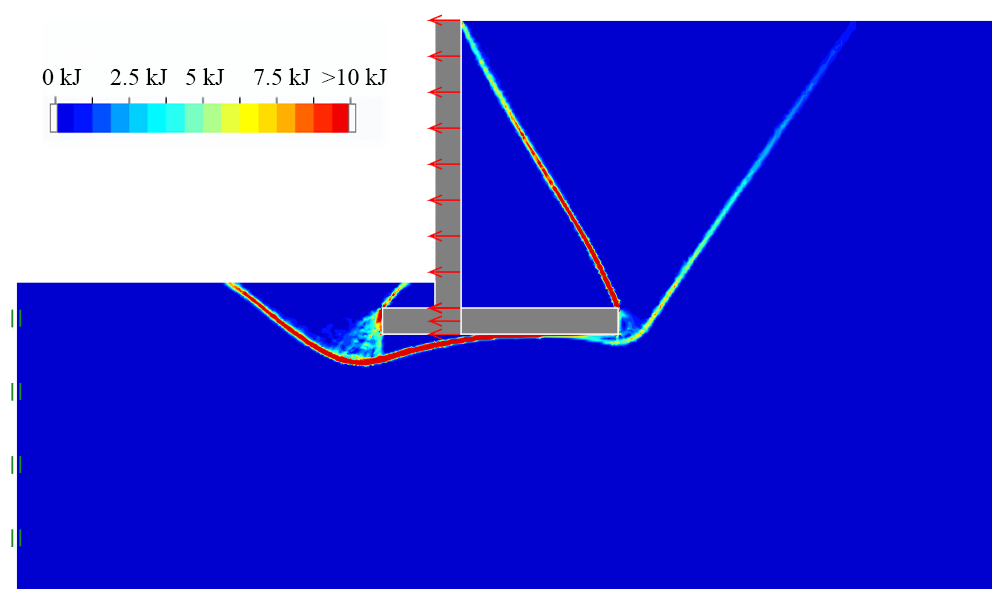


(a) Soil mass sliding surface


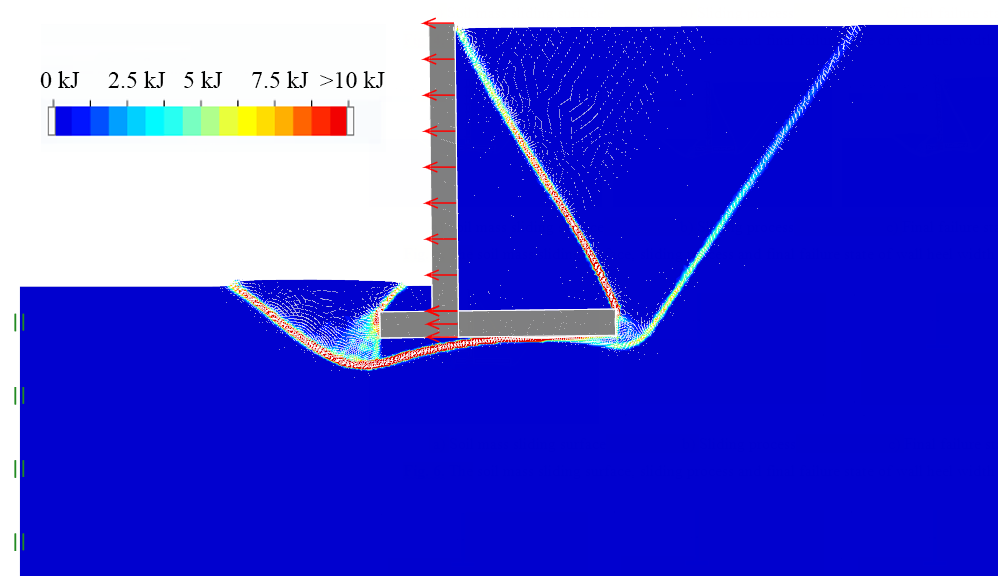


(b) Sliding process


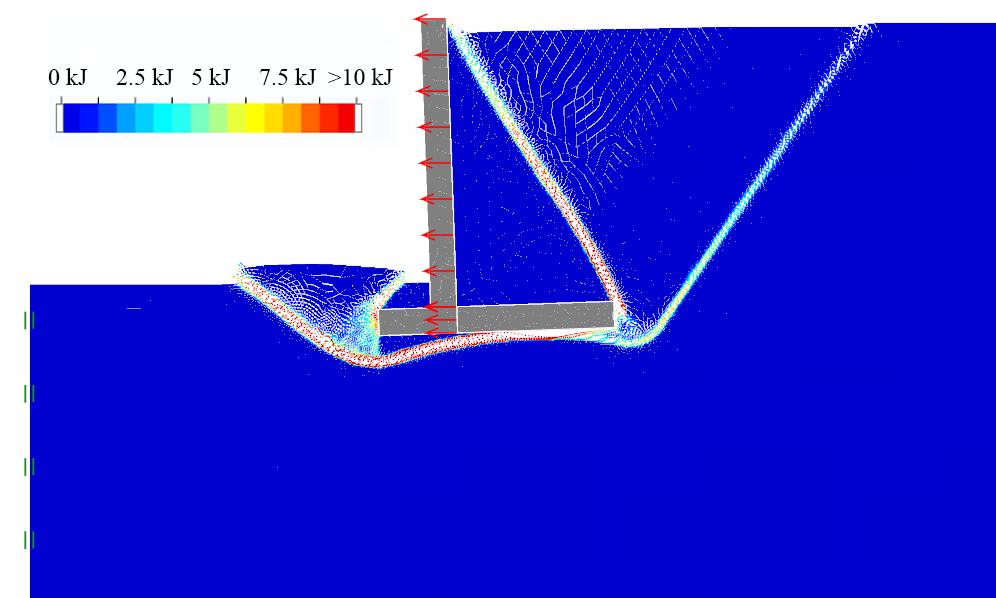


(c) Final failure state

**The soil mass sliding surface, sliding process and final failure state of wall heel width b_1_=3m.**


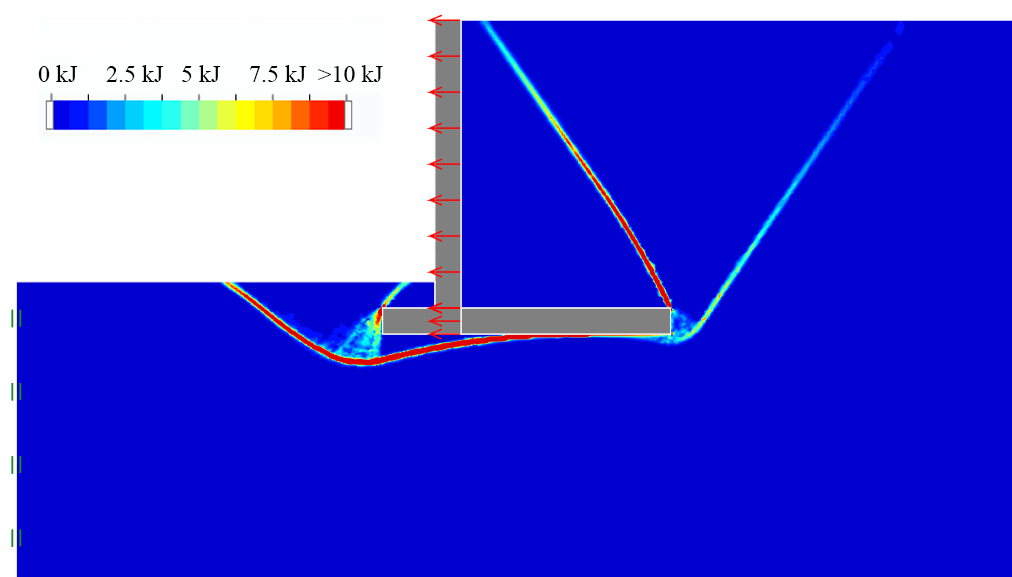


(a) Soil mass sliding surface


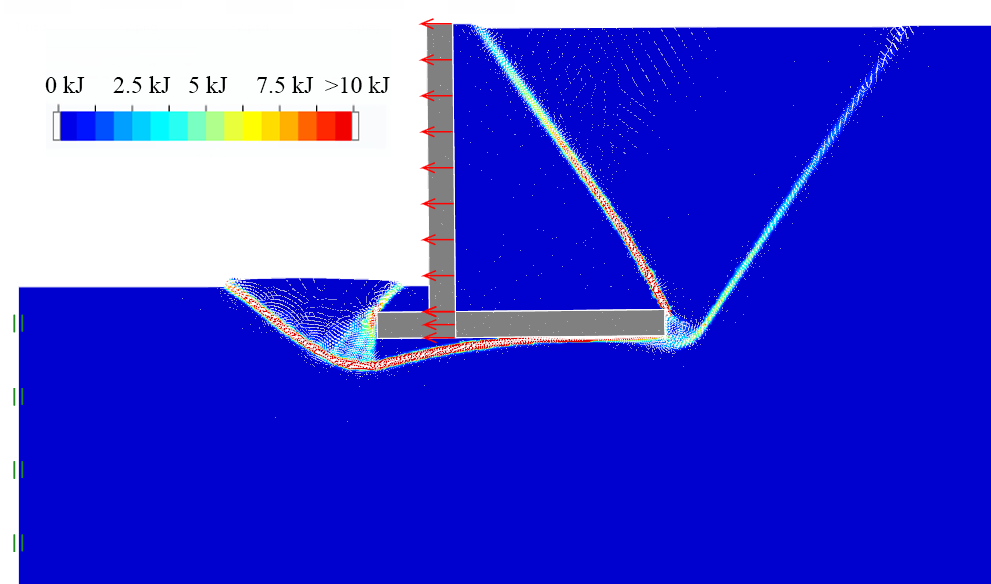


(b) Sliding process


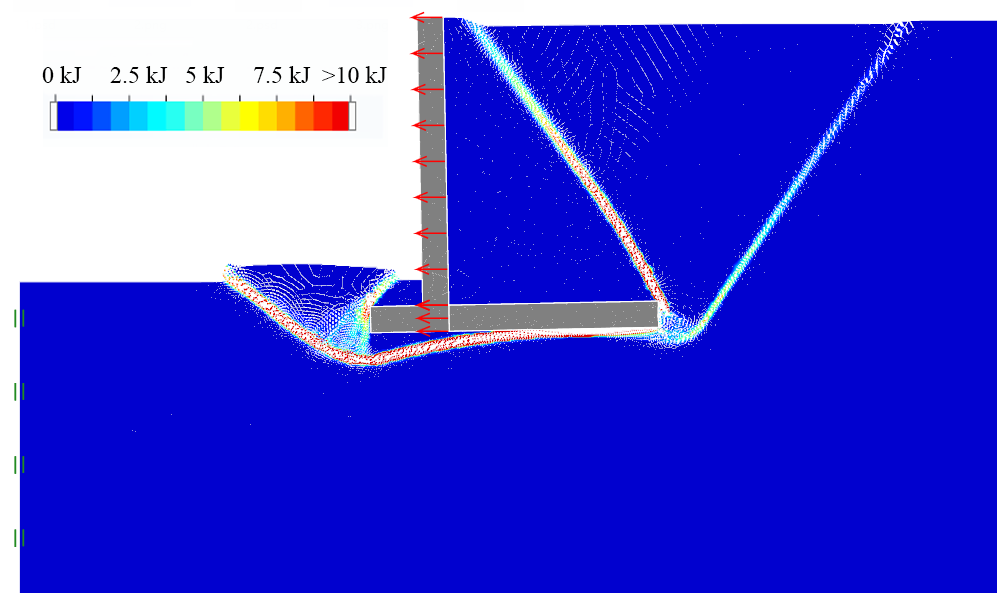


(c) Final failure state

**The soil mass sliding surface, sliding process and final failure state of wall heel width b_1_=4m.**

**(2) The effect of wall toe width on the soil mass sliding surface, sliding process and final failure state**


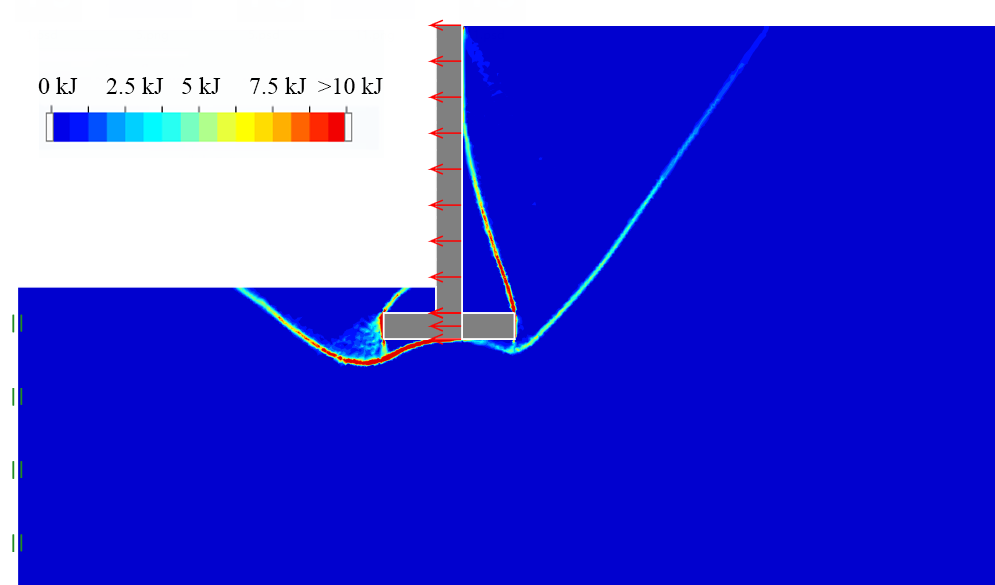


(a) Soil mass sliding surface


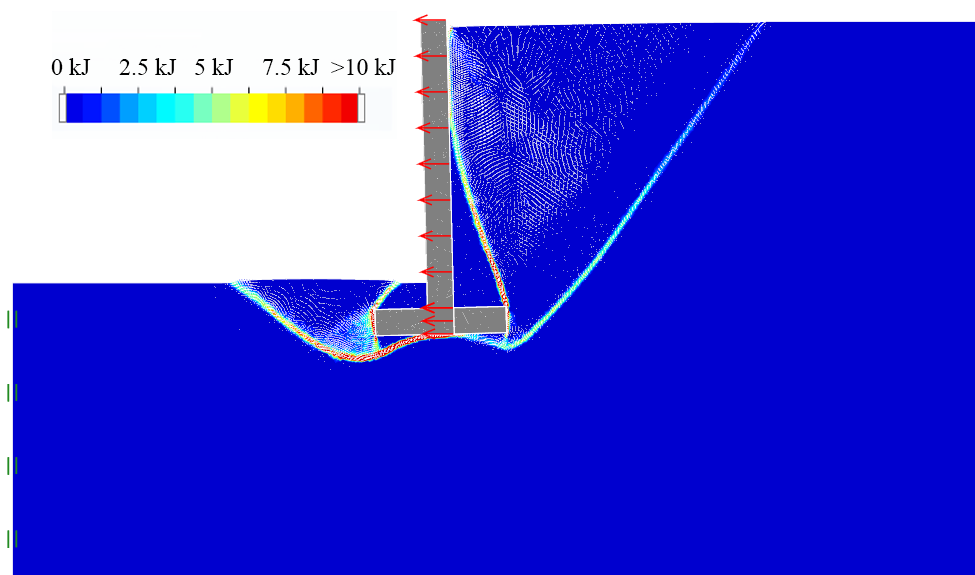


(b) Sliding process


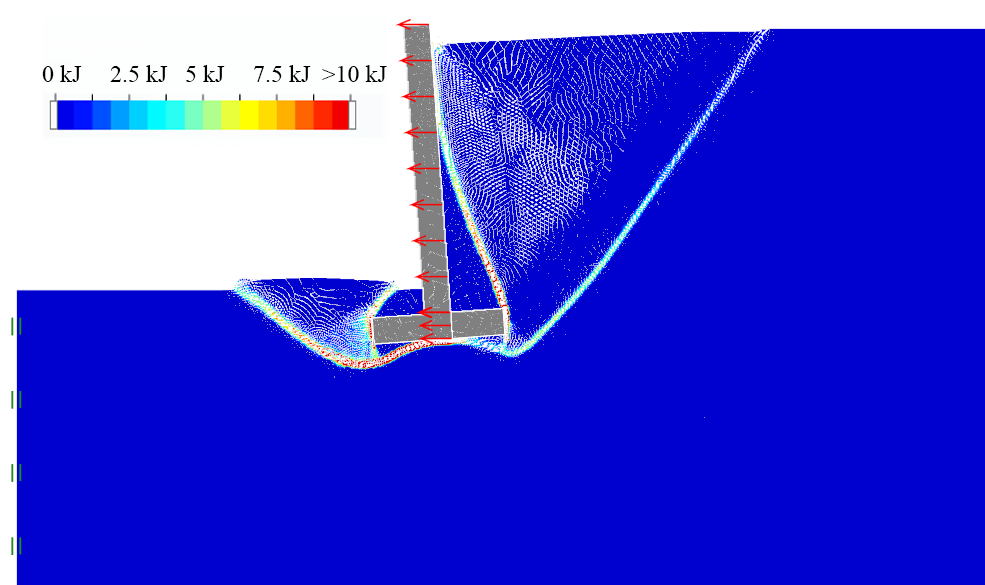


(c) Final failure state

**The soil mass sliding surface, sliding process and final failure state of wall toe width b_3_=1m.**


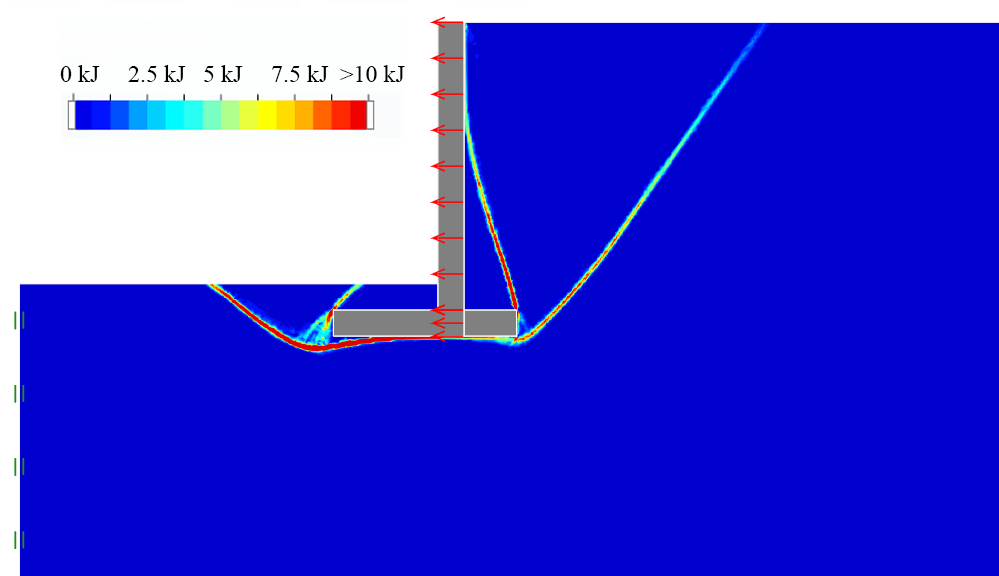


(a) Soil mass sliding surface


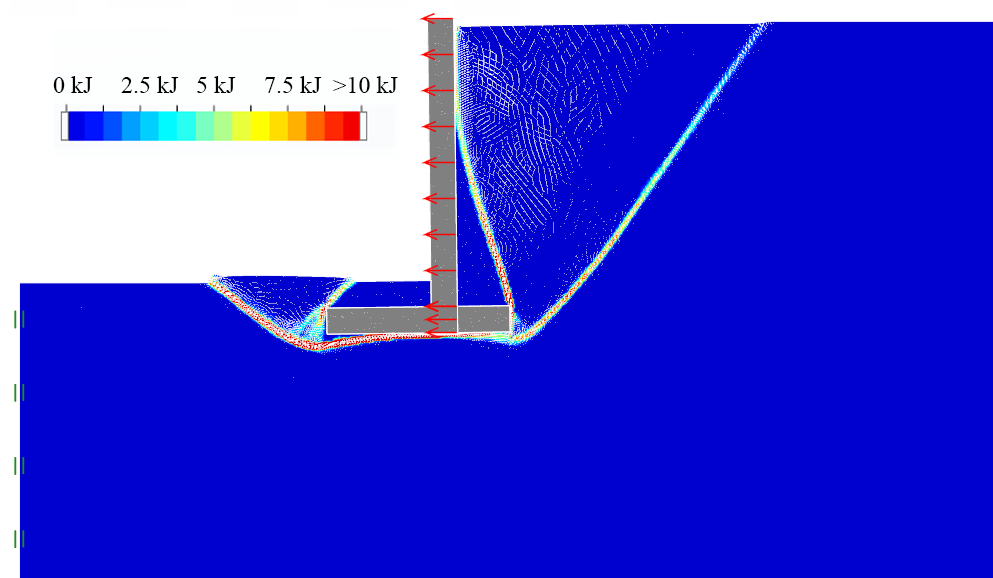


(b) Sliding process


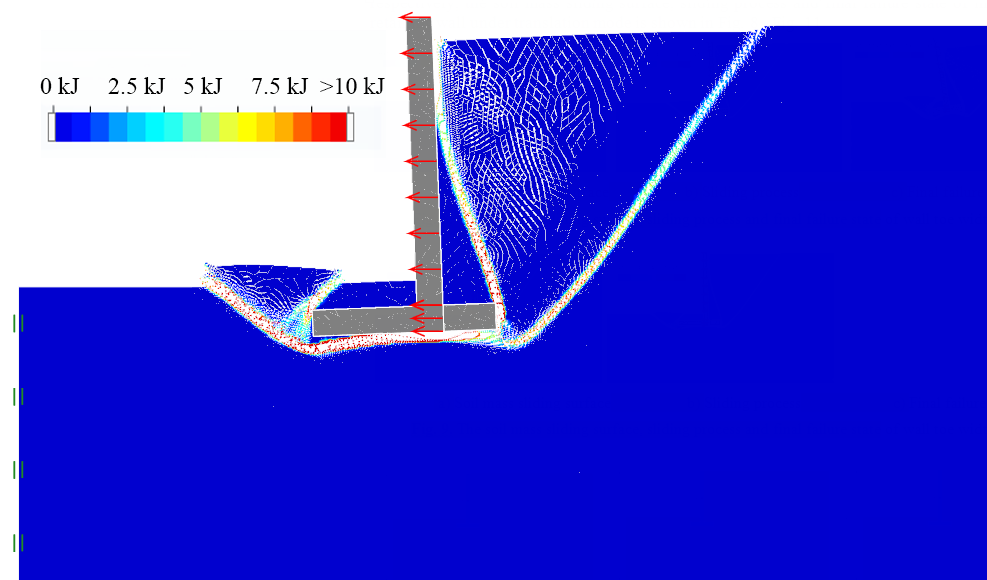


(c) Final failure state

**The soil mass sliding surface, sliding process and final failure state of wall toe width b_3_=2m.**


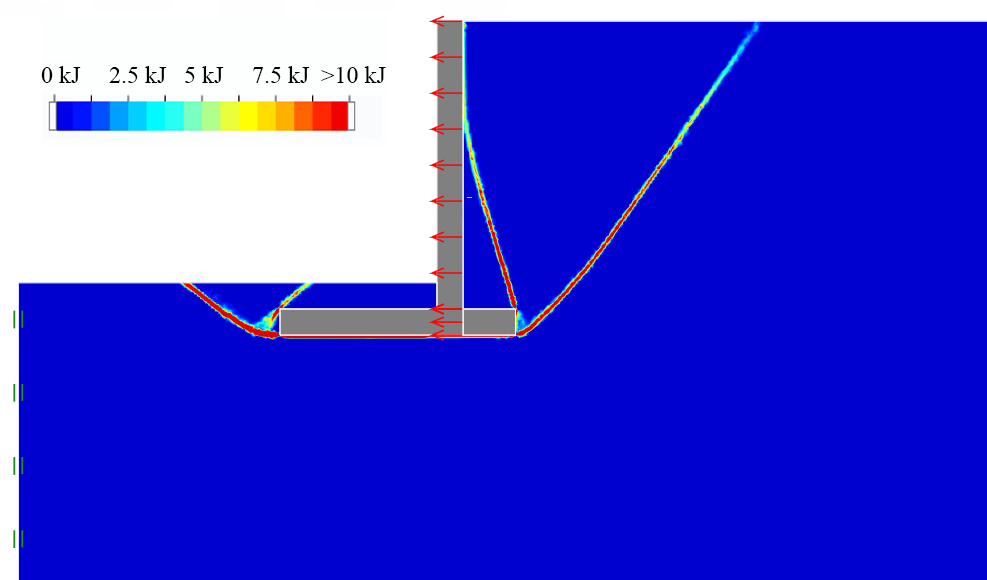


(a) Soil mass sliding surface


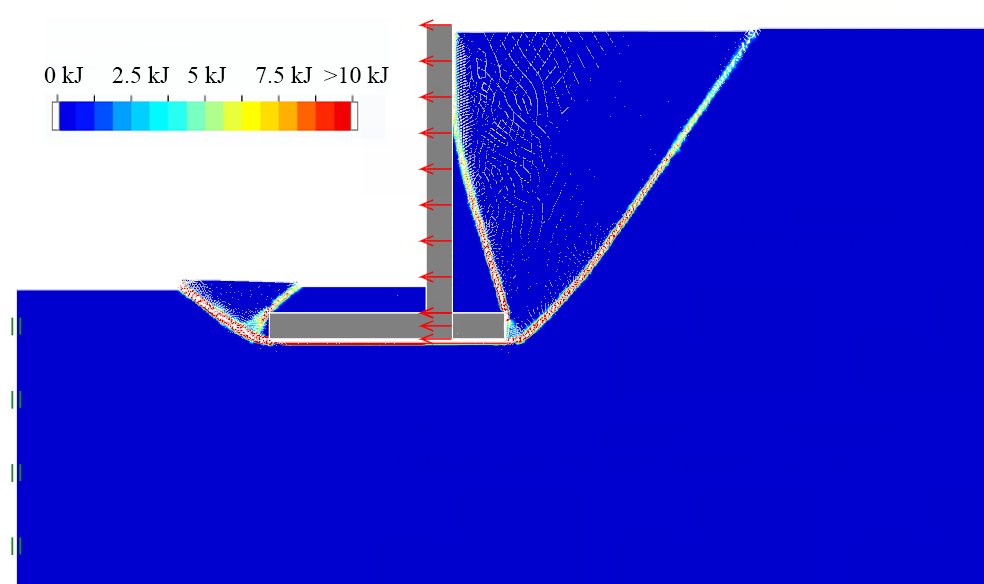


(b) Sliding process


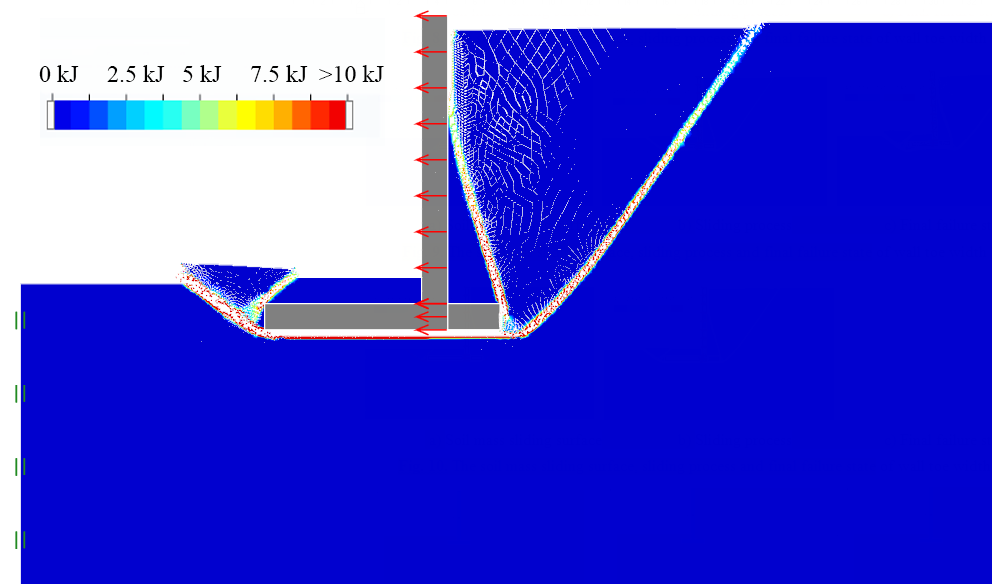


(c) Final failure state

**The soil mass sliding surface, sliding process and final failure state of wall toe width b_3_=3m.**


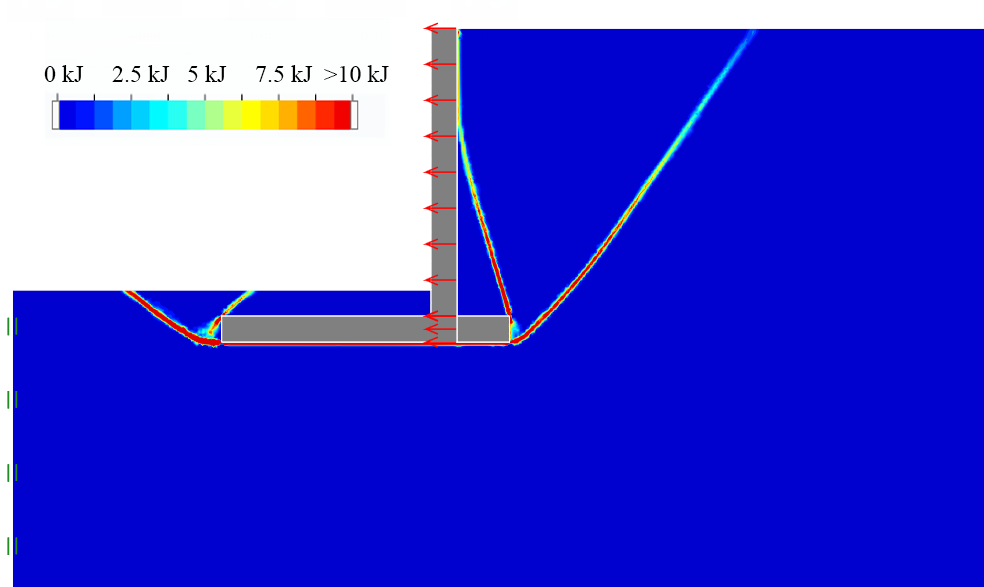


(a) Soil mass sliding surface


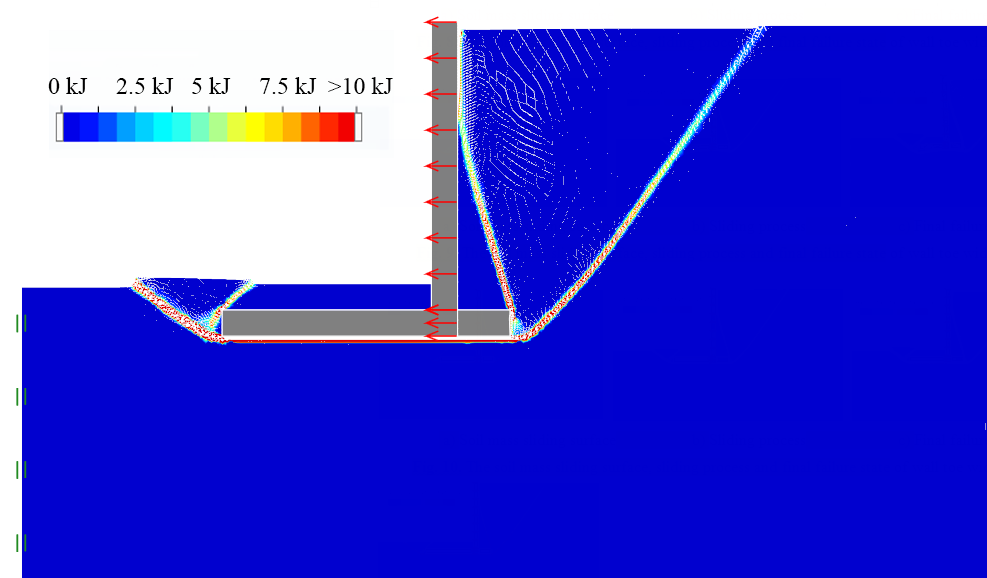


(b) Sliding process


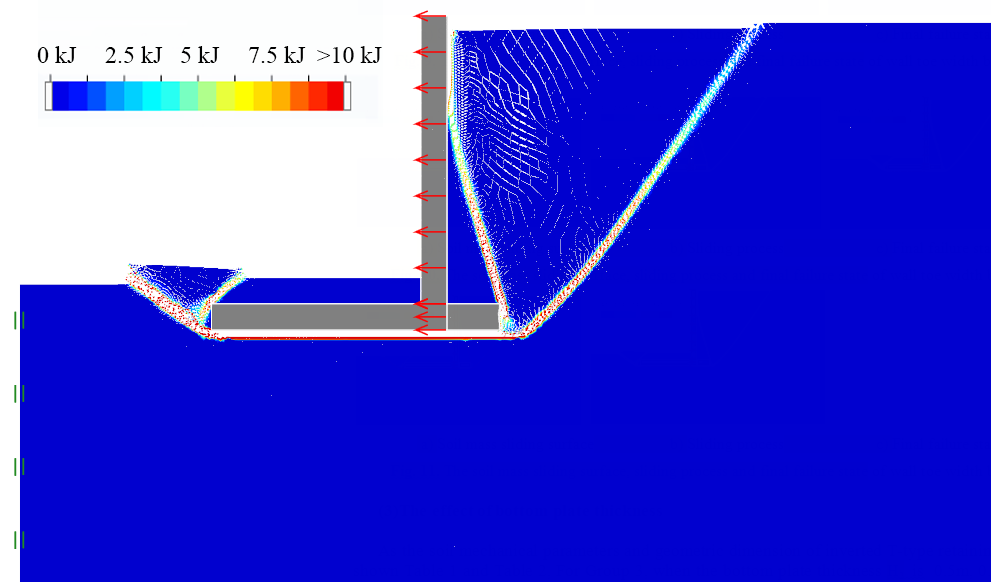


(c) Final failure state

**The soil mass sliding surface, sliding process and final failure state of wall toe width b_3_=4m.**

**(3) The effect of** **bottom plate thickness on the soil mass sliding surface, sliding process and final failure state**


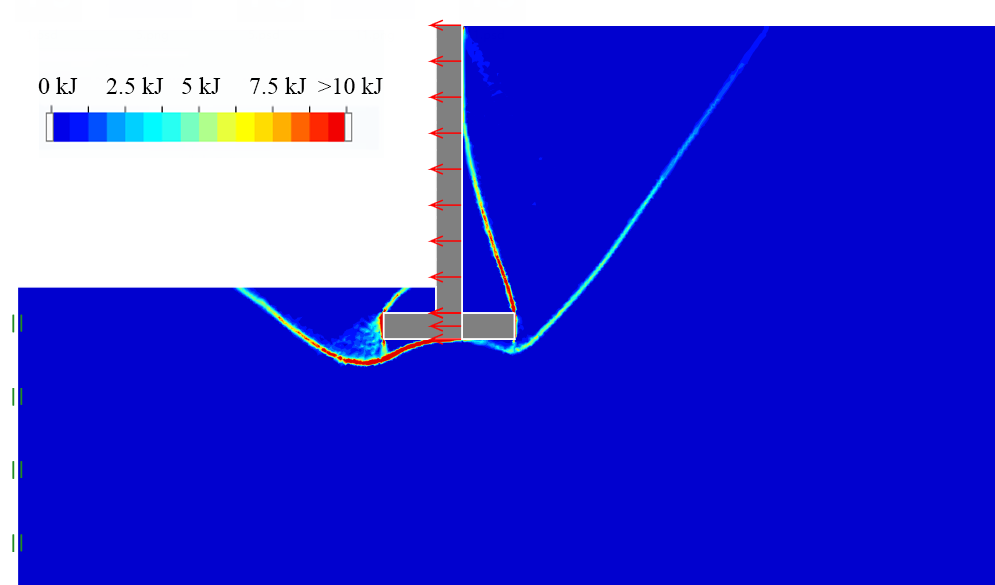


(a) Soil mass sliding surface


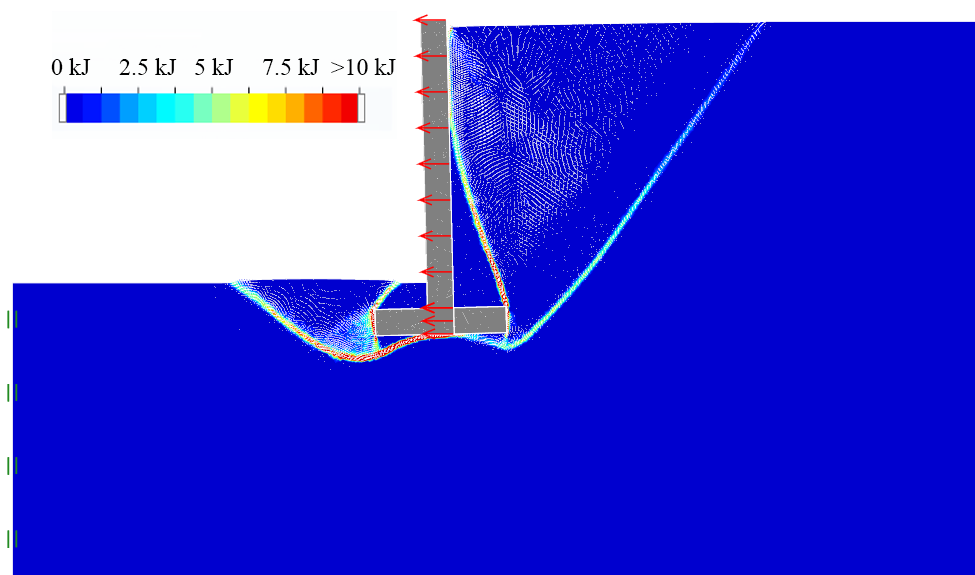


(b) Sliding process


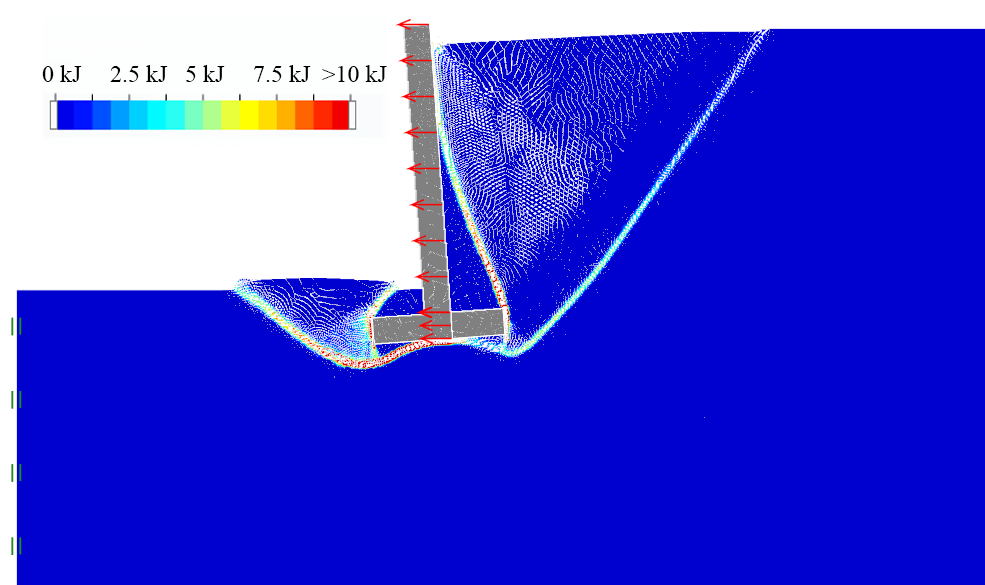


(c) Final failure state

**The soil mass sliding surface, sliding process and final failure state of bottom plate thicknesss H_2_ =0.5m.**


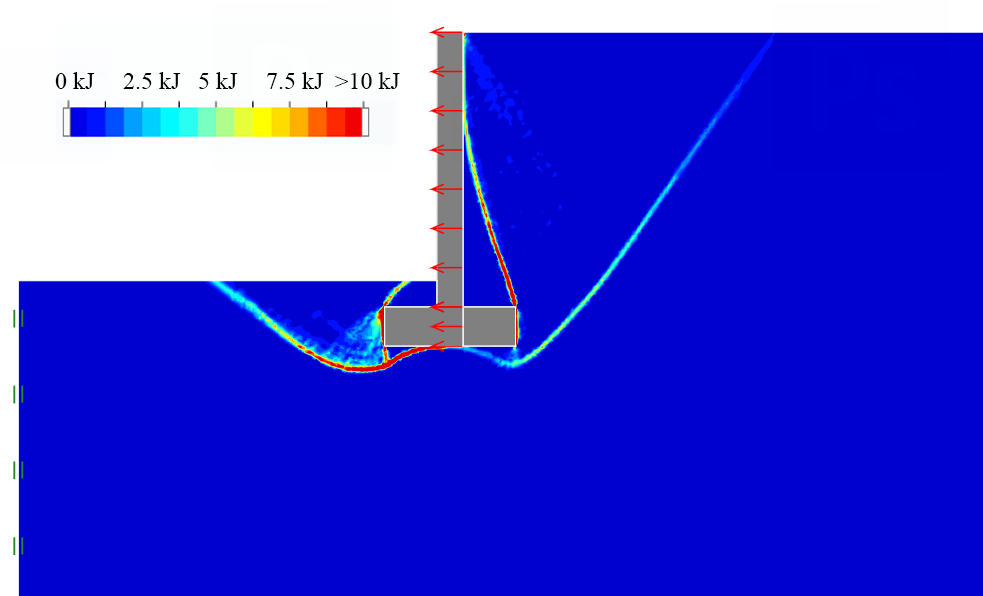


(a) Soil mass sliding surface


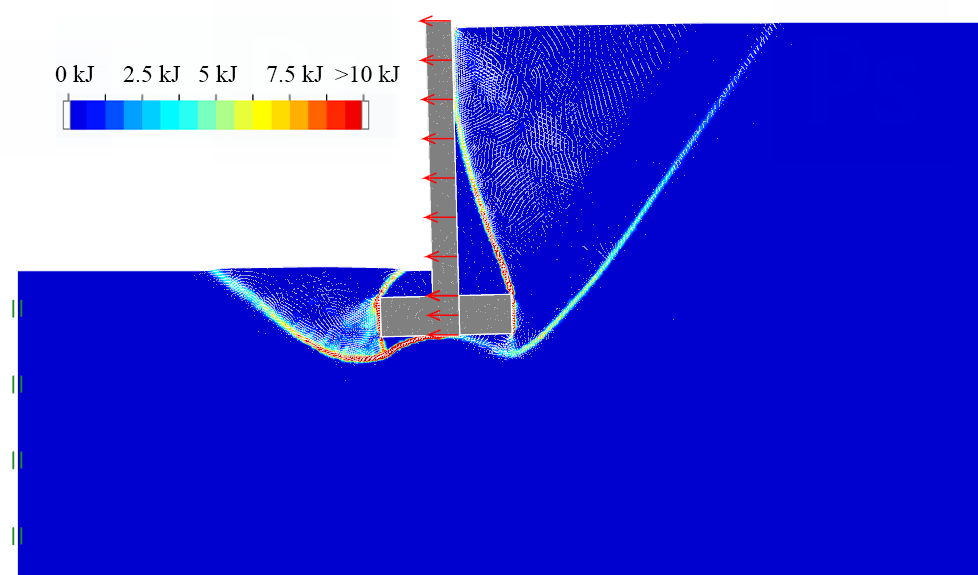


(b) Sliding process


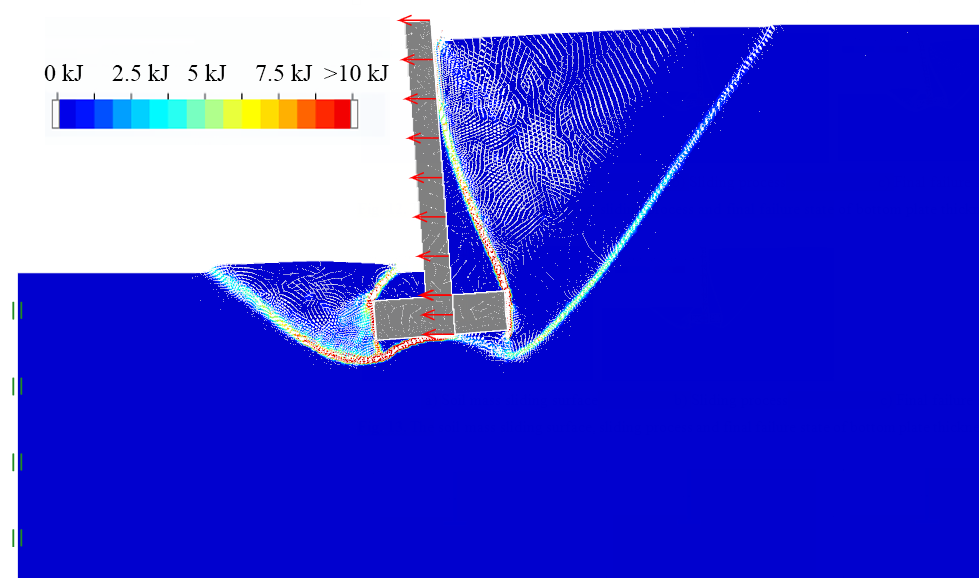


(c) Final failure state

**The soil mass sliding surface, sliding process and final failure state of bottom plate thicknesss H_2_ =0.75m.**


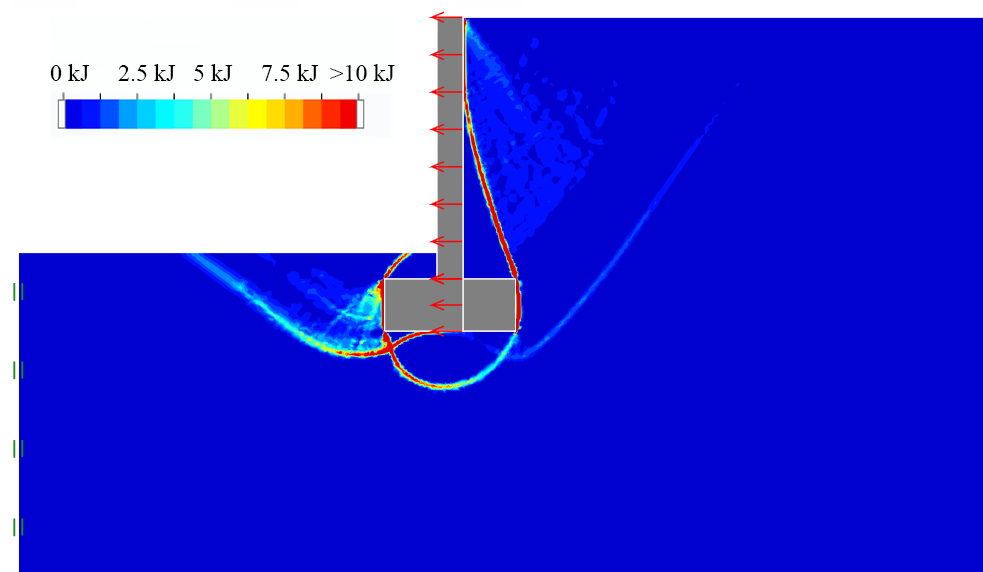


(a) Soil mass sliding surface


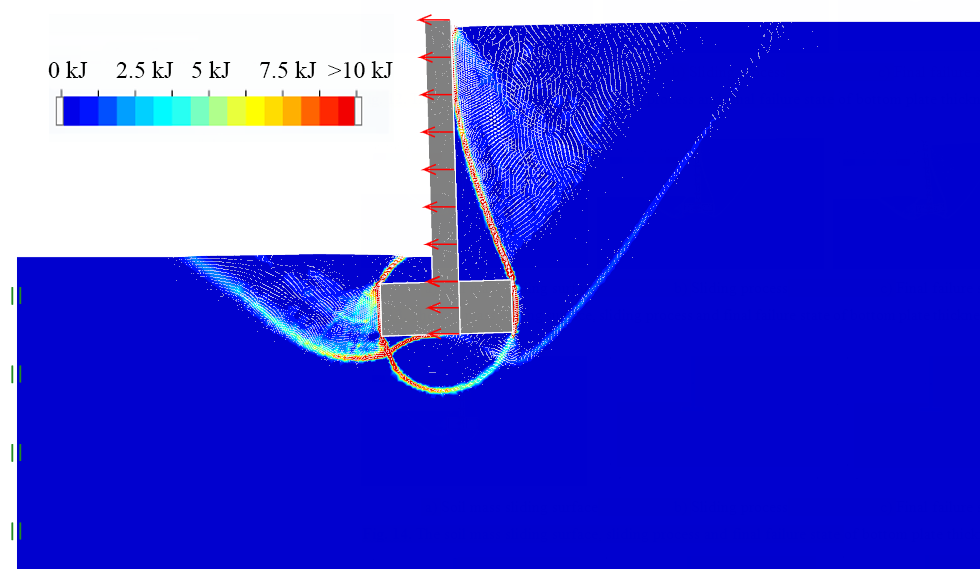


(b) Sliding process


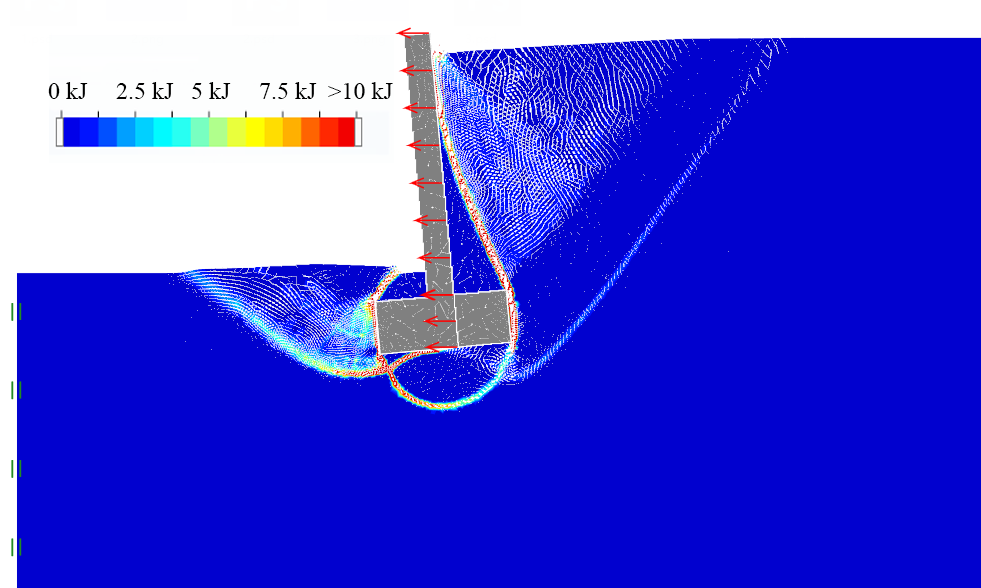


(c) Final failure state

**The soil mass sliding surface, sliding process and final failure state of bottom plate thicknesss H_2_ =1m.**


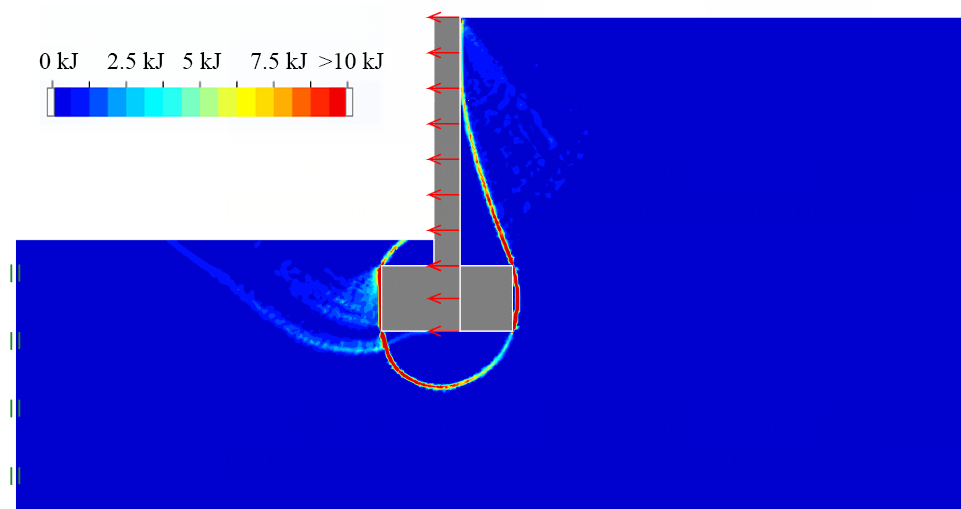


(a) Soil mass sliding surface


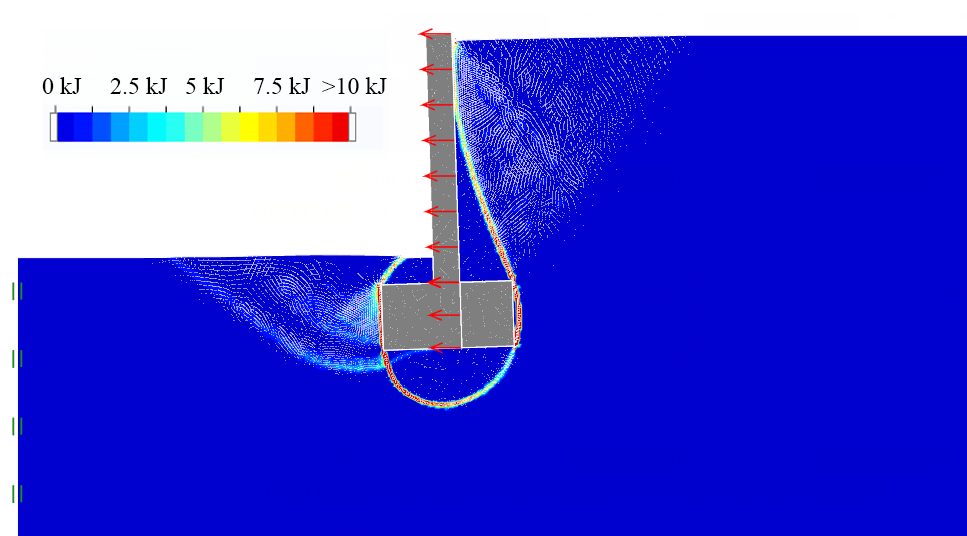


(b) Sliding process


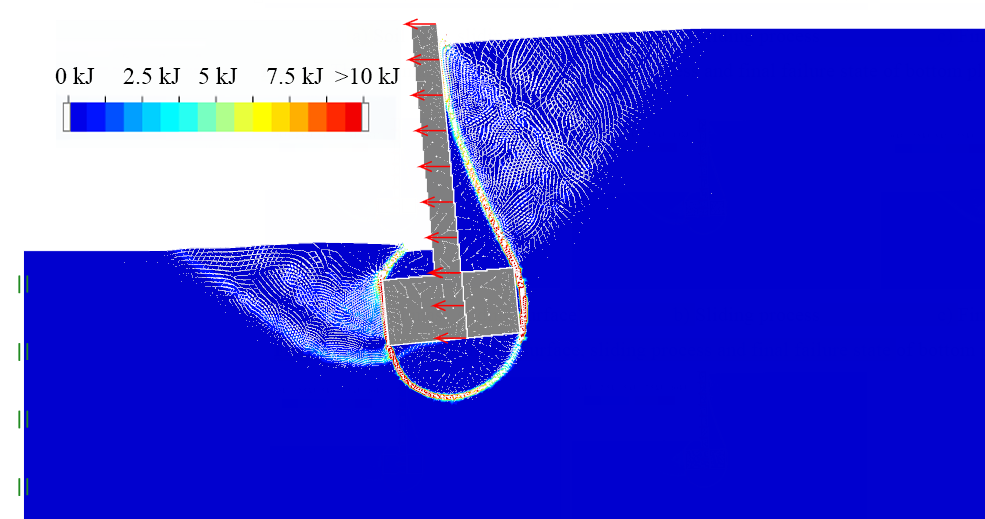


(c) Final failure state

**The soil mass sliding surface, sliding process and final failure state of bottom plate thicknesss H_2_ =1.25m.**

**(4) The effect of soil–wall interface element reduction coefficient on the soil mass sliding surface, sliding process and final failure state**


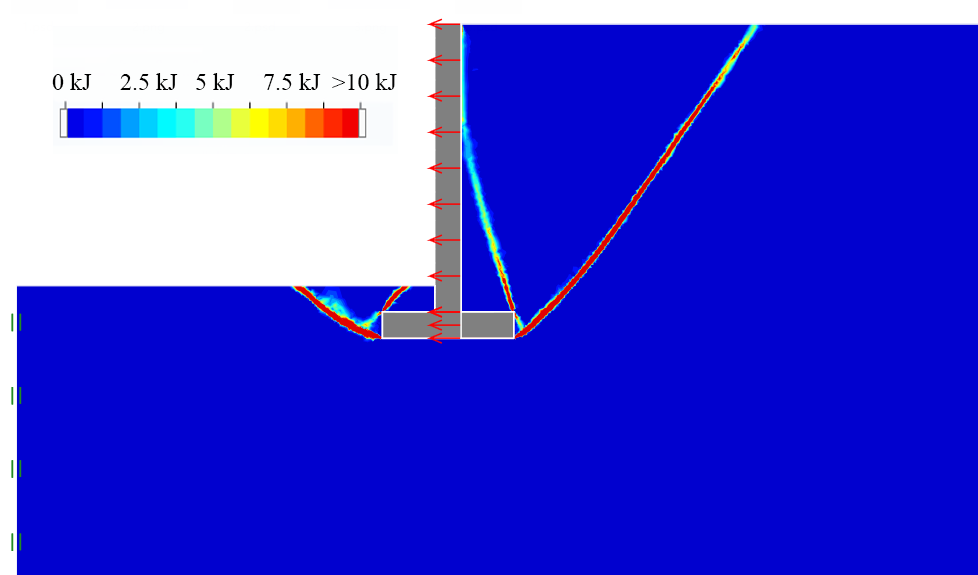


(a) Soil mass sliding surface


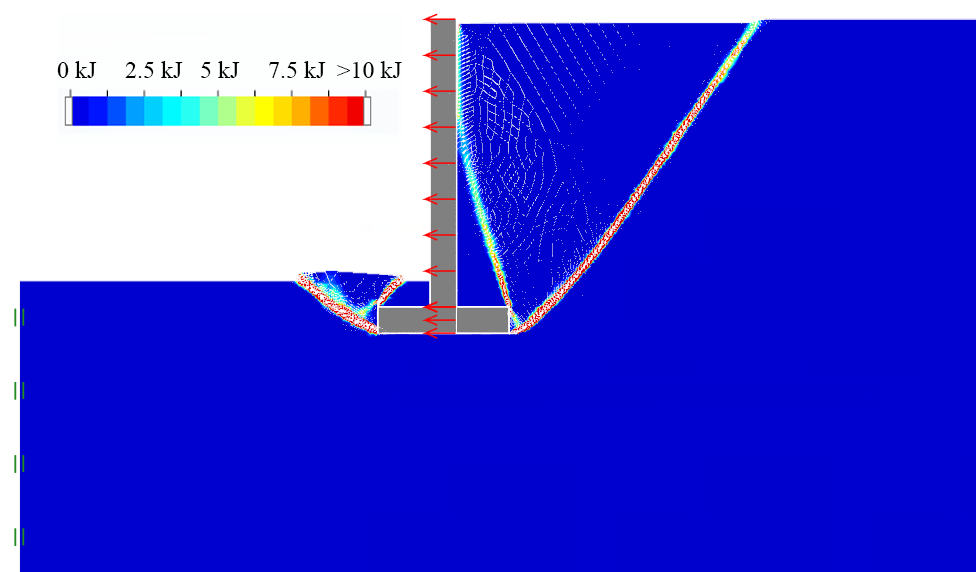


(b) Sliding process


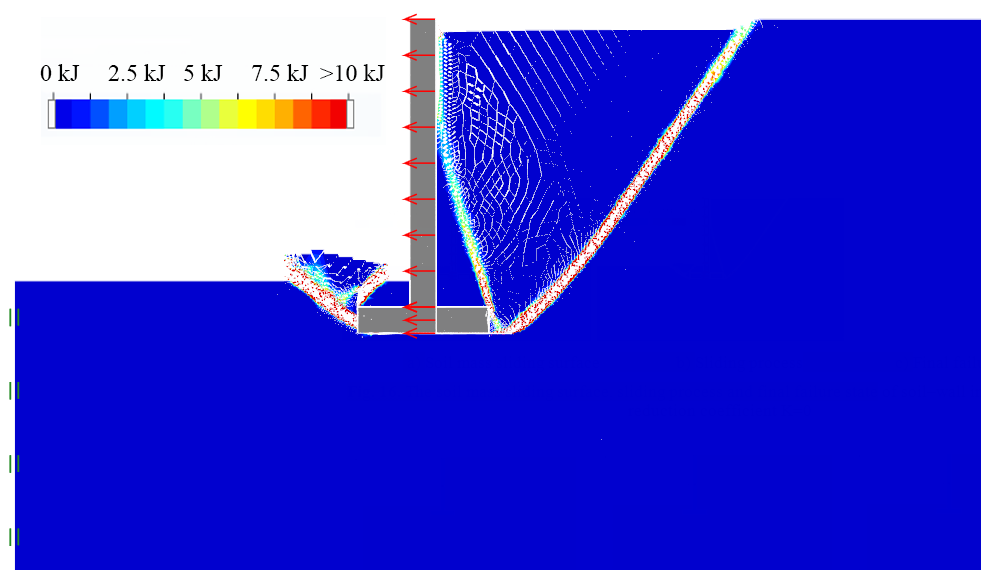


(c) Final failure state

**The soil mass sliding surface, sliding process and final failure state of soil–wall interface element reduction coefficient K=0.**


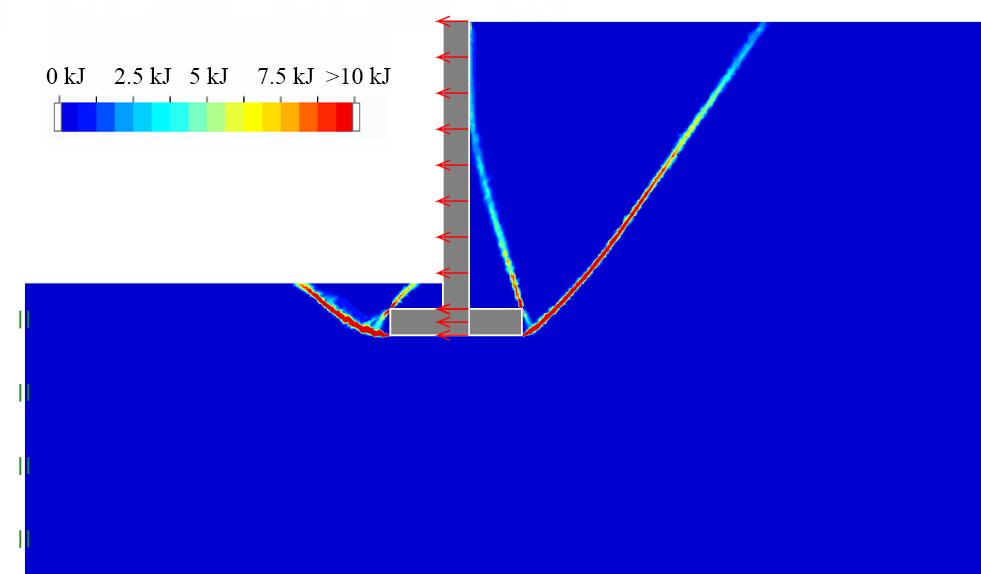


(a) Soil mass sliding surface


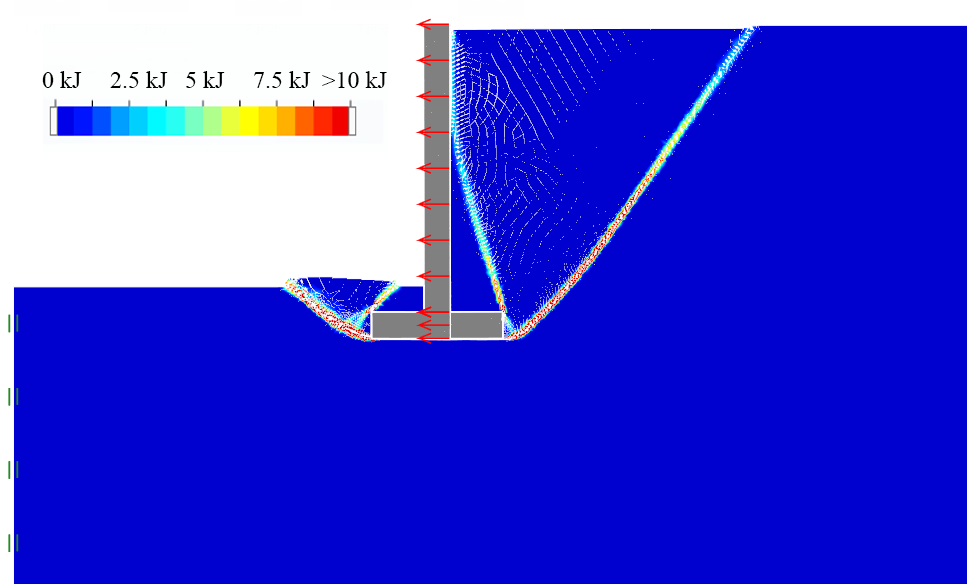


(b) Sliding process


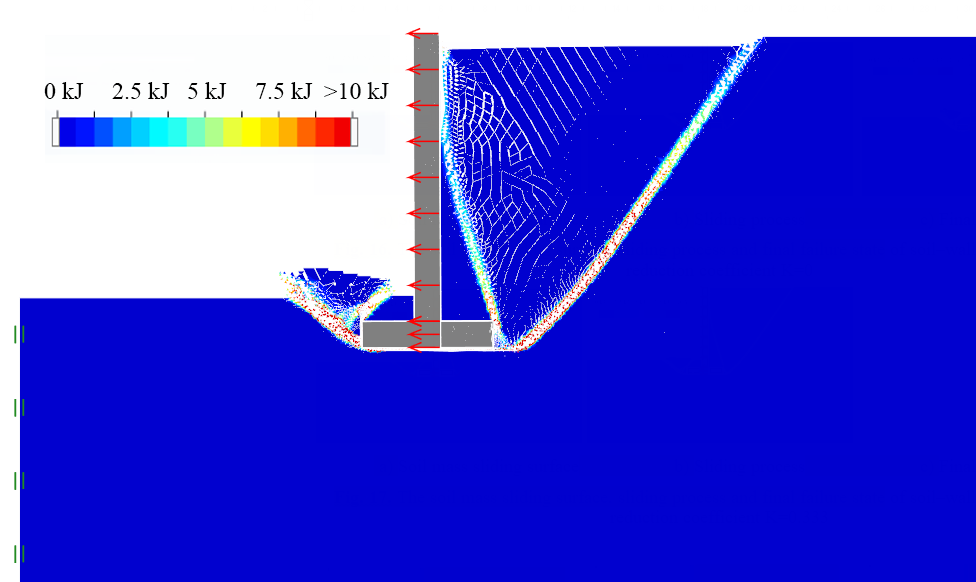


(c) Final failure state

**The soil mass sliding surface, sliding process and final failure state of soil–wall interface element reduction coefficient K=0.333.**


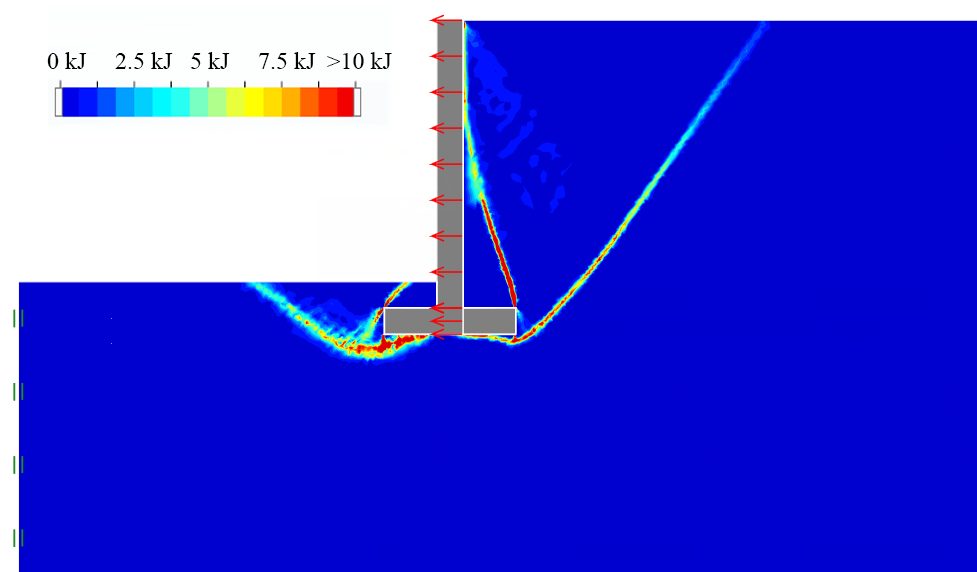


(a) Soil mass sliding surface


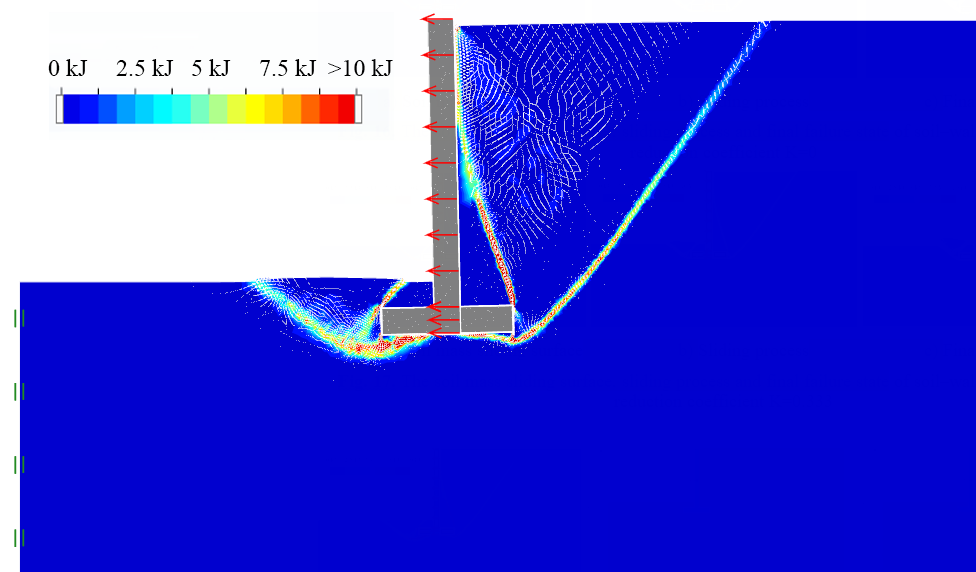


(b) Sliding process


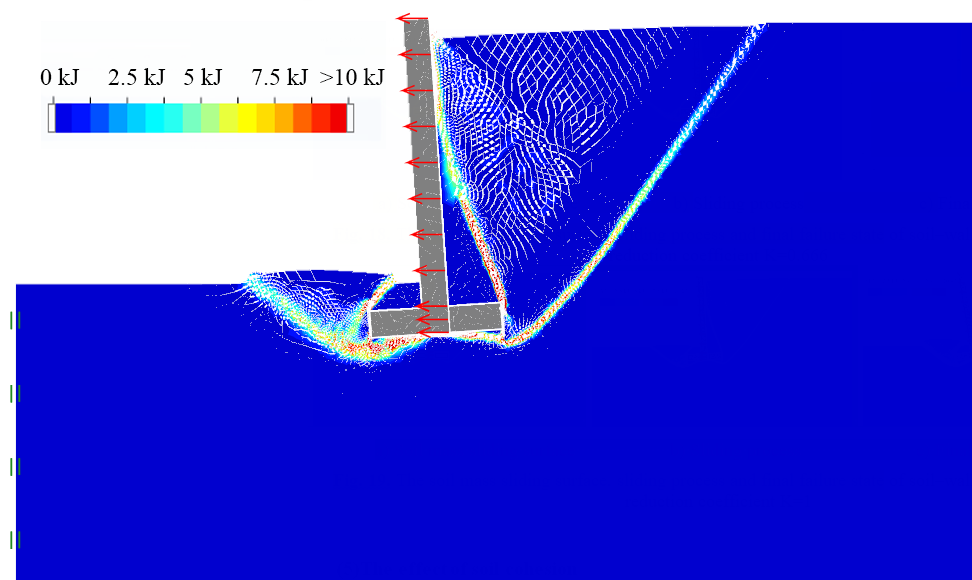


(c) Final failure state

**The soil mass sliding surface, sliding process and final failure state of soil–wall interface element reduction coefficient K=0.666.**


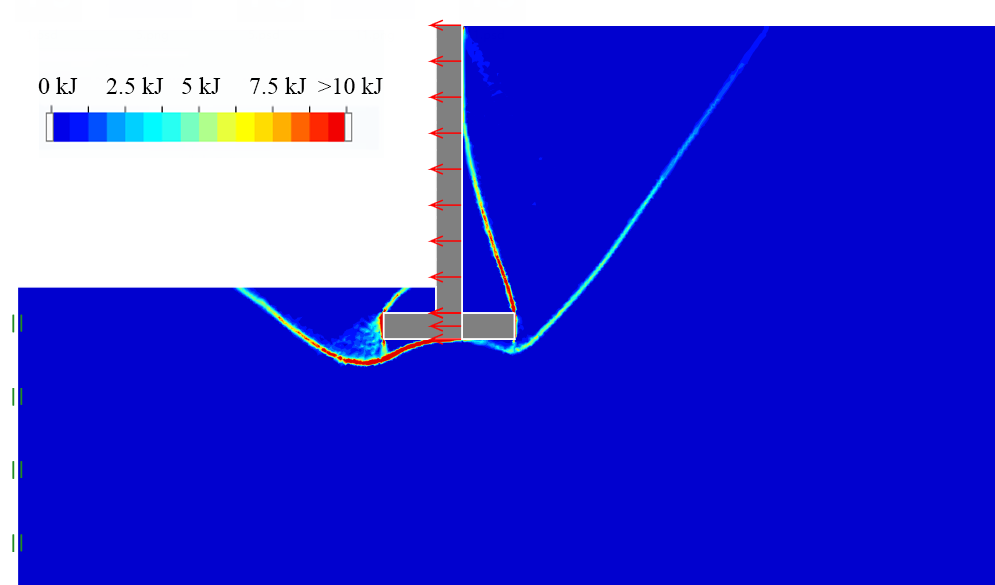


(a) Soil mass sliding surface


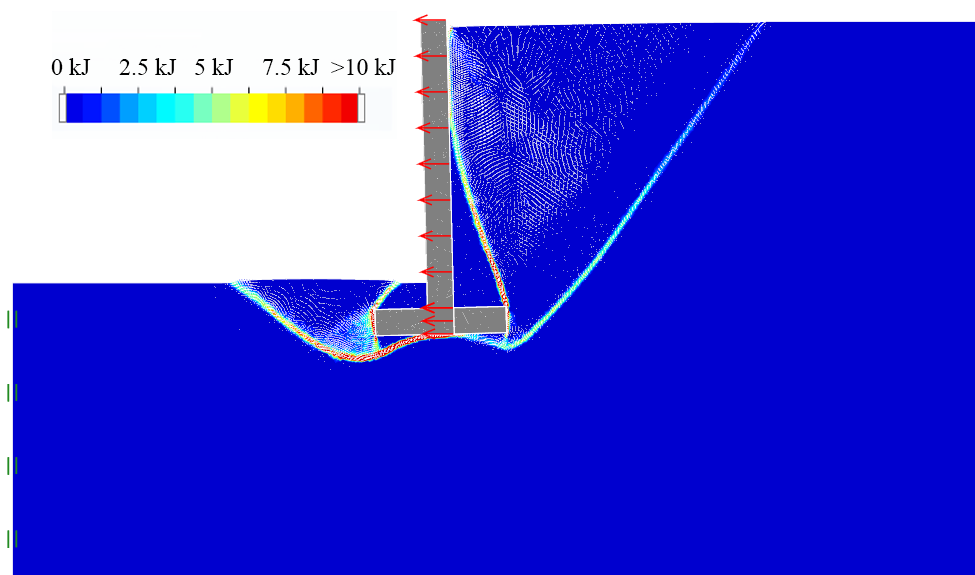


(b) Sliding process


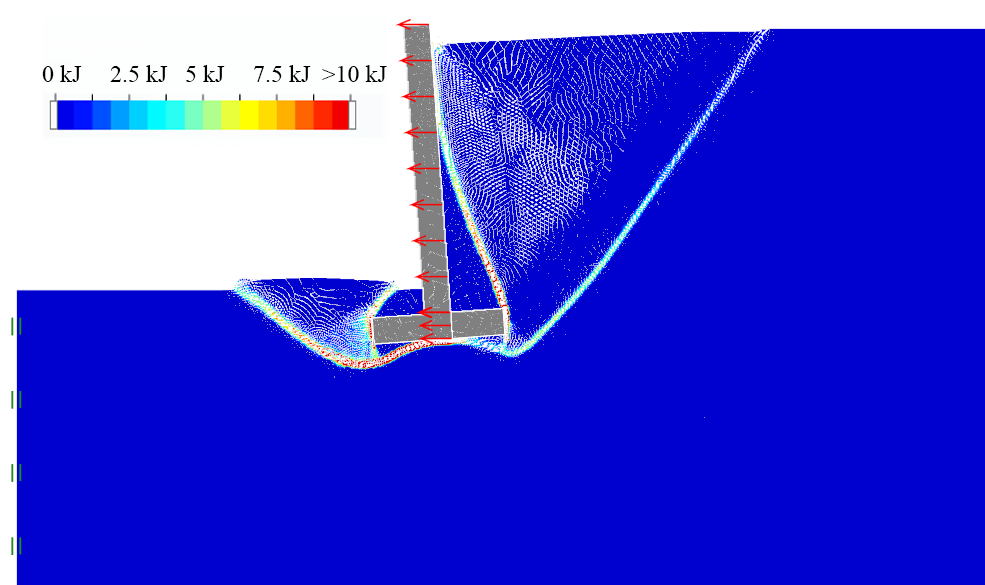


(c) Final failure state

**The soil mass sliding surface, sliding process and final failure state of soil–wall interface element reduction coefficient K=1.**

**(5) The effect of soil cohesion on the soil mass sliding surface, sliding process and final failure state**


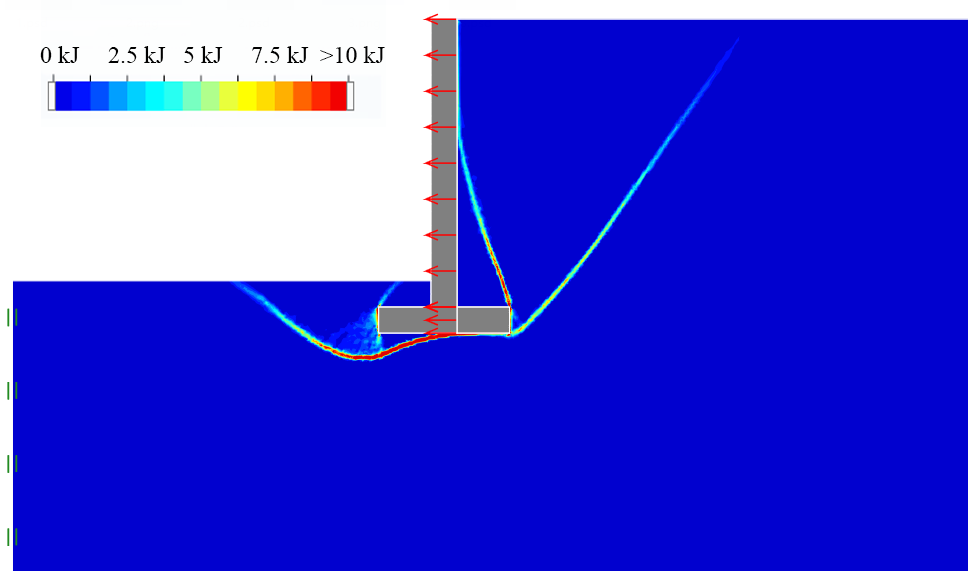


(a) Soil mass sliding surface


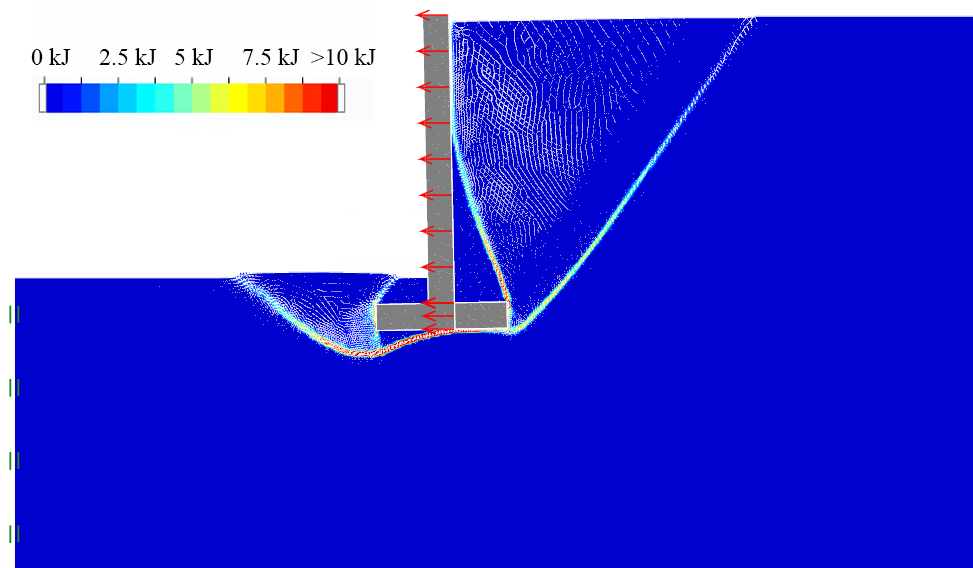


(b) Sliding process


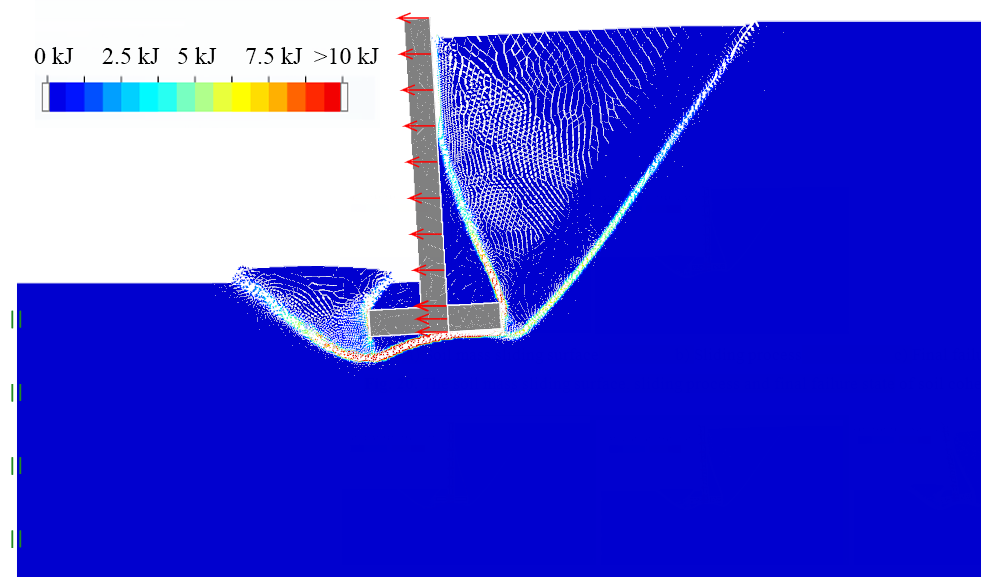


(c) Final failure state

**The soil mass sliding surface, sliding process and final failure state of soil cohesion C=5KPa.**


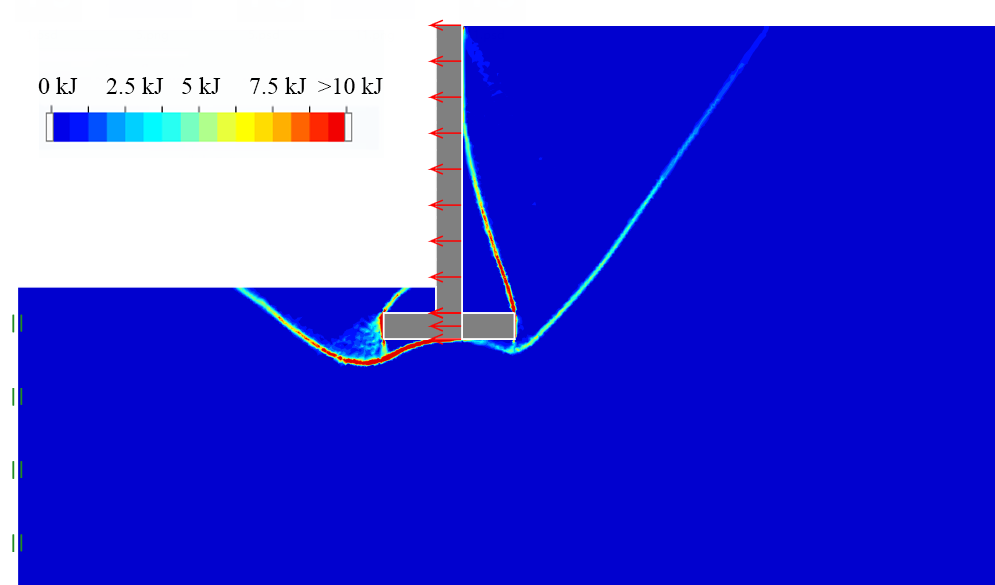


(a) Soil mass sliding surface


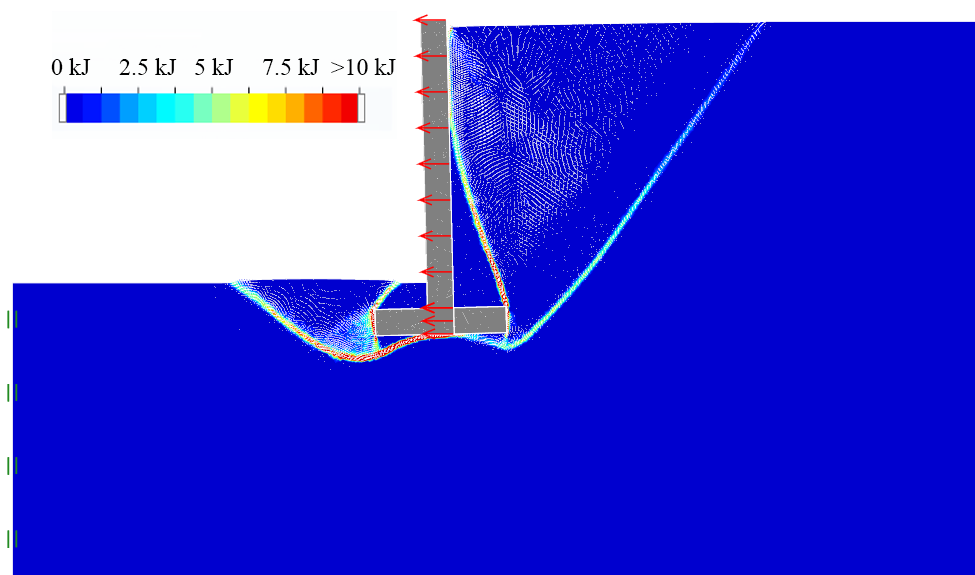


(b) Sliding process


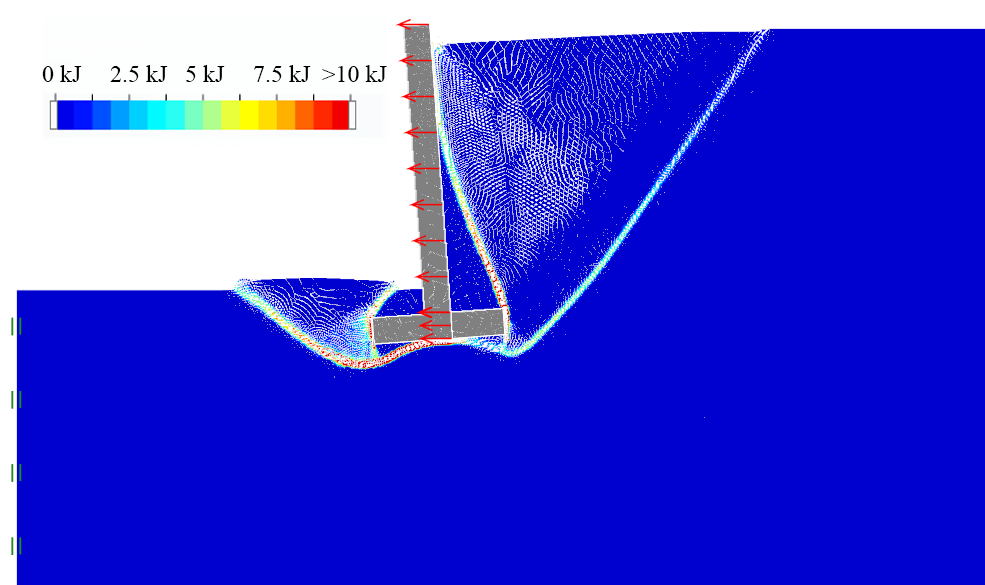


(c) Final failure state

**The soil mass sliding surface, sliding process and final failure state of soil cohesion C=15KPa.**


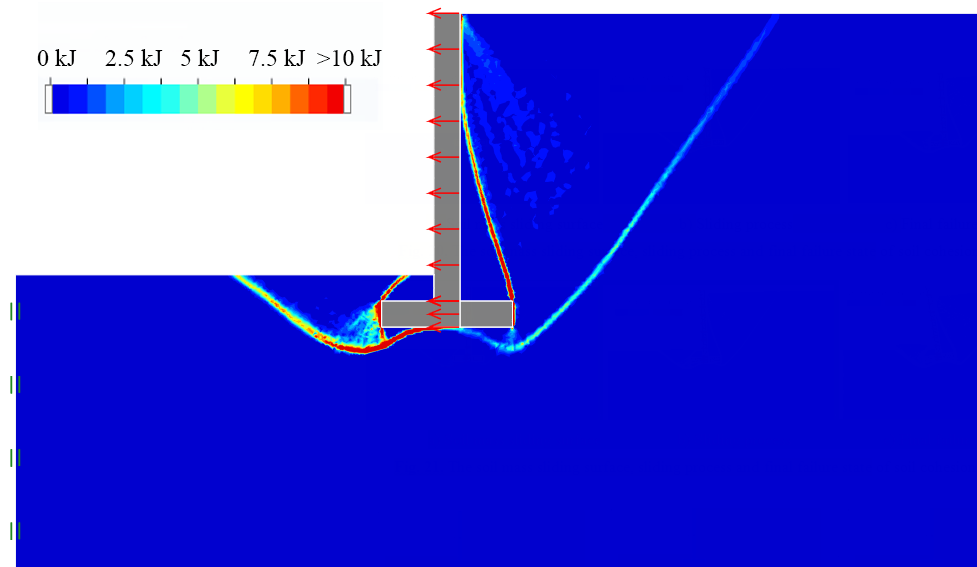


(a) Soil mass sliding surface


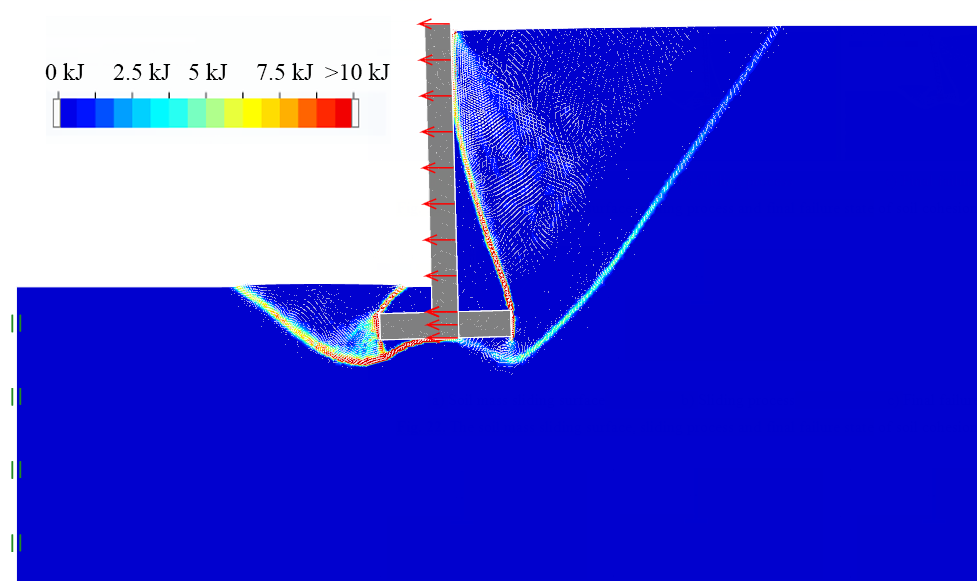


(b) Sliding process


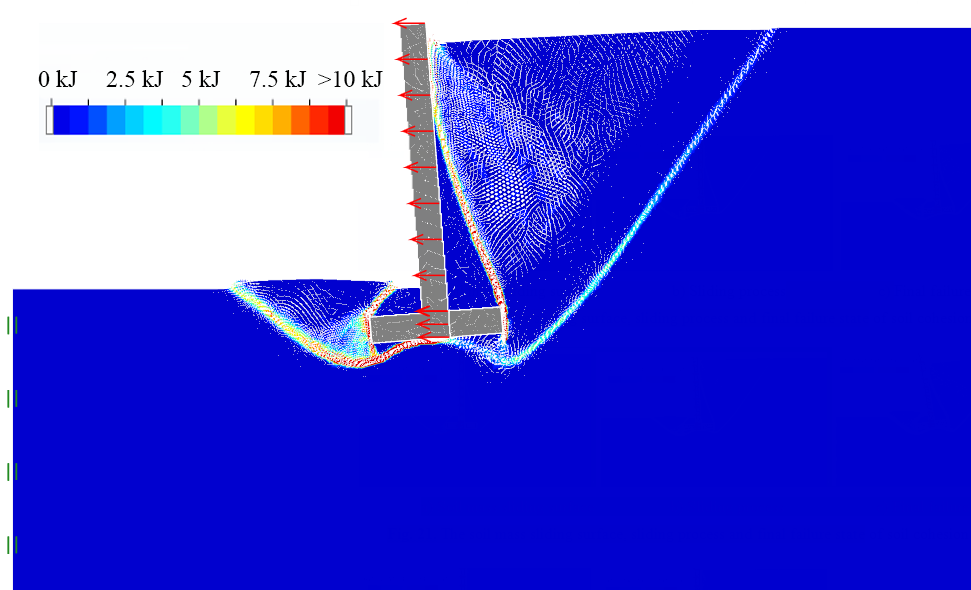


(c) Final failure state

**The soil mass sliding surface, sliding process and final failure state of soil cohesion C=25KPa.**


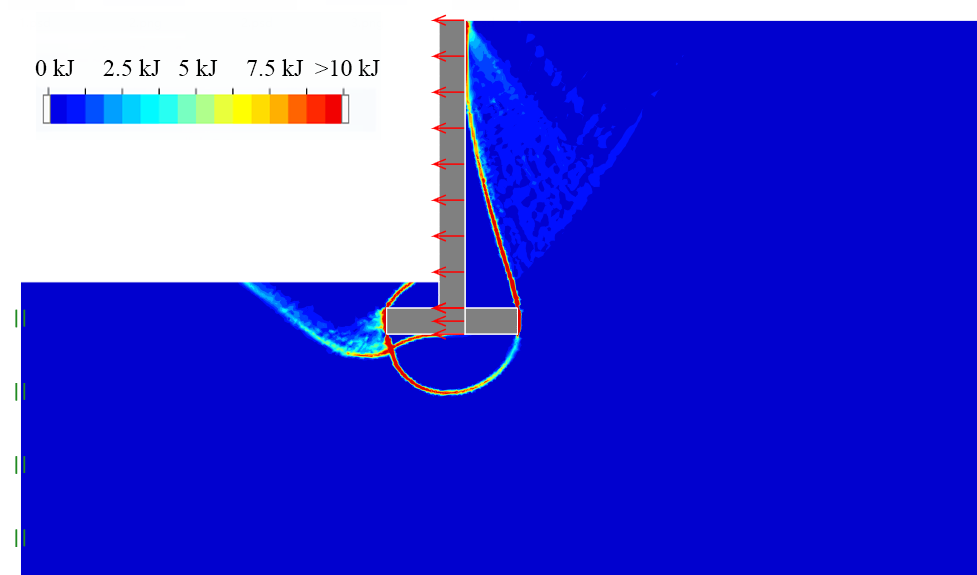


(a) Soil mass sliding surface


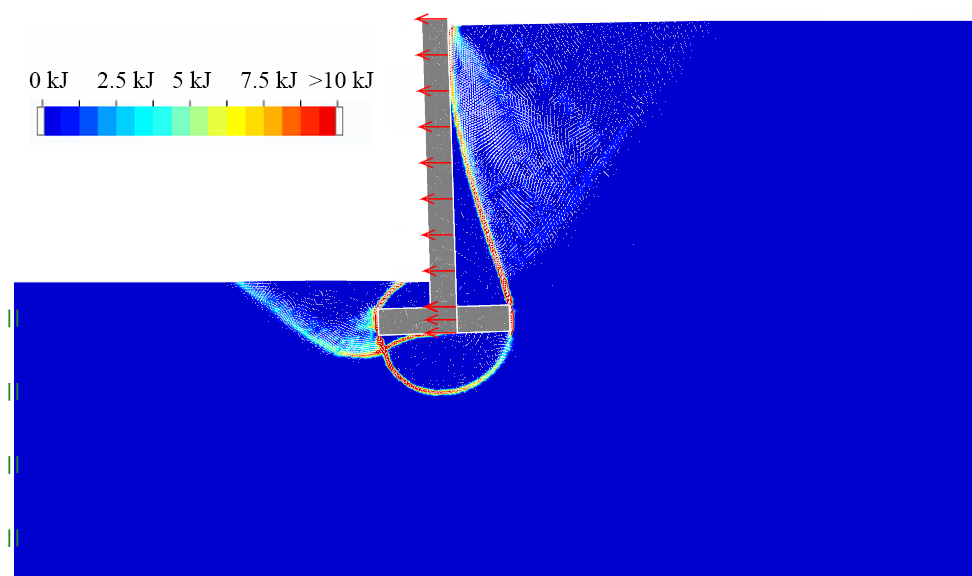


(b) Sliding process


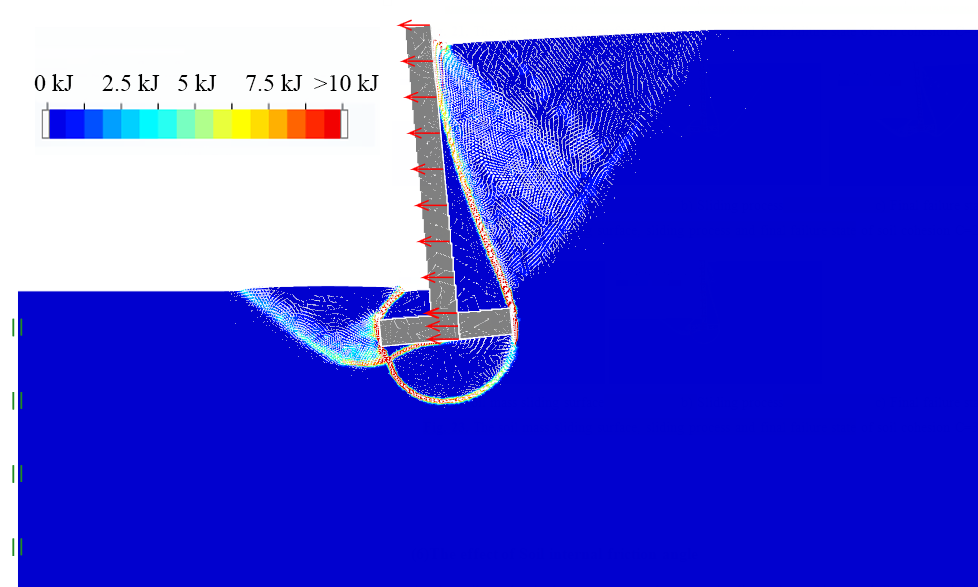


(c) Final failure state

**The soil mass sliding surface, sliding process and final failure state of soil cohesion C=35KPa.**

**(6) The effect of soil internal friction angle on the soil mass sliding surface, sliding process and final failure state**


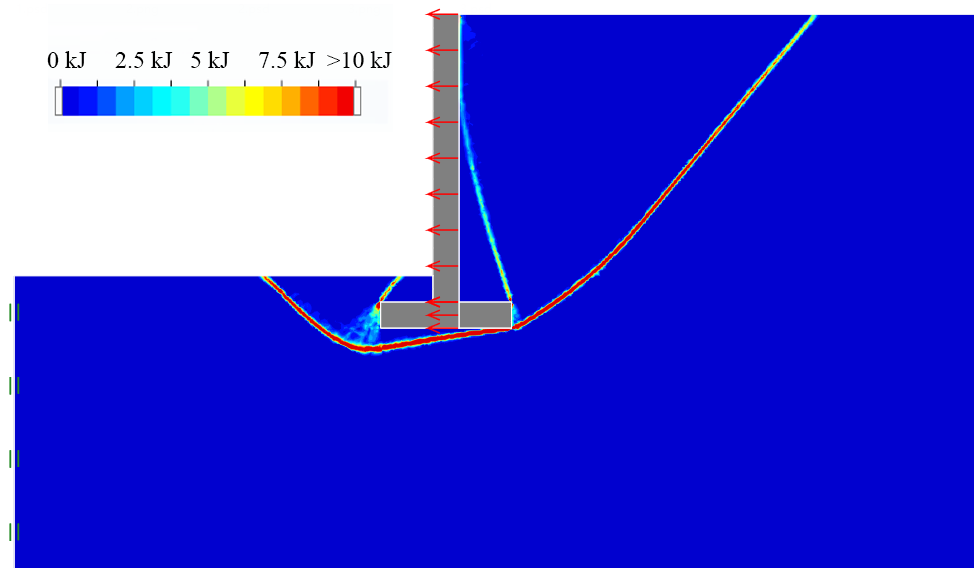


(a) Soil mass sliding surface


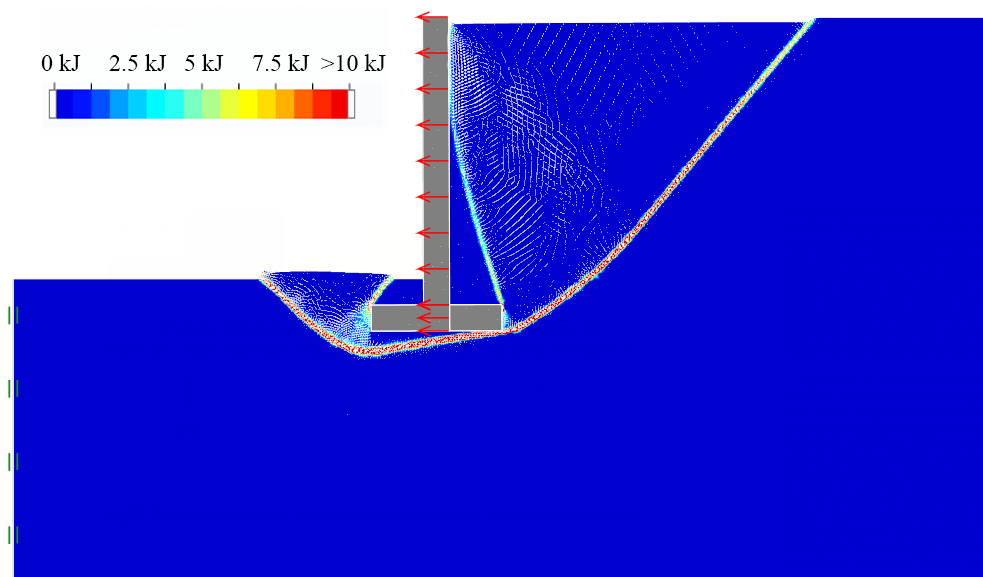


(b) Sliding process


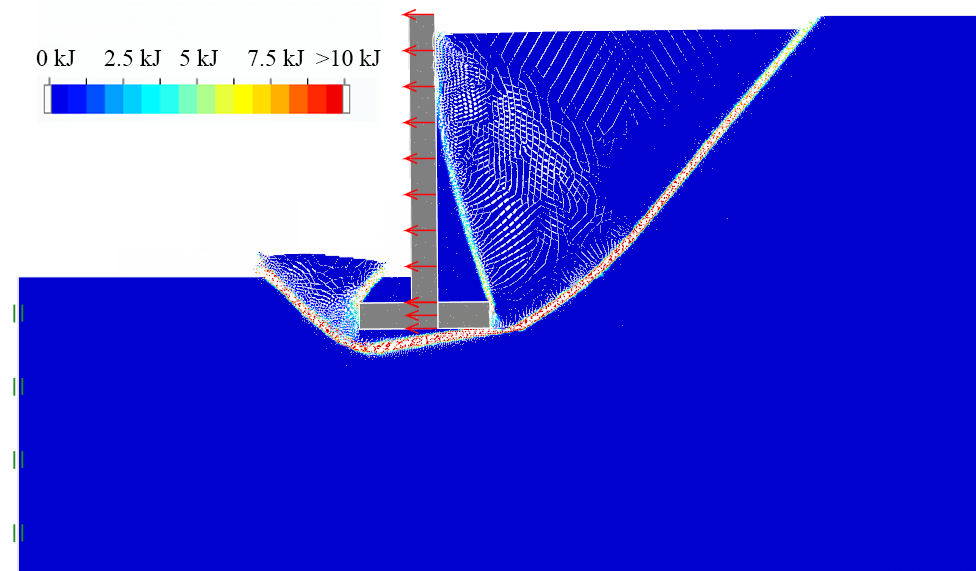


(c) Final failure state

**The soil mass sliding surface, sliding process and final failure state of soil internal friction angle φ =10^o^.**


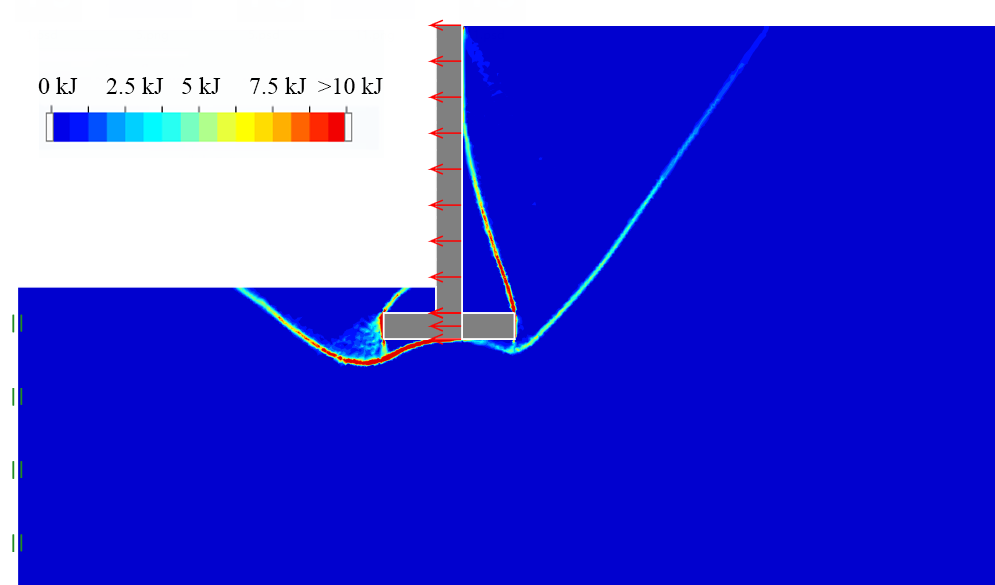


(a) Soil mass sliding surface


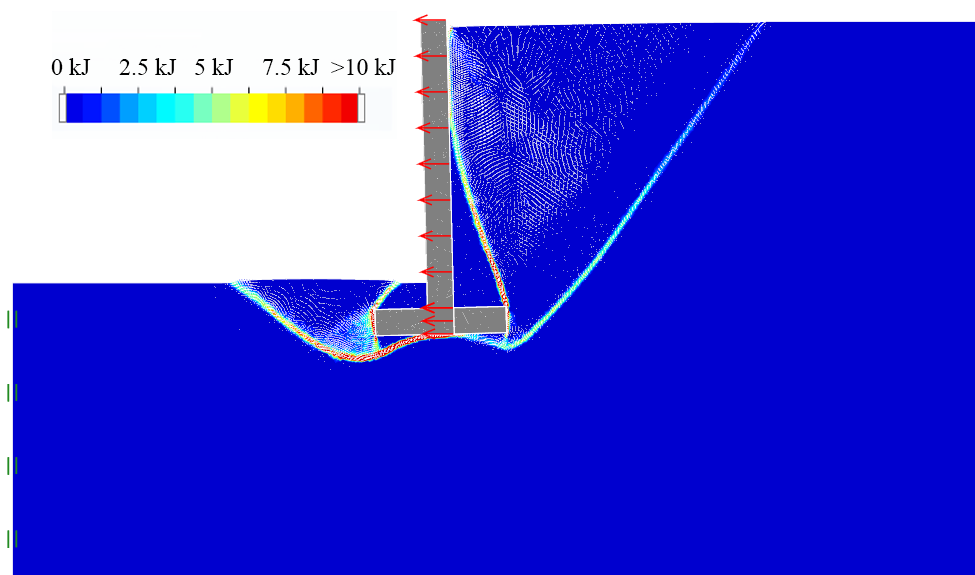


(b) Sliding process


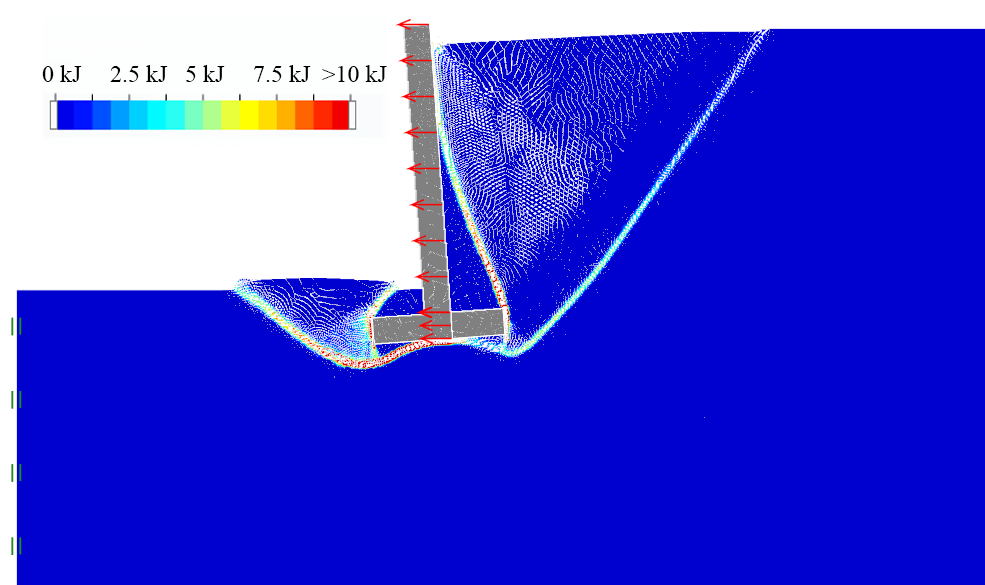


(c) Final failure state

**The soil mass sliding surface, sliding process and final failure state of soil internal friction angle φ =20^o^.**


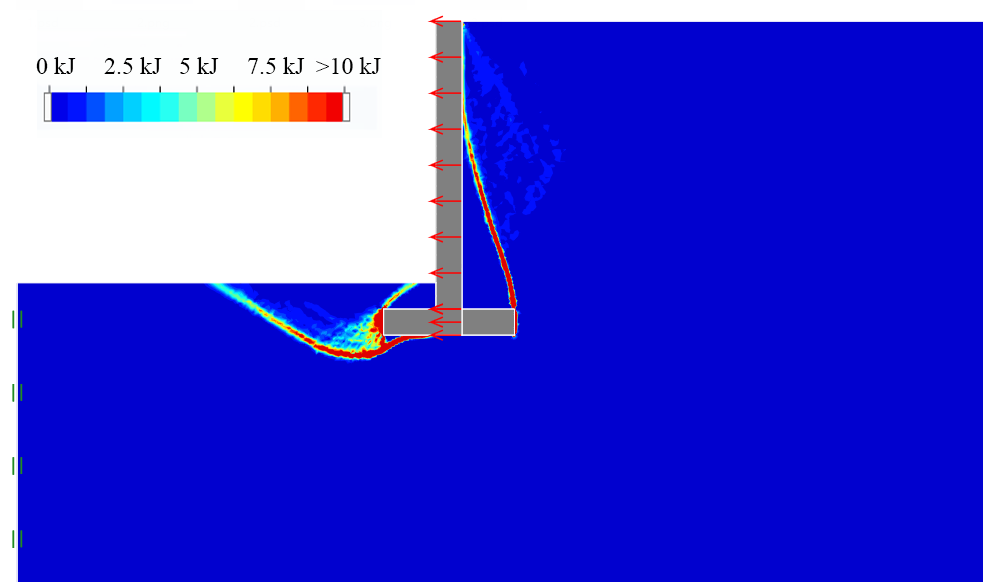


(a) Soil mass sliding surface


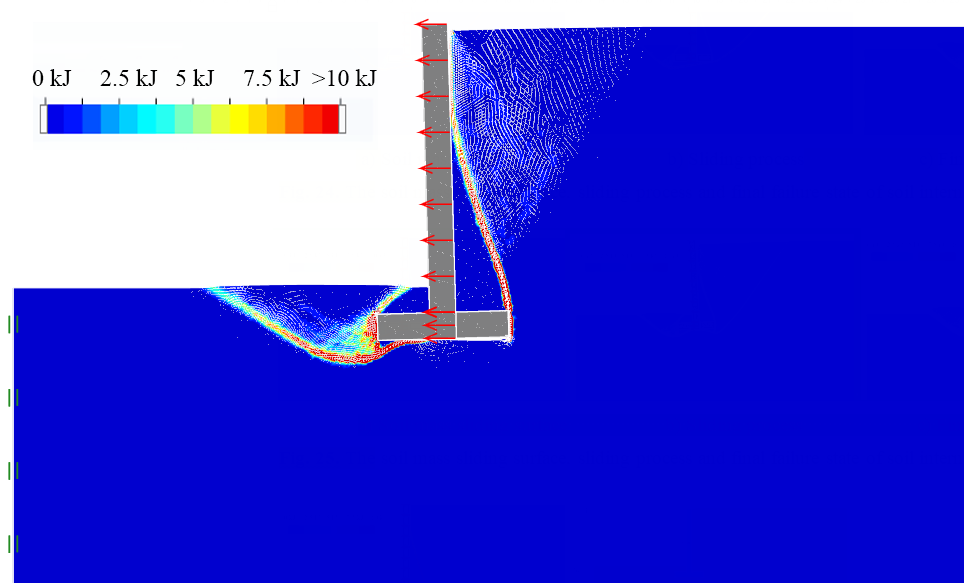


(b) Sliding process


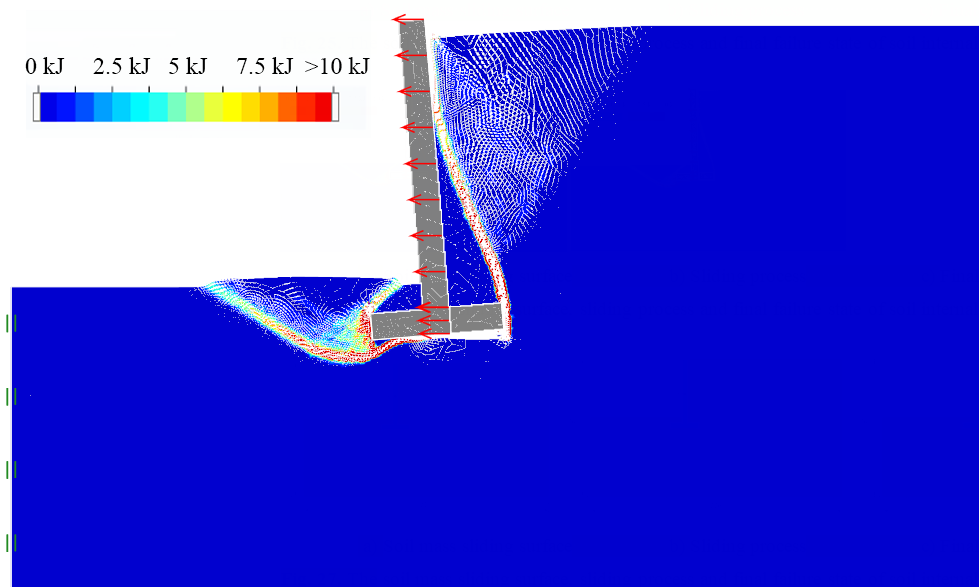


(c) Final failure state

**The soil mass sliding surface, sliding process and final failure state of soil internal friction angle φ =30^o^.**


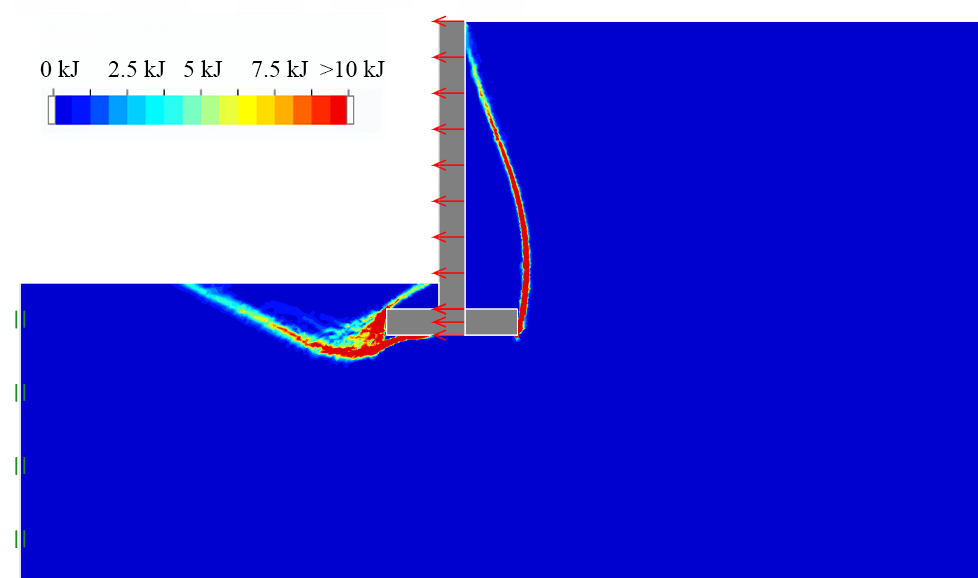


(a) Soil mass sliding surface


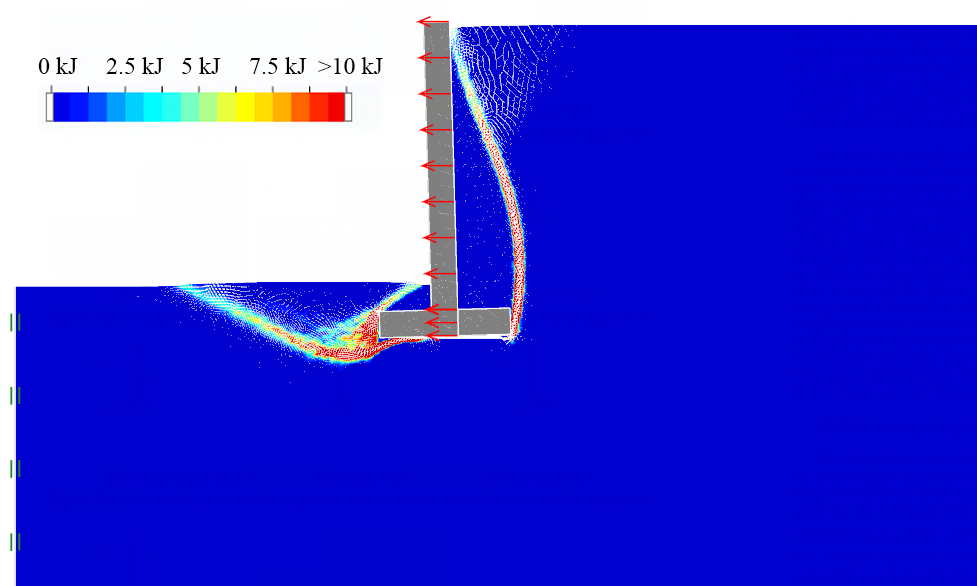


(b) Sliding process


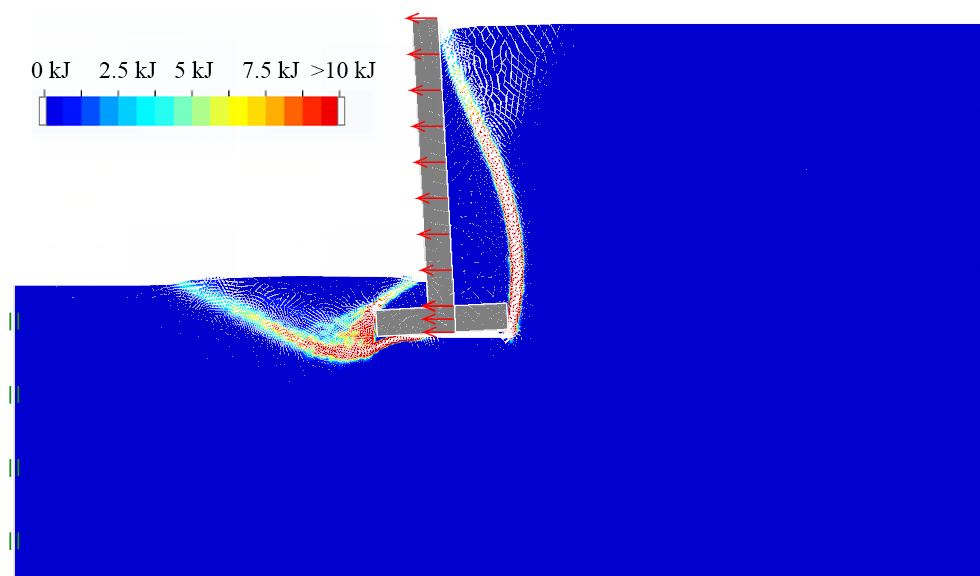


(c) Final failure state

**The soil mass sliding surface, sliding process and final failure state of soil internal friction angle φ =40^o^.**
